# Supramolecular Assemblies of Self‐Immolative Janus Dendrimers With Rapid Photodegradation Response

**DOI:** 10.1002/smll.202511067

**Published:** 2026-02-05

**Authors:** Chuanfeng Li, Jiabin Luan, Daniela A. Wilson, Elizabeth R. Gillies

**Affiliations:** ^1^ Department of Chemistry The University of Western Ontario London Ontario Canada; ^2^ Institute for Molecules and Materials Radboud University Nijmegen The Netherlands; ^3^ Department of Chemical and Biochemical Engineering The University of Western Ontario London Ontario Canada

**Keywords:** degradation, Janus dendrimer, self‐assembly, self‐immolative, supramolecular chemistry

## Abstract

Advanced supramolecular assemblies with predefined lifetimes and rapid responses to stimuli are in high demand for applications such as biomedical delivery systems. However, such assemblies are rarely able to respond rapidly and completely to stimuli, with predictable changes in morphology. Here, we introduce monodisperse self‐immolative Janus dendrimers (SIJDs) composed of hydrophilic oligo(ethylene glycol)‐functionalized phenolic acid dendrons and hydrophobic monodisperse oligo(ethyl glyoxylate) chains having light‐responsive end‐groups. These SIJDs self‐assemble into spherical nanoparticles in aqueous media. Upon ultraviolet (UV) light irradiation, the hydrophobic oligo(ethyl glyoxylate) units exhibit rapid end‐to‐end self‐immolation within minutes. The depolymerization at the molecular level leads to a degradation pathway from spherical to crescent‐shaped nanoparticles, which can be used for the rapid release of encapsulated molecules of interest.

## Graphical abstract

Self‐immolative Janus dendrimers assemble into spherical nanoparticles with solid cores. Upon irradiation, rapid end‐to‐end depolymerization triggers a unique degradation pathway from spheres to crescents within minutes, enabling on‐demand release of encapsulated cargo. This rapid photodegradation provides assemblies with predefined lifetimes and points toward dynamic supramolecular states for stimuli‐responsive applications.

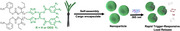

## 1 Introduction

In nature, amphiphilic molecules that self‐assemble with high precision underpin many critical biological processes [1, 2]. Notable examples include lipid bilayers of the cell membrane that compartmentalize cells from the extracellular environment, the self‐assembly of bile salts to facilitate lipid digestion [3], and protein assemblies that mediate the selective transport of ions [4]. The development of supramolecular chemistry enables researchers to design self‐assemblies with rich structures and properties for various applications, such as drug delivery [5, 6], imaging [7], information storage [8], and nanopatterning [9]. An essential feature of advanced self‐assemblies is on‐demand degradation to perform functions or to decompose at their end of use [10, 11]. This degradation is particularly important in biomedical scenarios [10, 12, 13] and has recently gained popularity in fields such as plastics recycling using biodegradable materials with controlled assembly and disassembly [14]. A well‐known strategy is the development of stimuli‐responsive self‐assemblies, which undergo changes in solubility or bond cleavages in response to stimuli such as light [15], pH [16], redox potential [17], and reactive oxygen species [18]. Traditional stimuli‐responsive systems require stoichiometric quantities of stimuli to fully degrade, resulting in incomplete and less controlled release kinetics in biological environments when stimuli are present at very low concentrations. This bottleneck was overcome by the introduction of self‐immolative polymers (SIPs) [19].

As a unique sub‐class of stimuli‐responsive polymers, certain SIPs can employ polymer backbones with ceiling temperatures below the operational temperature, favoring depolymerization at ambient temperature. The entropically disfavored polymerized state is trapped with a cleavable stimuli‐responsive end‐cap. Upon triggering and consequent removal of the end‐cap, the polymer chain undergoes depolymerization in a head‐to‐tail manner, reverting to its monomer state. This approach provides chemical amplification, as one single cleavage event is sufficient to fully depolymerize an entire polymer chain, greatly enhancing the polymer's biological signal sensitivity [20]. It also enables monomer recovery, offering the potential for easy recyclability [21]. Self‐immolative poly(benzyl carbamate) (PBC)‐poly(*N*,*N*‐dimethylacrylamide) block copolymers were designed by Liu and coworkers with light or thiol‐responsive end‐caps to trigger the release of payloads [22]. Vallet‐Regí and coworkers used PBC to fill the pores of mesoporous carbon nanoparticles to achieve payload release in acidic pH within lysosomal compartments within cells [23]. We have also previously reported poly(ethyl glyoxylate) (PEtG)‐based SIP assemblies equipped with pH‐ or UV‐responsive end‐caps [24, 25]. However, many of these systems suffer from relatively slow responses to stimuli, typically requiring long exposure periods to achieve complete end‐cap cleavage, followed by slow degradation kinetics ranging from hours to days to fully depolymerize [24, 25]. This slow response hampers their further potential in biomedical applications where a rapid release is demanded to ensure efficient delivery at target sites before being metabolized or migrating to an off‐target location [26]. In addition, conventional SIPs exhibit significant chain length dispersity and limited control over polymerization, resulting in potential batch‐to‐batch variability in both polymer properties and the resulting assemblies. Importantly, while cargo release has been demonstrated, previous studies provided little insight into the accompanying morphological transitions during depolymerization, which is key to understanding and controlling the behavior of supramolecular assemblies.

These challenges can potentially be addressed by assembling self‐immolative Janus dendrimers (SIJDs) to endow the assemblies with rapid responses to stimuli in the context of well‐defined monodisperse amphiphiles. Janus dendrimers are precisely defined molecules with a modular architecture that allows systematic variation of their hydrophilic and hydrophobic domains, combining structural control with synthetic flexibility [27, 28]. We recently reported the complex energy landscapes with pathway selections of self‐assemblies from amphiphilic JD molecules, which were composed of hydrophilic oligo(ethylene glycol) (OEG) and hydrophobic aliphatic chains on phenolic acid units connected by a pentaerythritol core [29, 30]. These findings demonstrated that the JD molecules were sufficiently dynamic to form diverse supramolecular self‐assembled structures. The intrinsic dynamic behavior, combined with the rich chemical design and precise molecular architecture, provides JD assemblies with substantial functional potential. Notably, ionizable amphiphilic Janus dendrimers have efficiently encapsulated and delivered mRNA, with their modular hydrophilic and hydrophobic domains enabling high transfection efficiency and organ‐specific targeting [31, 32, 33]. As a single‐component system, they simplify formulation by integrating the functions of poly(ethylene glycol) (PEG), ionizable lipids, phospholipids, and cholesterol into a single molecule. While these assemblies were pH‐responsive through ionization of their amino groups, we also reported photodegradable Janus dendrimers containing 2‐nitrobenzyl carbonate units in the backbone of the hydrophobic block [15]. These JDs assembled to form dendrimersomes that were degraded by light. However, this approach did not allow for chemical amplification—the translation of a single stimulus‐mediated event into a cascade of subsequent reactions. To our knowledge, self‐assembling Janus dendrimers containing self‐immolative components have not yet been reported.

For the present work, we hypothesized that incorporating self‐immolative linkages into Janus dendrimers could build on the advantages of JDs by introducing a built‐in mechanism for rapid and cascade‐driven molecular breakdown [21, 34]. This capability could provide precise temporal control over assembly disintegration and cargo release, thereby extending the utility of JD systems to applications requiring triggered, self‐regulated, or transient function. To test this hypothesis, we designed SIJD molecules by linking hydrophilic OEG‐functionalized phenolic acid dendrons with hydrophobic monodisperse self‐immolative oligo(ethyl glyoxylate) (OEtG). Upon UV irradiation to remove the end‐cap, the OEtG underwent degradation, triggering a rapid structural transition of the supramolecular self‐assemblies from spherical to crescent‐shaped nanoparticles. This rapid photodegradation response was demonstrated to be generally applicable to SIJDs with different OEG substitution patterns in the hydrophilic block and SIJDs with different OEtG lengths in the hydrophobic block. By encapsulating Nile red as a model cargo, we demonstrate that its rapid and controllable release upon the degradation of the nanoparticles highlights the potential of these photo‐responsive supramolecular assemblies for advanced stimuli‐responsive applications.

## 2 Results and Discussion

### 2.1 Synthesis of SIJD Molecules

**SCHEME 1:**
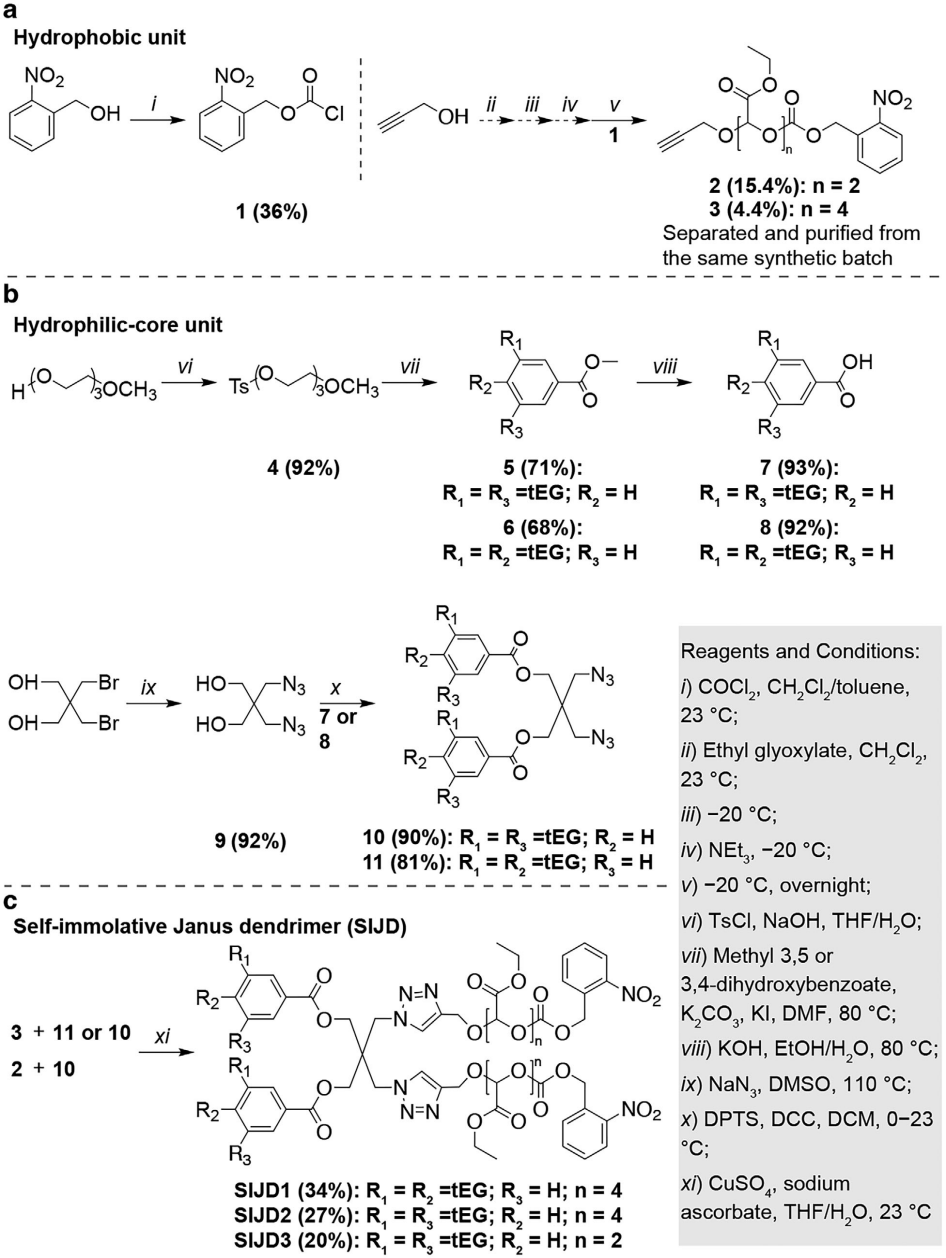
Synthesis of (a) the hydrophobic unit, (b) hydrophilic‐core unit, and (c) self‐immolative Janus dendrimer (SIJD) molecules.

**FIGURE 1:**
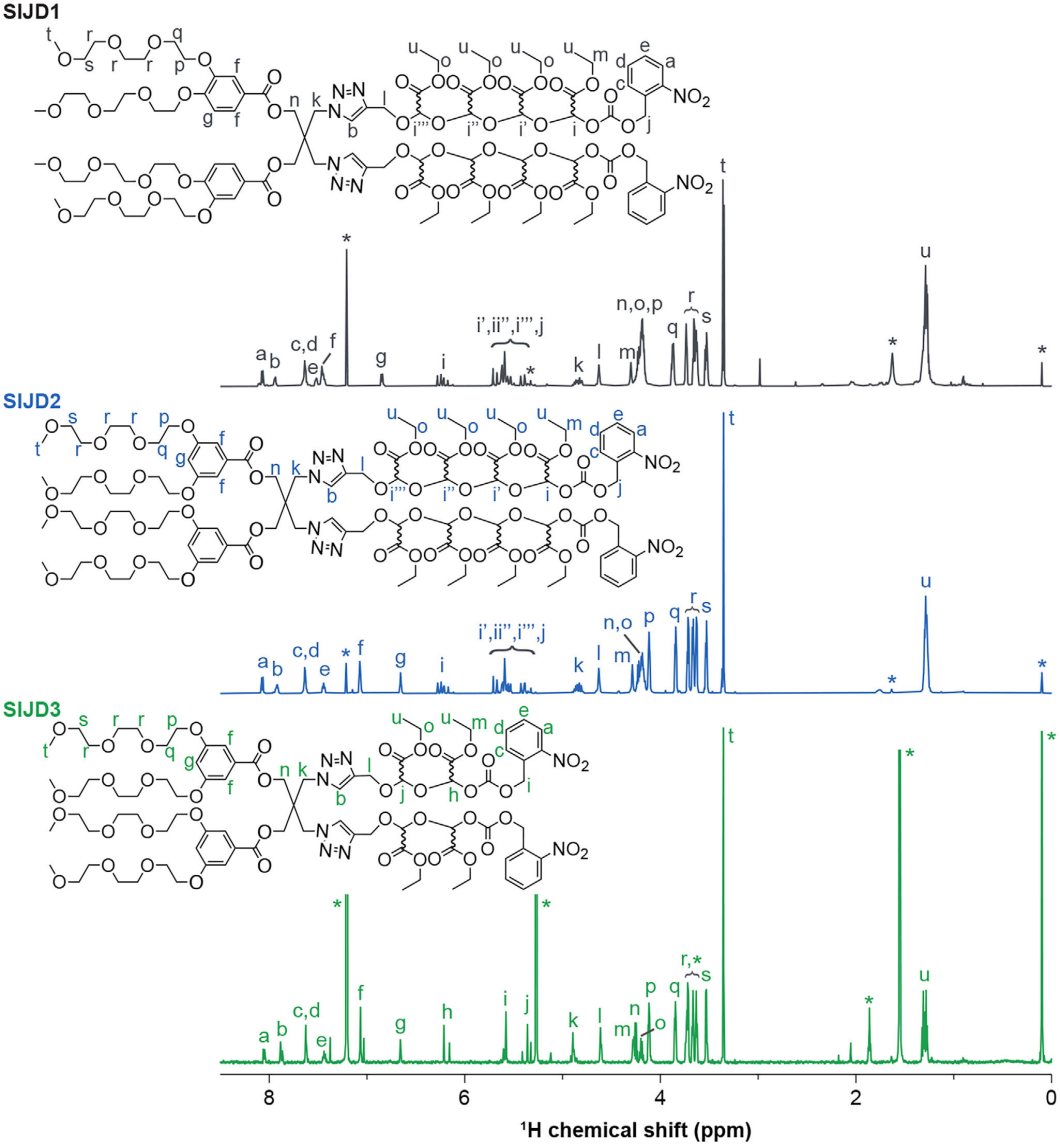
^1^H NMR spectra of three SIJD molecules (**SIJD1−3**) in CDCl_3_ (600 MHz). The multiple peaks observed for the ethyl glyoxylate oligomers reflect the presence of stereoisomers originating from the backbone acetal carbon, for which the stereochemistry is undefined and therefore indicated by a wavy bond. Asterisks denote solvent peaks, with detailed assignments provided in the Supporting Information.

The SIJD molecules were synthesized by a modular synthetic strategy (Scheme 1) [28]. First, the hydrophobic unit was synthesized by functionalization of OEtGs with alkynes (Scheme 1a). Oligomerization of freshly distilled ethyl glyoxylate monomer [35] was initiated with propargyl alcohol, and then the resulting oligomers were end‐capped with 2‐nitrobenzyl chloroformate (**1**) to introduce photo‐responsive end‐caps. Instead of using precipitation, which is the favored purification method for long‐chain PEtGs [21, 34, 35], the oligomers with different chains lengths were successfully separated and purified by silica gel column chromatography. The resulting oligomers with defined chain lengths allow the synthesis of monodisperse SIJD molecules. Efforts focused on the isolation of the dimer (**2**) and tetramer (**3**). These lengths of OEtG were selected to achieve comparable volume fractions of hydrophilic and hydrophobic domains within the resulting amphiphiles. Their successful syntheses were confirmed by ^1^H and ^13^C NMR spectroscopy, as well as mass spectrometry (Figures S2, S3, S15, S16, and S26). It is noteworthy that due to the asymmetric backbone acetal carbon, different stereoisomers of the oligomers were observed in the NMR spectra compared to previously synthesized PEtG where the backbone CH signals appeared as a broad peak from 5.5–6.0 ppm [34]. Next, the hydrophilic portions of the Janus dendrimers [28, 29, 30] (**7** and **8**) were conjugated to the pentaerythritol core functionalized with azides (**9**), resulting in hydrophilic‐core units (**10** and **11**, Scheme 1b). All products were carefully characterized by NMR spectroscopy and mass spectrometry (Figures S4–S11, S17–S22, and S27). Finally, a copper‐catalyzed azide‐alkyne cycloaddition reaction was performed, followed by chromatographic purification, to obtain three SIJD molecules with different substitution patterns (**SIJD1** and **SIJD2**) and OEtG lengths (**SIJD2** and **SIJD3**) (Scheme 1c). The monodispersity of the synthesized molecules was confirmed by ^1^H (Figure 1; Figures S12–S14) and ^13^C NMR spectroscopy (Figures S23–S25), mass spectrometry (Figures S28–S30), and high‐performance liquid chromatography (HPLC, Figure S31).

### 2.2 Self‐Assembly of SIJD Molecules

**FIGURE 2:**
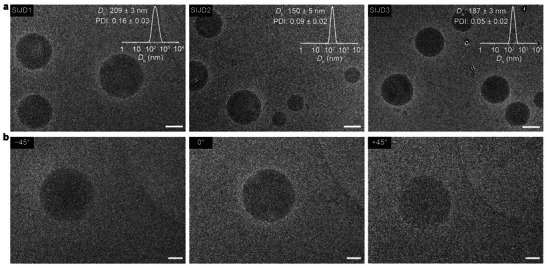
(a) Cryo‐TEM images of SIJD self‐assemblies. Insets: intensity‐weighted *D_h_* distribution of SIJD self‐assemblies with *D_h_* and PDI values as determined by DLS. The numerical data are presented as mean ± standard deviation (s.d.) from three different samples. (b) Cryo‐TEM images of **SIJD1** at different tilt angles demonstrating the spherical morphology of the nanoparticle. Scare bars are 100 nm.

Self‐assembly of the SIJDs was then investigated using a simple injection method. First, an ethanol/water solvent combination was used as we have previously reported [29, 30]. However, all SIJDs displayed poor solubility in ethanol (Figure S32), leading to inconsistent results. In contrast, the injection of tetrahydrofuran (THF) solutions of SIJDs into water provided reproducible results in terms of hydrodynamic diameters of the particles (*D*_h_), polydispersity index (PDI), and count rate based on dynamic light scattering (DLS). All further self‐assembly studies were performed using the THF/water system. The stability of the assemblies was initially assessed by DLS in the presence or absence of THF and via room temperature or 4°C dialysis. Without THF removal or when dialyzed at room temperature, the PDI gradually increased, and the distribution became bimodal, indicating progressive aggregation over time. In contrast, samples dialyzed at 4°C maintained consistent particle sizes and PDI over a one‐week post‐dialysis stability test at 4°C, while preserving a stable unimodal size distribution (Figure S33). The instability of the assemblies was attributed to the presence of 5% THF at room temperature, which likely fluidized the particles and facilitated interparticle aggregation at a relatively rapid rate. Dialysis at 4°C retained the rigidity of the assemblies during THF removal, resulting in stable suspensions in the aqueous environment. Further studies confirmed that after THF removal, the assemblies were also stable at room temperature for at least one week (Figure S33). The assemblies of **SIJD1**, **SIJD2**, and **SIJD3** showed *D*_h_ of 209 ± 3 nm, 150 ± 5 nm, and 187 ± 3 nm, with corresponding low PDI values of 0.16 ± 0.03, 0.09 ± 0.02, and 0.05 ± 0.02, respectively (insets in Figure 2; Table S1). These results indicate homogenous populations of nanoparticles in each case, which is crucial in further applications [36]. The relatively higher PDI for **SIJD1** suggests that the (3,4)‐OEG substitution pattern was less effective than the (3,5)‐OEG pattern in stabilizing the longer OEtG tetramer, leading to less uniform structures. The morphologies of self‐assemblies were investigated by cryogenic‐transmission electron microscopy (cryo‐TEM). Self‐assemblies of all three SIJDs showed structures with electron‐dense cores (Figure 2a), which are reminiscent of the lipid nanoparticles [37, 38, 39, 40]. Additional cryo‐TEM imaging at different tilt angles provided further evidence of the spherical morphology of the nanoparticles (Figure 2b). Although it is certain that the hydrophilic OEG units will be present at the surface [28, 29, 30], the mesoscopic core structure of the spherical nanoparticles could be amorphous [38, 39] or ordered inverse phases [37, 40], with further detailed investigations needed to confirm this aspect.

### 2.3 Photodegradation of SIJD Self‐Assemblies

To investigate the photodegradation of the nanoparticles, samples were irradiated with a 360–365 nm LED light (12 mW/cm^2^) for 32 min. An obvious transition was observed as the particle suspensions turned from a translucent state to a clear state (Figure S34), indicating efficient UV light penetration to trigger the photodegradation process. The resulting solution was light yellow, corresponding to the expected 2‐nitrosobenzaldehyde photolysis product [41].

NMR spectroscopy confirmed that light‐induced depolymerization occurred. The aqueous media of the assemblies were switched to D_2_O by centrifugation and resuspension, and then spectra were acquired before and after 15 min of UV exposure (Figure S35). Prior to irradiation, no peaks were observed due to the lack of molecular motion of the Janus dendrimers. Following irradiation, peaks corresponding to the depolymerization product ethyl glyoxylate hydrate were observed as this soluble product was released from the assemblies into the solution.

**FIGURE 3:**
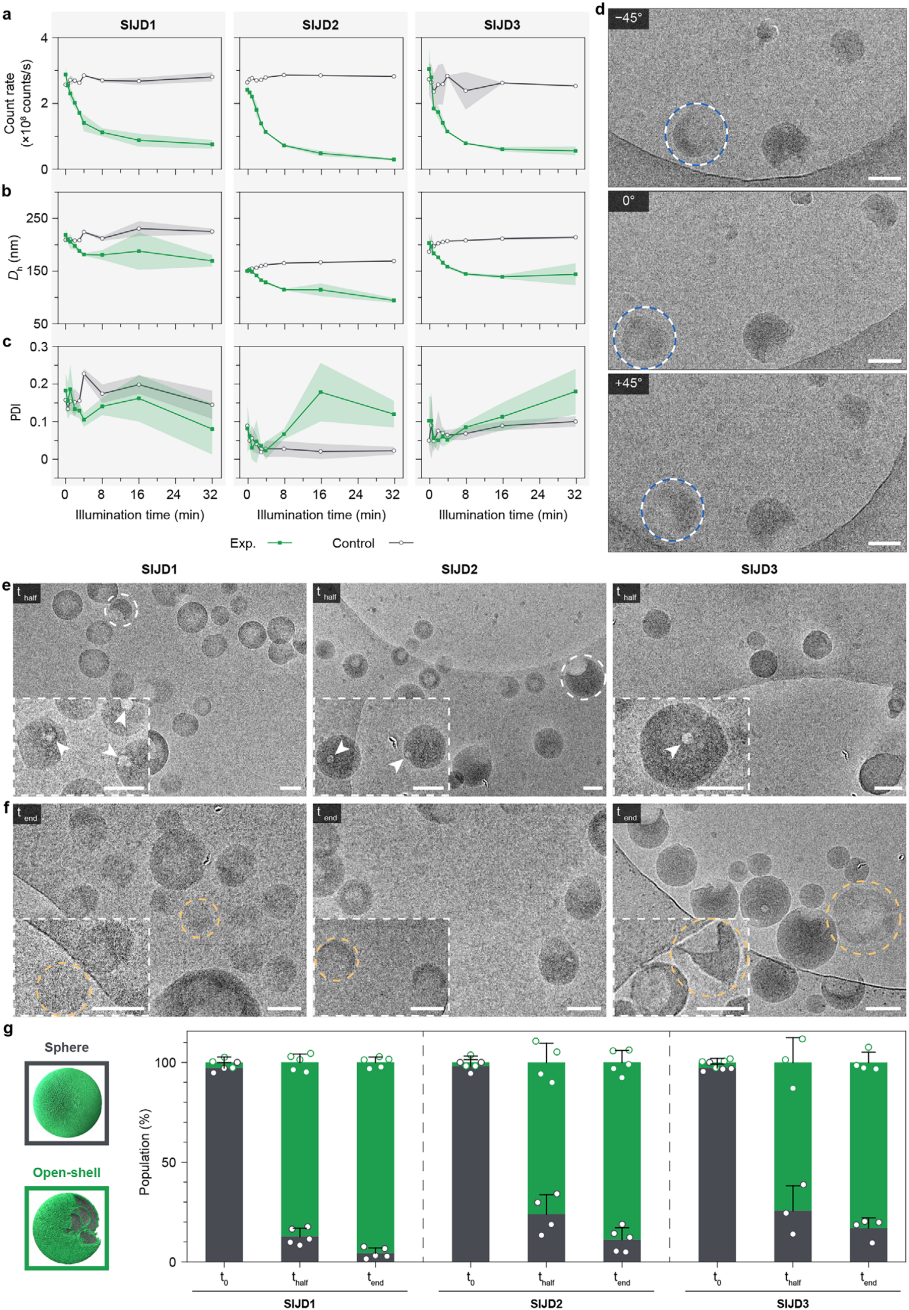
UV degradation of three SIJD self‐assemblies. (a) Derived count rate, (b) Z‐average diameters, and (c) PDI of the assemblies over time with UV light irradiation (Exp.) and kept in the dark (Control). Measurements were performed on triplicate samples, with the s.d. presented as the shaded areas. (d) Cryo‐TEM images of **SIJD1** after UV irradiation at different tilt angles demonstrating the crescent morphology of the nanoparticles. The opening of an apparent hollow particle at 0° could be observed from other angles, as highlighted by blue/white dashed circles. (e,f) Cryo‐TEM images of SIJD self‐assemblies at **t_half_** (e) and **t_end_** (f) following UV irradiation. Insets are zoomed‐in images of representative self‐assembly structures. The formation of pores within nanoparticles, typical crescent, and final structures are highlighted by arrows, white and yellow dashed circles, respectively. Scale bars are 100 nm. (g) Quantitative analysis of assembly populations before (**t_0_**) and after UV degradation (**t_half_** and **t_end_**). For each condition, images from multiple areas were analyzed to minimize the error (*n* > 100 particles for **SIJD1** (**t_0_**), **SIJD2** (**t_0_**), and **SIJD3** (**t_half_**); *n* > 200 particles for **SIJD2** (**t_half_**) and **SIJD3** (**t_0_**); *n* > 300 particles for other conditions. The data is presented as the mean ± s.d.

Next, DLS was used to quantitatively study the photodegradation process of the nanoparticles with the *D_h_*, PDI, and derived count rate monitored (Figure 3a–c; Table S1). Over 32 min of irradiation, the derived count rates decreased by 74%–88% compared to the initial value, with the majority of the decrease taking place in the first 8 min. At the same time, the *D_h_* decreased by 23%–29%, and the PDI increased significantly, except in the case of **SIJD1** (Figure S36). The anomalous trend for **SIJD1** might stem from its relatively higher initial PDI in comparison to **SIJD2** and **SIJD3**. Based on DLS data, the current SIJD system showed faster degradation kinetics compared to our previous work on polymeric PEtG based nanoparticles with the same UV irradiation setup, where after 30 min of UV irradiation, the polymer only partially depolymerized over 24 h [25]. All control samples kept in the dark did not undergo any notable changes in count rates, *D_h_*, or PDI. These results suggest that, following cleavage of the 2‐nitrobenzyl carbonate end‐cap, self‐immolative deoligomerization of the OEtG groups occurred, leading to rearrangement of the assemblies into particles with decreased size, a reduction of particle concentration, and/or the formation of less dense particles with lower light‐scattering intensity.

The structures of the assemblies during photodegradation were further examined using cryo‐TEM at **t_half_**, when the count rate had decreased to 50% of its initial value corresponding to the system's half‐life, and at **t_end_**, at the completion of UV irradiation after 32 min. These time points were selected to capture both the intermediate and terminal states of the photoinduced degradation process. Compared with the nanoparticles prior to light illumination (Figure 2), all assemblies displayed morphological transitions from spherical to crescent‐shaped nanoparticles at **t_half_** (highlighted by white dashed circles in Figure 3d,e), preceded by the formation of pores within nanoparticles (indicated by arrows in the insets of Figure 3e). These pores locally expose the internal structure to water and degradation intermediates, where molecular mobility and accessibility are relatively higher, promoting further material loss around the opening. As degradation progresses preferentially at these sites, the remaining shells relax into a crescent‐like morphologies. Some particles appeared hollow due to the viewing angle, with the opening discernible at alternative tilt angles (highlighted by blue/white dashed circles in Figure 3d). The missing structure observed in the crescent nanoparticles was attributed to the self‐immolative depolymerization of OEtG groups, which initiated the disruption of the nanoparticles. The degradation process progressed further by **t_end_** (Figure 3f), when the crescent particles exhibited extensive erosion and material loss, ultimately leading to their disappearance (highlighted by yellow dashed circles in Figure 3f). The loss of material from individual particles followed by the eventual disassembly of nanoparticles accounts for the reduction in the DLS count rate over time. To investigate the kinetics of the morphological transition, we quantified the populations of spherical and open‐shelled nanoparticles before (**t_0_**) and after UV illumination (**t_half_** and **t_end_**) by imaging a substantial number of particles across multiple regions (Figure 3g). All three SIJD assemblies showed a similar transition from predominantly spherical nanoparticles with electron‐dense cores at **t_0_** (>97%) to largely open‐shelled structures after photodegradation at **t_half_** and **t_end_** (>80%), yielding nanoparticles with pores and crescent morphologies.

### 2.4 Photo‐Triggered Cargo Release and In Vitro Cytotoxicity

**FIGURE 4:**
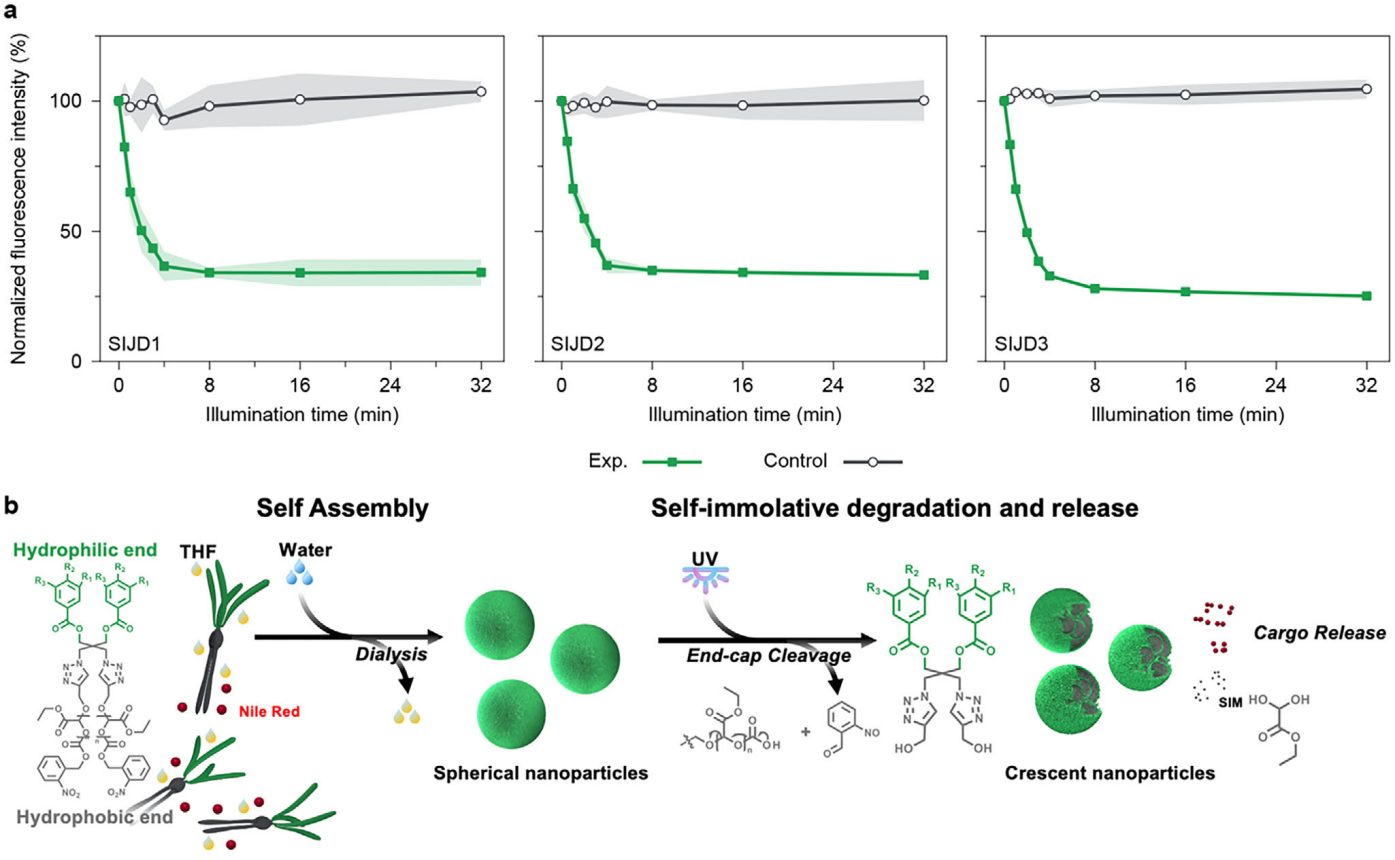
(a) Nile red release from three SIJD self‐assemblies under UV irradiation (Exp.) and dark conditions (Control), using the same exposure duration as in degradation studies. The results are presented as the mean of triplicate samples, with s.d. presented as the shaded areas. (b) Schematic representation of SIJD self‐assembly and subsequent UV‐triggered degradation and Nile red release. Upon UV irradiation, the end‐cap is cleaved, exposing OEtG chains which depolymerize head‐to‐tail to release Nile red cargo and monomer hydrate.

The potential for photodegradation‐induced cargo release was investigated. Nile red, a lipophilic dye, was selected as a model molecule due to its photostability under UV irradiation [42, 43, 44]. Furthermore, Nile red shows strong fluorescence in hydrophobic environments, while its fluorescence is quenched in aqueous media due to poor solubility and subsequent aggregation. This feature enables its release to be reliably and sensitively probed by fluorescence spectroscopy. To encapsulate Nile red, the dendrimers and Nile red were first co‐dissolved in THF, followed by the same self‐assembly procedure and THF removal by dialysis as described above. Nile red in aqueous solution (without assemblies) exhibited only about 1% of the fluorescence observed when it was encapsulated into the assemblies (Figure S37). To study the photo‐triggered release, the samples were irradiated with 365 nm light for 32 min, and the fluorescence emission intensity was measured at 620 nm periodically. While a negligible amount of Nile red was released in the absence of light trigger, all three SIJD assemblies exhibited consistent UV‐triggered release, as indicated by a reduction in fluorescence intensity to 25%–35% of the initial level (Figure 4a; Figures S38–S40). Thus, the results indicate that approximately 65%–75% of encapsulated Nile red was discharged upon irradiation and the consequent loss of substantial portions of the hydrophobic domains that initially encapsulated the dye (Figure 4b). The residual 25%–35% fluorescence can be attributed to Nile red that was retained in the residual hydrophobic domains of the crescent nanoparticles that remained even after most of the dye has been discharged.

**FIGURE 5:**
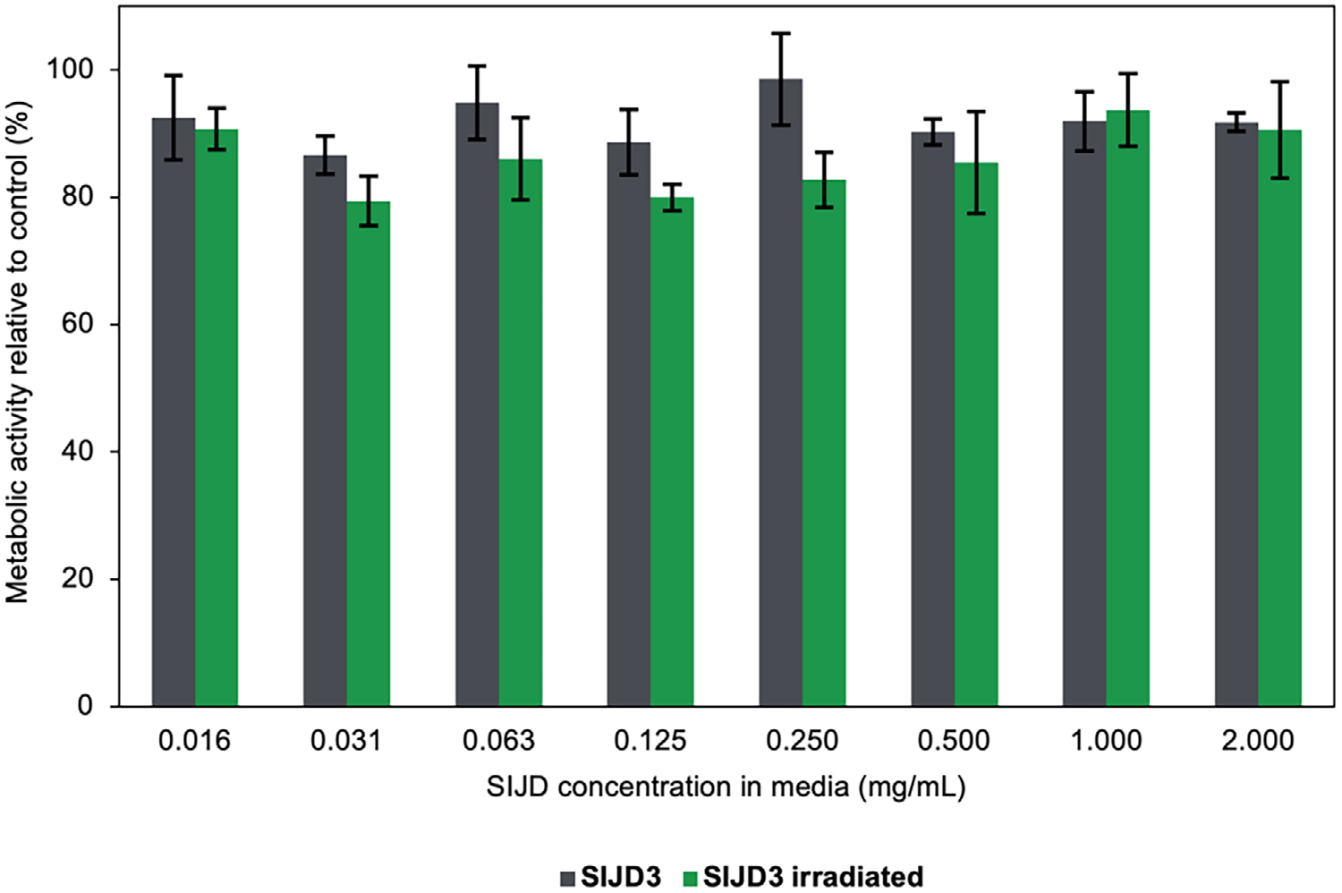
Cell metabolic activity measured by MTT assay after 24 h incubation of HeLa cells with varying concentrations of **SIJD3** assemblies without and after UV irradiation. The metabolic activity of cells exposed only to culture medium was defined as 100%. The data are presented as the mean ± s.d. of six replicates for each concentration.

Finally, to assess the potential of the SIJD assemblies for triggered release in therapeutic delivery applications, we evaluated their cytotoxicity to HeLa cells after 24 h of incubation using a 3‐(4,5‐dimethyl‐2‐thiazolyl)‐2,5‐diphenyl‐2H‐tetrazolium bromide (MTT) assay. **SIJD3** was selected because it contains the highest proportion of the 2‐nitrobenzyl carbonate end‐cap, the moiety most likely to cause adverse effects, while OEG‐based dendrons and PEtG have previously been found to be non‐toxic [45, 46]. Even at the highest tested concentration of 2.0 mg/mL, both non‐irradiated and UV‐irradiated samples resulted in metabolic activities at least 80% those of control, untreated cells, indicating their low cytotoxicities under these conditions (Figure 5).

## 3 Conclusions

In conclusion, monodisperse SIJDs with UV‐responsive end‐caps were successfully synthesized. Three types of SIJDs, varying in hydrophilic OEG substitution patterns and different hydrophobic OEtG lengths, were capable of self‐assembling into unimodal spherical nanoparticles with solid cores. Upon UV irradiation, cleavage of the 2‐nitrobenzyl end‐caps triggered rapid end‐to‐end depolymerization of OEtG chains within minutes, leading to a degradation pathway from spherical to crescent‐shaped nanoparticles. This rapid photodegradation response endowed the SIJD assembles with a predefined lifetime and precise, on‐demand disassembly, thereby enabling controlled release of encapsulated cargo molecules. We further envision that the final degradation molecules of glyoxylic acid hydrate, a metabolic intermediate, could engage in non‐covalent interactions to navigate the assemblies into diverse supramolecular states. This potential dynamic behavior points to a rich energy landscape [29, 30] of SIJD assemblies, offering great potential in stimuli‐responsive applications and the design of complex out‐of‐equilibrium supramolecular systems. In future work toward advanced biomedical applications, the modular architecture of SIJDs will allow the UV light‐responsive 2‐nitrobenzyl end‐caps to be readily replaced with alternative end‐caps, such as boronates or disulfide groups that respond to reactive oxygen species or reducing thiols, respectively. These species are intrinsically present in vivo and are often elevated in pathological conditions such as inflammation or cancer.

## 4 Experimental Section

### 4.1 Synthesis of Self‐Immolative Janus Dendrimers (SIJDs): SIJD1, SIJD2, and SIJD3

All Three Dendrimers Were Synthesized Using the Same General Procedure as **SIJD3**. A solution of CuSO_4_ (1.80 mg, 0.0150 mmol, 0.600 equiv) in 0.05 mL of H_2_O, a solution of compound **2** (24.9 mg, 0.0460 mmol, 2.40 equiv) in THF/H_2_O (0.150/0.050 mL) and a solution of compound **10** (20.0 mg, 0.0190 mmol, 1.00 equiv) in THF/H_2_O (0.150/0.050 mL) were added to a 1 mL glass vial equipped with a magnetic stir bar and purged with N_2_ for 2 min. Then, a solution of sodium ascorbate (2.30 mg, 0.0150 mmol, 0.600 equiv) in 0.05 mL of H_2_O was added. The reaction mixture was stirred at room temperature for 48 h and the solvents were removed under high vacuum. The product was purified by silica gel column chromatography using a gradient eluent system (1 v/v% to 3 v/v% methanol in CH_2_Cl_2_) to give **SIJD 3** as a tacky solid. Characterization data are included in the Supporting Information.

### 4.2 Self‐Assembly

All Three SIJDs Were Self‐Assembled Using the Same Procedure. Into a 0.5 mL Eppendorf tube, 1.2 mg of the sample was added. The dendrimer was then dissolved in 120 µL of THF with vortex mixing. Deionized water (2 mL) was added to a 1‐dram glass vial. A 100 µL aliquot of the dendrimer solution in THF was drawn using a micropipette and injected directly into water with the pipette tip submerged beneath the surface. The resulting suspension was then subjected to 5 s of vortex mixing. Then, the suspension was transferred to a dialysis tube and dialyzed against 500 mL of deionized water in a beaker submerged in a 4°C bath for 4 h to remove THF. The sample was then stored at 4°C.

DLS measurements were carried out on Malvern Zetasizer Nano‐ZS (Malvern Instruments) equipped with a He‐Ne laser (633 nm, 4 mW) and an Avalanche photodiode detector (173°) to evaluate the *Z*‐average diameter, derived count rate, and polydispersity (PDI) of the assemblies. The samples were prepared at 0.5 mg/mL in polystyrene cuvettes. TEM was used to characterize the shape of the nanoparticles.

### 4.3 General Procedure for UV Degradation of Self‐Assembled SIJD1, SIJD2, and SIJD3

All Three SIJD Assemblies Were Degraded Using the Same Procedure. Triplicates of each Janus dendrimer assembly were placed in a UV box equipped with LEDs (365–370 nm, intensity: 10 mW/cm^2^) and irradiated for 32 min. DLS data were acquired at 0, 0.5, 1, 2, 3, 4, 8, 16, and 32 min. The attenuator index value was set to automatic mode to run all initial scans (i.e., t = 0 min). After the initial scans, the subsequent scans were then manually fixed to be the same attenuator index value throughout the experiments. Since all initial experiments had the attenuator automatically optimized to 5, all subsequent experiments were also set to 5, meaning 0.1% transmission (% nominal) of the light source passes the attenuator as per Malvern Technical Note states. Fresh samples were used for the NMR/UV degradation study for three nanoparticles apart from the DLS/UV degradation study. To replace the medium with D_2_O, 0.5 mL of each self‐assembled solution was taken to the ultracentrifuge at 12 344 rpm (14 000 g) for 15 min using an Optima XPN Ultracentrifuge with a Type 70 fixed‐angle titanium rotor. After decanting the supernatant, the resulting white pallet was resuspended in D_2_O and transferred to NMR tubes for irradiation. Aliquots of the sample solution at **t_half_** and **t_end_** were collected and concentrated via centrifugation, then imaged by cryo‐TEM.

### 4.4 Encapsulation and Release of Nile Red From Self‐Assembled Nanoparticles

To 1.2 mg of either **SIJD1**, **SIJD2,** or **SIJD3**, 120 µL of a 2.5 mg/mL Nile red solution in THF (2% w/w, Nile red/SIJD) was added. This solution was mixed using a vortex mixer for 5 min in the dark to ensure complete dissolution. Subsequently, the same self‐assembly injection and dialysis procedures were applied as described previously.

For the light‐triggered release study, 2 mL of the Nile red‐encapsulated assembly suspension was diluted with 4 mL of deionized water. The resulting sample was divided into 6 equal aliquots, each transferred into a quartz cuvette. Three cuvettes were irradiated with UV light for 32 min, while the remaining three were kept in the dark as controls. Fluorescence emission spectra were recorded at designated time points using an excitation wavelength of 540 nm and emission range of 550–750 nm. The fluorescence intensity at the maximum emission wavelength (620 nm) was used to calculate the percentage of initial fluorescence over time.

### 4.5 Cryo‐TEM Imaging

Cryo‐TEM imaging was performed using a JEOL 2100 transmission electron microscope operating at 200 kV and equipped with a high‐resolution Gatan 895 Ultrascan 4000 bottom‐mount camera (4080 × 4080 pixels) to capture the morphologies of the self‐assembled structures. Quantifoil TEM grids were glow‐discharged using a 208 carbon coater (Cressington) prior to sample loading. To ensure adequate particle concentration, samples were first concentrated by centrifugation. A 3.5 µL aliquot of the concentrated solution was then applied to the grid, blotted, and vitrified by rapid plunging into liquid ethane under 100% humidity using an FEI Vitrobot Mark IV (blot time: 1.5 s, blot force: 2). Vitrified grids were loaded into a Gatan 914 high‐tilt cryoholder (Munich, Germany) for imaging. Image analysis was conducted using Fiji ImageJ software (v2.1.0).

### 4.6 Statistical Analysis

No pre‐processing of the data was performed. The results are presented as the mean ± s.d. The number of replicates is indicated in the figure caption for each data set. No formal statistical tests were performed.

## Author Contributions

J.L. conceived the concept. J.L. and C.L. designed and performed the experiments, analyzed the results, and prepared the manuscript. D.A.W. and E.R.G. supervised the research. All authors contributed to the analysis of the results and commented on the manuscript.

## Conflicts of Interest

The authors declare no conflicts of interest.

## Supporting information




**Supporting File**: smll72720‐sup‐0001‐SuppMat.pdf.

## Data Availability

The data that support the findings of this study are available from the corresponding author upon reasonable request.

## References

[1] G. Bao and S. Suresh , “Cell and Molecular Mechanics of Biological Materials,” Nature Materials 2 (2003): 715–725. 14593396 10.1038/nmat1001

[2] A. Singh , P. Parvin , B. Saha , and D. Das , “Non‐Equilibrium Self‐Assembly for Living Matter‐Like Properties,” Nature Reviews Chemistry 8 (2024): 723–740. 39179623 10.1038/s41570-024-00640-z

[3] P. Y. Parekh , V. I. Patel , M. R. Khimani , and P. Bahadur , “Self‐Assembly of Bile Salts and Their Mixed Aggregates as Building Blocks for Smart Aggregates,” Advances in Colloid and Interface Science 312 (2023): 102846. 36736167 10.1016/j.cis.2023.102846

[4] L. Tu and C. Deutsch , “Evidence for Dimerization of Dimers in K^+^ Channel Assembly,” Biophysical Journal 76 (1999): 2004–2017. 10096897 10.1016/S0006-3495(99)77358-3 PMC1300175

[5] J. Qian , Y. Guo , Y. Xu , X. Wang , J. Chen , and X. Wu , “Combination of Micelles and Liposomes as a Promising Drug Delivery System: A Review,” Drug Delivery and Translational Research 13 (2023): 2767–2789. 37278964 10.1007/s13346-023-01368-x

[6] W. Shao , K. Miao , H. Liu , C. Ye , J. Du , and Y. Zhao , “Acid and Reduction Dually Cleavable Amphiphilic Comb‐Like Copolymer Micelles for Controlled Drug Delivery,” Polymer Chemistry 4 (2013): 3398.

[7] W. Tan , Q. Zhang , P. Hong , and B. Xu , “A Self‐Assembling Probe for Imaging the States of Golgi Apparatus in Live Single Cells,” Bioconjugate Chemistry 33 (2022): 1983–1988. 35312281 10.1021/acs.bioconjchem.2c00084 PMC9489815

[8] Y. Xie , Z. Tong , T. Xia , et al., “2D Hierarchical Microbarcodes With Expanded Storage Capacity for Optical Multiplex and Information Encryption,” Advanced Materials 36 (2024): 2308154. 10.1002/adma.202308154 38014933

[9] C. Tang , E. M. Lennon , G. H. Fredrickson , E. J. Kramer , and C. J. Hawker , “Evolution of Block Copolymer Lithography to Highly Ordered Square Arrays,” Science 322 (2008): 429–432. 18818367 10.1126/science.1162950

[10] C. Sharma and A. Walther , “Self‐Regulating Colloidal Co‐Assemblies That Accelerate Their Own Destruction via Chemo‐Structural Feedback,” Angewandte Chemie International Edition 61 (2022): 202201573. 10.1002/anie.202201573 PMC9311650 35235231

[11] Z. Tong , Y. Xie , M. C. Arno , et al., “Uniform Segmented Platelet Micelles With Compositionally Distinct and Selectively Degradable Cores,” Nature Chemistry 15 (2023): 824–831. 10.1038/s41557-023-01177-2 PMC10239731 37081206

[12] H. Liu , H.‐H. Lu , Y. Alp , R. Wu , and S. Thayumanavan , “Structural Determinants of Stimuli‐Responsiveness in Amphiphilic Macromolecular Nano‐Assemblies,” Progress in Polymer Science 148 (2024): 101765. 38476148 10.1016/j.progpolymsci.2023.101765 PMC10927256

[13] P. Wei , N. Gu , Q. Gu , J. Jiang , J. Chen , and J. Du , “Preparation and Mechanism Insight of Biodegradable Kippah Vesicles,” Small 21 (2025): 2501838. 10.1002/smll.202501838 40264332

[14] A. Niazov‐Elkan , H. Weissman , E. Shimoni , et al., “Emergent Self‐Assembly of Sustainable Plastics Based on Amino Acid Nanocrystals,” ACS Nano 17 (2023): 20962–20967. 37871004 10.1021/acsnano.3c02528 PMC10655173

[15] A. Nazemi and E. R. Gillies , “Dendrimersomes With Photodegradable Membranes for Triggered Release of Hydrophilic and Hydrophobic Cargo,” Chemical Communications 50 (2014): 11122–11125. 25110987 10.1039/c4cc05161k

[16] H. Shen , E. M. Lynch , S. Akkineni , et al., “De Novo Design of pH‐Responsive Self‐Assembling Helical Protein Filaments,” Nature Nanotechnology 19 (2024): 1016–1021. 10.1038/s41565-024-01641-1 PMC11286511 38570702

[17] D. Li , R. Zhang , G. Liu , Y. Kang , and J. Wu , “Redox‐Responsive Self‐Assembled Nanoparticles for Cancer Therapy,” Advanced Healthcare Materials 9 (2020): 2000605. 10.1002/adhm.202000605 32893506

[18] B. Hu , Z. Lian , Z. Zhou , L. Shi , and Z. Yu , “Reactive Oxygen Species‐Responsive Adaptable Self‐Assembly of Peptides Toward Advanced Biomaterials,” ACS Applied Bio Materials 3 (2020): 5529–5551. 10.1021/acsabm.0c00758 35021788

[19] A. Sagi , R. Weinstain , N. Karton , and D. Shabat , “Self‐Immolative Polymers,” Journal of the American Chemical Society 130 (2008): 5434–5435. 18376834 10.1021/ja801065d

[20] A. D. Wong , M. A. DeWit , and E. R. Gillies , “Amplified Release Through the Stimulus Triggered Degradation of Self‐Immolative Oligomers, Dendrimers, and Linear Polymers,” Advanced Drug Delivery Reviews 64 (2012): 1031–1045. 21996055 10.1016/j.addr.2011.09.012

[21] B. Fan , J. F. Trant , R. E. Yardley , A. J. Pickering , F. Lagugné‐Labarthet , and E. R. Gillies , “Photocontrolled Degradation of Stimuli‐Responsive Poly(ethyl glyoxylate): Differentiating Features and Traceless Ambient Depolymerization,” Macromolecules 49 (2016): 7196–7203.

[22] G. Liu , X. Wang , J. Hu , G. Zhang , and S. Liu , “Self‐Immolative Polymersomes for High‐Efficiency Triggered Release and Programmed Enzymatic Reactions,” Journal of the American Chemical Society 136 (2014): 7492–7497. 24786176 10.1021/ja5030832

[23] M. Gisbert‐Garzarán , J. C. Berkmann , D. Giasafaki , et al., “Engineered pH‐Responsive Mesoporous Carbon Nanoparticles for Drug Delivery,” ACS Applied Materials & Interfaces 12 (2020): 14946–14957. 32141284 10.1021/acsami.0c01786 PMC7116326

[24] Z. Deng , X. Liang , and E. R. Gillies , “Click to Self‐Immolation: A “Click” Functionalization Strategy Towards Triggerable Self‐Immolative Homopolymers and Block Copolymers,” Angewandte Chemie International Edition 63 (2024): 202317063. 10.1002/anie.202317063 38029347

[25] X. Liang and E. R. Gillies , “Self‐Immolative Amphiphilic Diblock Copolymers With Individually Triggerable Blocks,” ACS Polymers Au 2 (2022): 313–323. 36254315 10.1021/acspolymersau.2c00013 PMC9562457

[26] X. Wang , C. Li , Y. Wang , et al., “Smart Drug Delivery Systems for Precise Cancer Therapy,” Acta Pharmaceutica Sinica B 12 (2022): 4098–4121. 36386470 10.1016/j.apsb.2022.08.013 PMC9643298

[27] A.‐M. Caminade , R. Laurent , B. Delavaux‐Nicot , and J.‐P. Majoral , “‘Janus’ Dendrimers: Syntheses and Properties,” New Journal of Chemistry 36 (2012): 217–226.

[28] V. Percec , D. A. Wilson , P. Leowanawat , et al., “Self‐Assembly of Janus Dendrimers Into Uniform Dendrimersomes and Other Complex Architectures,” Science 328 (2010): 1009–1014. 20489021 10.1126/science.1185547

[29] J. Luan , D. Wang , S. Zhang , Y. Miyazaki , W. Shinoda , and D. A. Wilson , “Complex Energy Landscapes of Self‐Assembled Vesicles,” Journal of the American Chemical Society 145 (2023): 15496–15506. 37427769 10.1021/jacs.3c04285 PMC10360149

[30] J. Luan , D. Wang , N. P. Kok , N. Moslehi , I. K. Voets , and D. A. Wilson , “Dynamic Pathways in Energy Landscapes Guiding Supramolecular Janus Dendrimer Self‐Assemblies Between Lamellar and Cubic Architectures,” Nature Communications 16 (2025): 8075. 10.1038/s41467-025-62866-9 PMC12397278 40883274

[31] D. Zhang , E. N. Atochina‐Vasserman , D. S. Maurya , et al., “One‐Component Multifunctional Sequence‐Defined Ionizable Amphiphilic Janus Dendrimer Delivery Systems for mRNA,” Journal of the American Chemical Society 143 (2021): 12315–12327. 34324336 10.1021/jacs.1c05813

[32] D. Sahoo , E. N. Atochina‐Vasserman , D. S. Maurya , et al., “The Constitutional Isomerism of One‐Component Ionizable Amphiphilic Janus Dendrimers Orchestrates the Total and Targeted Activities of mRNA Delivery,” Journal of the American Chemical Society 146 (2024): 3627–3634. 38306714 10.1021/jacs.3c13569

[33] M. Arshad , E. N. Atochina‐Vasserman , D. S. Maurya , et al., “Harnessing the Electron‐Withdrawing Inductive Effect of One‐Component Ionizable Amphiphilic Janus Dendrimers Unveils Cation−π Interactions and Their Important Roles to Targeted mRNA Delivery,” Journal of the American Chemical Society 147 (2025): 21347–21356. 40513049 10.1021/jacs.5c07232

[34] B. Fan , J. F. Trant , A. D. Wong , and E. R. Gillies , “Polyglyoxylates: A Versatile Class of Triggerable Self‐Immolative Polymers From Readily Accessible Monomers,” Journal of the American Chemical Society 136 (2014): 10116–10123. 24956012 10.1021/ja504727u

[35] A. Rabiee Kenaree and E. R. Gillies , “Controlled Polymerization of Ethyl Glyoxylate Using Alkyllithium and Alkoxide Initiators,” Macromolecules 51 (2018): 5501–5510.

[36] M. Danaei , M. Dehghankhold , S. Ataei , et al., “Impact of Particle Size and Polydispersity Index on the Clinical Applications of Lipidic Nanocarrier Systems,” Pharmaceutics 10 (2018): 57. 29783687 10.3390/pharmaceutics10020057 PMC6027495

[37] M. Yanez Arteta , T. Kjellman , S. Bartesaghi , et al., “Successful Reprogramming of Cellular Protein Production Through mRNA Delivered by Functionalized Lipid Nanoparticles,” Proceedings of the National Academy of Sciences 115 (2018): E3351–E3360. 10.1073/pnas.1720542115 PMC5899464 29588418

[38] J. A. Kulkarni , M. M. Darjuan , J. E. Mercer , et al., “On the Formation and Morphology of Lipid Nanoparticles Containing Ionizable Cationic Lipids and siRNA,” ACS Nano 12 (2018): 4787–4795. 29614232 10.1021/acsnano.8b01516

[39] T. Unruh , K. Götz , C. Vogel , et al., “Mesoscopic Structure of Lipid Nanoparticle Formulations for mRNA Drug Delivery: Comirnaty and Drug‐Free Dispersions,” ACS Nano 18 (2024): 9746–9764. 38514237 10.1021/acsnano.4c02610

[40] H. Yu , A. Angelova , B. Angelov , et al., “Real‐Time pH‐Dependent Self‐Assembly of Ionisable Lipids From COVID‐19 Vaccines and In Situ Nucleic Acid Complexation,” Angewandte Chemie International Edition 62 (2023): 202304977. 10.1002/anie.202304977 37391876

[41] Y. V. Il'ichev , M. A. Schwörer , and J. Wirz , “Photochemical Reaction Mechanisms of 2‐Nitrobenzyl Compounds: Methyl Ethers and Caged ATP,” Journal of the American Chemical Society 126 (2004): 4581–4595. 15070376 10.1021/ja039071z

[42] D. Han , X. Tong , and Y. Zhao , “Fast Photodegradable Block Copolymer Micelles for Burst Release,” Macromolecules 44 (2011): 437–439.

[43] J. Xuan , D. Han , H. Xia , and Y. Zhao , “Dual‐Stimuli‐Responsive Micelle of an ABC Triblock Copolymer Bearing a Redox‐Cleavable Unit and a Photocleavable Unit at Two Block Junctions,” Langmuir 30 (2014): 410–417. 24328893 10.1021/la404493n

[44] J. Dong , Y. Wang , J. Zhang , et al., “Multiple Stimuli‐Responsive Polymeric Micelles for Controlled Release,” Soft Matter 9 (2013): 370–373.

[45] P. Zhu , Y. Li , and D. Zhang , “One Component Ionizable Amphiphilic Janus Dendrimers for Targeted mRNA Delivery,” Angewandte Chemie International Edition 64 (2025): 202505304. 10.1002/anie.202505304 40192525

[46] B. Belloncle , C. Bunel , L. Menu‐Bouaouiche , O. Lesouhaitier , and F. Burel , “Study of the Degradation of Poly(ethyl glyoxylate): Biodegradation, Toxicity and Ecotoxicity Assays,” Journal of Polymers and the Environment 20 (2012): 726–731.

