# Supplementary material for: Supramolecular Assemblies of Self‐Immolative Janus Dendrimers With Rapid Photodegradation Response
Source: Small. 2026 Feb 5;22(19):e11067. doi: 10.1002/smll.202511067 (PMC13040118; doi:10.1002/smll.202511067)
Supplement: Supplementary file 1 — Supporting File: smll72720‐sup‐0001‐SuppMat.pdf. [file SMLL-22-e11067-s001.pdf]

Supporting Information

Supramolecular Assemblies of Self-Immolative Janus Dendrimers with Rapid Photodegradation Response

Chuanfeng Li[a,1], Jiabin Luan[c,1]*, Daniela A. Wilson[c]*, and Elizabeth R. Gillies[a,b]*

[a] Department of Chemistry and Centre for Advanced Materials and Biomaterials Research, The University of Western Ontario, London, Canada, N6A 5B7

[b] Department of Chemical and Biochemical Engineering and School of Biomedical Engineering, The University of Western Ontario, London, Canada, N6A 5B7

[c] Institute for Molecules and Materials, Radboud University, Nijmegen, The Netherlands

[1] These authors contributed equally: Chuanfeng Li, Jiabin Luan

*Corresponding author:

J.L.: j.luan@science.ru.nl; D.A.W.: d.wilson@science.ru.nl; E.R.G.: egillie@uwo.ca

Table of Contents

General Materials 3
General Methods 3
Cytotoxicity Assay 4
Synthesis 4
DLS UV Degradation Data 9
^{1}H NMR Spectra 10
^{13}C NMR Spectra 24
Mass Spectrometry 35
HPLC Data 40
Characterization of SIJD Self-assemblies 41
Fluorescence Spectra for Nile Red Release 45
References 49

General Materials

Ethyl glyoxylate in toluene solution (50% w/w), propargyl alcohol, and anhydrous copper (II) sulfate ($CuSO_4$) were obtained from Alfa Aesar (Haverhill, Massachusetts, USA). 2-Nitrobenzyl alcohol and tetrahydrofuran (THF) were obtained from AK Scientific (Union City, California, USA). Phosgene, sodium ascorbate, calcium hydride (CaH_2), phosphorus pentoxide (P_2O_5), dichloromethane (CH_2Cl_2) and triethylamine (NEt_3) were obtained from Millipore Sigma (Saint Louis, MO, United States). CH_2Cl_2 and NEt_3 were distilled over CaH_2 under a nitrogen atmosphere before use. Ethyl glyoxylate was purified over P_2O_5 as previously reported.[1] Tri(ethylene glycol) monomethyl ether, *p*-toluenesulfonyl chloride, methyl 3,5-dihydroxybenzoate, anhydrous dimethyl sulfoxide (DMSO), 4(dimethylamino)pyridine (DMAP), *p*-toluenesulfonic acid monohydrate (PTSA), *N,N'*-dicyclohexylcarbodiimide (DCC), were products from Sigma-Aldrich. Dimethylformamide (DMF), sodium hydroxide (NaOH), and sodium carbonate (Na_2CO_3) were purchased from Thermo Fisher Scientific. Absolute ethanol (EtOH), sodium sulfate (Na_2SO_4), and magnesium sulfate ($MgSO_4$) were products from VWR International. Potassium iodide (KI) was purchased from Merck. Potassium hydroxide (KOH) was bought from J.T.Baker, Avantor. 2,2-bis(bromomethyl)-1,3-propanediol and sodium azide (NaN_3) were bought from TCI Europe NV. Spectra/Por 6 dialysis membrane (pre-wetted regenerated cellulose tubing) with a molecular weight cut-off of 2 kD was obtained from Spectrum Labs (San Francisco, CA, United States). Deionized water was obtained from Barnstead EASYpure II ultrapure water system. All other chemicals were reagent grade and were used without further purification.

General Methods

Column chromatography was performed using SiliaFlash P60 silica gel (0.040−0.063 mm particle size, 230−400 mesh) from silicycle (Quebec City, QC, Canada). Thin layer chromatography (TLC) was carried out using Siliaplate silica gel F254 plates (20 cm × 20 cm, 250 μm).

NMR spectroscopy was conducted using a Bruker AvIII HD 400 MHz or 600 MHz Spectrometer. The ^1H and ^{13}C chemical shifts (δ) are reported in parts per million (ppm) relative to tetramethylsilane (TMS) and were calibrated against $CHCl_3$ (7.26 ppm) and $CDCl_3$ (77 ppm) respectively. Coupling constants (*J*) are expressed in Hertz (Hz).

High-resolution mass spectrometry (HRMS) was conducted on a Synapt high-definition mass spectrometer using electrospray (ESI) ionization.

HPLC was performed using a Waters Separations Module 2695, a photodiode array (PDA) detector (Waters 2998) with detection at 325 nm, and a Phenomenex Luna C8 3 μm (4.6 x 150 mm) column connected to a C8 guard column. Analyte separation was obtained using HPLC grade acetonitrile as the eluent.

Fluorescence data were obtained on a QM-4 SE spectrofluorometer equipped with double excitation and emission monochromators from Photon Technologies International.

DLS measurements were carried out on Malvern Zetasizer Nano-ZS (Malvern Instruments) equipped with a He-Ne laser (633 nm, 4 mW) and Avalanche photodiode detector (173°) to evaluate the *Z*-average diameter, derived count rate, and polydispersity (PDI) of the assemblies. The samples were prepared at 0.5 mg/mL in polystyrene cuvettes. TEM was used to characterize the shape of the nanoparticles.

Cytotoxicity Assay

SIJD3 was first self-assembled to obtain a suspension concentration of 20 mg/mL using the same procedure described in the manuscript. The sample was then split into two equal aliquots. One was kept in dark while the other was irradiated under UV light for 30 min using the same setup described in the manuscript. The two samples where then used in the cytotoxicity assays. HeLa cells (CCL-2, ATCC, USA) were cultured in Dulbecco's Modified Eagle Medium (DMEM) containing 10% fetal bovine serum (FBS), 1% Glutamax (100×) solution, and antibiotics (100 units/mL of each of Streptomycin and Penicillin). Cells were then seeded in a 96-well plate (~10,000 cells/well in 100 μL of culture media) and incubated for 24 h in an atmosphere containing 5% CO_2 at 37 °C. Meanwhile, the two samples were each diluted 10-fold in the cell culture medium resulting in 2 mg/mL SIJD3 solutions. The medium was then aspirated from the cells and replaced with the 2 mg/mL diluted sample media directly or serial 2-fold dilutions of the sample, 0.05–0.2 mg/mL sodium dodecyl sulfate (SDS) as positive controls, or fresh culture medium as negative controls. The background corresponded to wells containing no cells but only the culture medium. The cells were incubated at 37 °C (5% CO_2) for 24 h, and then the media was removed and 110 μL of fresh media containing 0.5 mg/mL (3-(4,5-dimethylthiazol-2-yl)-2,5-diphenyltetrazolium bromide) (MTT) was added to each well. After 4 h at 37 °C (5% CO_2), the solution was carefully aspirated from the wells and then the purple crystals in each well were dissolved in 50 μL of spectroscopic grade dimethyl sulfoxide (DMSO). The wells were mixed and then their absorbance at 540 nm was read (Biochrom Asys UVM340 plate reader). The background was subtracted, and then for normalization, the mean absorbance of wells containing cells in the fresh culture medium was considered as 100% cell viability. SDS concentrations were evaluated in four replicates and each dilution was evaluated in six replicates. The data are presented as the mean ± standard deviation.

Synthesis

Synthesis of Compound 1.

Phosgene solution (67.8 mL, 0.102 mol, 3.00 eq) was added to a 100 mL flame-dried Schlenk flask equipped with a rubber septum under a nitrogen atmosphere. In a separate round bottom flask, 2-

nitrobenzyl alcohol (13.1 g, 0.0858 mol, 2.50 equiv) was dissolved in 10 mL of dry CH_2Cl_2 and the solution was then added to the phosgene solution drop-wise. The reaction was stirred overnight and then concentrated *in vacuo* to obtain a white powder. ^{1}H NMR spectroscopic analysis indicated the product was ~36% converted to chloroformate. It was used in the next step (in excess) without further purification.

Synthesis of Compounds 2 and 3.

To a 100 mL flame-dried Schlenk flask under a nitrogen atmosphere, freshly distilled propargyl alcohol initiator (0.659 mL, 0.0114 mol, 1.00 equiv.) was dissolved in 15 mL of dry CH_2Cl_2. Purified ethyl glyoxylate (3.20 mL, 0.0343 mol, 3.00 equiv.) was then added to this flask, and the resulting solution was stirred for 20 min at room temperature. The flask was subsequently cooled to -20 °C and stirred for 30 min. Freshly distilled NEt_3 (12.8 mL, 0.0914 mol, 8.00 equiv) was then added to the polymerization flask, and the solution was stirred for another 20 min. 2-Nitrobenzyl chloroformate **(Compound 1)** end-cap (active species 6.60 g, 0.0306 mol, 2.69 equiv) was directly dissolved in 5 mL of dry CH_2Cl_2 and cooled to -20 °C before the solution was added into the polymerization reaction flask. The reaction mixture was sealed under nitrogen, stirred for 5 min and then put in -20 °C freezer for 16 h. Then, the reaction mixture was allowed to warm up to room temperature and was put under vacuum to remove solvent to yield a crude yellow sticky liquid. Purification of crude was performed by silica gel column chromatography with a gradient eluent system (5 v/v% to 10v/v% ethyl acetate in hexane) to obtain **Compound 2** (15% yield) and **3** (4% yield).

Compound 2: ^{1}H NMR (600 MHz, $CDCl_3$) δ 8.20 (d, J = 8.4 Hz, 1H), 7.74–7.71 (m, 2H), 7.56–7.52 (m, 1H), 6.34–6.21 (multiple s, 1H, stereoisomers), 5.69 (s, 2H), 5.55–5.45 (multiple s, 1H, stereoisomers), 4.57–4.42 (m, 2H), 4.37–4.24 (m, 4H), 2.60–2.49 (m, 1H), 1.38–1.31 (m, 6H). ^{13}C NMR (151 MHz, $CDCl_3$) δ 165.6, 165.5, 164.7, 164.5, 153.1, 146.9, 134.1, 131.37, 131.26, 129.7, 129.2, 128.5, 125.23, 125.21, 95.9, 94.7, 92.3, 91.0, 77.5, 77.4, 76.4, 76.3, 66.8, 62.7, 62.3, 55.4, 55.1, 14.0. MS (*m/z*): calcd, 462.1012; found, 462.0587.

Compound 3: ^{1}H NMR (600 MHz, $CDCl_3$) δ 8.20 (d, J = 8.4 Hz, 1H), 7.77–7.72 (m, 2H), 7.55–7.52 (m, 1H), 6.39–6.23 (multiple s, 1H, stereoisomers), 5.79–5.38 (multiple s, 5H, stereoisomers), 4.59 4.08 (m, 10H), 2.51–2.49 (m, 1H), 1.33 (s, 12H). ^{13}C NMR (151 MHz, $CDCl_3$) δ 165.6–164.2 (multiple peaks, stereoisomers), 153.1, 146.9, 134.4, 131.4, 129.0, 128.4, 125.2, 94.8–92.1 (multiple peaks, stereoisomers), 78.0, 75.8, 66.80, 66.7, 62.3, 55.7–55.0 (multiple peaks, stereoisomers), 14.0. MS (*m/z*): calcd, 666.1646; found, 666.1606.

Synthesis of Compound 4.

NaOH (0.2 mol) was dissolved in 40 mL of Milli-Q water. Separately, tri(ethylene glycol) monomethyl ether (0.14 mol) was dissolved in 40 mL of THF. The two solutions were then combined under stirring, forming a white turbid emulsion. The resulting mixture was cooled in an ice bath. In parallel, *p*-toluenesulfonyl chloride (TsCl, 0.13 mol) was dissolved in 40 mL of THF

and then transferred to a dropping funnel and added dropwise to the cold emulsion under continuous stirring. After complete addition, the reaction mixture was stirred at ice bath for an additional 2 h. The reaction was then stopped by pouring into ice water. The resulting mixture was extracted with DCM. The organic extractant was then washed with water (twice) and once with saturated brine, then dried over anhydrous Na_2SO_4. **Compound 4** (92% yield), an oil product, was obtained after drying under vacuum.

Synthesis of Compounds 5 and 6.

Compound 5: To a 250 mL round-bottom flask, methyl 3,5-dihydroxybenzoate (15 mmol), K_2CO_3 (90 mmol), Compound 4 (58.5 mmol), and KI (9 mmol) were added, followed by the addition of 70 mL of dry DMF. The mixture was purged with nitrogen by bubbling for 30 min, heated to 80 °C and stirred under a nitrogen atmosphere overnight. The reaction progress was monitored by TLC. Upon completion, the reaction mixture was allowed to cool to room temperature and diluted with 100 mL of water, yielding a yellow, semi-clear solution. The aqueous mixture was extracted multiple times with DCM, and the combined organic extracts were dried over anhydrous Na_2SO_4, resulting in a transparent yellow solution. After filtration, the solvent was removed under vacuum to yield a slightly viscous yellow oil. The crude product was purified by column chromatography using ethyl acetate/heptane (1.5:1) as the eluent, yielding **Compound 5** (71% yield).

Compound 6 (68% yield) was synthesized following a similar procedure with methyl 3,5-dihydroxybenzoate being used.

Synthesis of Compounds 7 and 8.

Compound 7: **Compound 5** (6 mmol) and KOH (30 mmol) were dissolved in 95% EtOH solvent. The reaction mixture was heated to 80 °C and stirred for 2 h. Reaction progress was monitored by TLC. Upon completion, the reaction was stopped and the solvent was evaporated under reduced pressure, yielding an orange liquid. The residue was poured into water, followed by adjustment of the pH to ~1 using hydrochloric acid. This resulted in a clear yellow aqueous solution, which was extracted with DCM. The organic extracts were dried over anhydrous Na_2SO_4. The filtrate was concentrated under reduced pressure, and the resulting residue was dried under vacuum to afford **Compound 7** (93% yield).

Compound 8 (92% yield) was synthesized following a similar procedure with **Compound 6** being used.

Synthesis of Compound 9.

To a stirred solution of 2,2-bis(bromomethyl)-1,3-propanediol (12 mmol) in anhydrous DMSO (9 mL) was added NaN_3 (30 mmol) under a nitrogen atmosphere. The resulting suspension was heated to 110 °C and stirred for approximately 16 h under N_2. After cooling to room temperature, reaction mixture was diluted with water. And the resulting brown aqueous solution was transferred

to a separatory funnel and extracted with ethyl acetate. The extracted organic phases were washed with saturated brine and then dried over anhydrous $MgSO_4$. After drying under vacuum, the product was obtained as a pale yellow oil (92% yield).

NMR characterization of **Compounds 4−9** are in agreement with previously reported data.[2-3]

Synthesis of Compounds 10 and 11.

Compound 10: A Schlenk flask (50 mL) was charged with **Compound 9** (0.6 mmol), **Compound 7** (1.38 mmol), and 4-(dimethylamino)pyridinium 4-toluenesulfonate (DPTS, synthesized as described previously with DMAP and PTSA,[4] 0.6 mmol), followed by evacuation and refilling with nitrogen. The flask was cooled in an ice-water bath, and 12 mL of dry DCM was added slowly.The reaction was performed under nitrogen at room temperature. TLC analysis was used to monitor the reaction. Upon observing incomplete conversion, additional reagents were added. Upon completion of the reaction, the light yellow turbid mixture was filtered and DCM was removed under reduced pressure, yielding the crude product as a yellow oil. The crude product was further purified by column chromatography using a gradient eluent of ethyl acetate and methanol, yielding **Compound 10** (90% yield, light yellow oil).

Compound 11 (81% yield) was synthesized following a similar procedure with **Compound 8** being used.

Compound 10: ^{1}H NMR (600 MHz, $CDCl_3$) δ 7.15 (s, 4H), 6.73 (s, 2H), 4.36 (s, 4H), 4.18 – 4.14 (m, 8H), 3.88 (t, *J* = 4.6 Hz, 8H), 3.76 (t, *J* = 4.6 Hz, 8H), 3.71 (t, *J* = 4.7 Hz, 8H), 3.67 (t, *J* = 4.5 Hz, 8H), 3.61 (s, 4H), 3.56 (t, *J* = 4.7 Hz, 8H), 3.39 (s, 12H). ^{13}C NMR (150 MHz, $CDCl_3$) δ 165.6, 159.9, 131.1, 108.2, 106.9, 71.9, 70.8, 70.7, 70.6, 69.6, 67.8, 63.4, 59.0, 51.6, 43.6. MS (*m/z*): calcd, 1065.4856; found, 1065.4804.

Compound 11: ^{1}H NMR (600 MHz, CDCl3) δ 7.62 (dd, *J* = 8.4, 2.1 Hz, 2H), 7.56 (d, *J* = 2.1 Hz, 2H), 6.92 (d, *J* = 8.5 Hz, 2H), 4.37 (s, 4H), 4.24–4.20 (m, *J* = 5.0 Hz, 8H), 3.90 (t, *J* = 5.2 Hz, 8H), 3.82–3.74 (m, 8H), 3.72–3.68 (m, 16H), 3.61 (s, 4H), 3.56 (t, J = 4.7 Hz, 8H), 3.39 (s, 12H). ^{13}C NMR (151 MHz, CDCl3) δ 165.6, 153.4, 148.5, 124.0, 122.1, 115.2, 112.7, 72.0, 70.94, 70.89, 70.7, 70.6, 69.6, 69.5, 69.0, 68.6, 63.2, 59.1, 59.0, 51.8, 43.6. MS (*m/z*): calcd, 1065.4856; found, 1065.4812.

SIJD1: ^{1}H NMR (600 MHz, $CDCl_3$) δ 8.16 – 8.15 (m, 2H), 8.03–7.98 (m, 2H), 7.71–7.69 (m, 4H), 7.60–7.58 (m, 2H), 7.54–7.51 (m, 4H), 6.91 (d, *J* = 7.84 Hz 2H), 6.35–6.24 (multiple s, 2H, stereoisomers), 5.78–5.59 (multiple s, 8H, stereoisomers), 5.48–5.40 (multiple s, 2H, stereoisomers), 4.97–4.83 (m, 4H), 4.70–4.65 (m, 4H), 4.38–4.14 (m, 32H), 3.96–3.86 (m, 8H), 3.78–3.76 (m, 8H), 3.70–3.65 (m, 16H), 3.57–3.54 (m, 8H), 3.40–3.38 (m, 12H), 1.38–1.27 (m, 24H). ^{13}C NMR (151 MHz, $CDCl_3$) δ 165.7–164.2 (multiple peaks, stereoisomers), 159.9, 153.0, 146.9, 134.2, 131.4, 131.3, 130.8, 129.0, 128.6, 125.1, 124.1, 108.3, 106.9, 94.3–92.4 (multiple peaks, stereoisomers), 71.9, 70.9, 70.7, 70.6, 69.6, 69.5, 69.0, 68.6, 66.8, 63.0–62.0 (multiple peaks, stereoisomers), 59.0, 49.7, 44.3, 13.9. MS (m/z): calcd, 2351.8353; found, 2351.8418.

SIJD2: ^{1}H NMR (600 MHz, $CDCl_3$) δ 8.16 (d, *J* = 8.4 Hz, 2H), 8.10–8.00 (m, 2H), 7.72–7.69 (m, 4H), 7.52–7.50 (m, 2H) 7.15 (s, 4H), 6.74–6.70 (m, 2H), 6.34–6.23 (multiple s, 2H, stereoisomers), 5.78–5.58 (multiple s, 8H, stereoisomers), 5.49–5.38 (multiple s, 2H, stereoisomers), 4.91–4.80 (m, 4H), 4.67–4.66 (m, 4H), 4.32–4.12 (m, 28H), 3.88–3.87 (m, 8H), 3.76–3.65 (m, 24H), 3.56–3.54 (m, 8H), 3.39 (s, 12H), 1.32–1.26 (m, 24H). ^{13}C NMR (151 MHz, $CDCl_3$) δ 166.3–164.4 (multiple peaks, stereoisomers), 160.0, 153.0, 146.9, 134.2, 131.4. 130.8, 129.0, 128.6, 125.1, 108.3, 106.9, 94.7–92.4 (multiple peaks, stereoisomers), 71.4, 70.8, 70.6, 69.6, 67.8, 66.8, 62.7–62.0 (multiple peaks, stereoisomer), 59.0, 49.7, 44.3, 13.9. MS (m/z): calcd, 2351.8353; found, 2351.8448.

SIJD3: ^{1}H NMR (600 MHz, $CDCl_3$) δ 8.15 (d, *J* = 8.4 Hz, 2H), 8.11–7.98 (m, 2H), 7.73–7.69 (m, 4H), 7.52–7.50 (m, 2H), 7.15–7.14 (m, 4H), 6.73 (s, 2H), 6.28–6.22 (multiple s, 2H, stereoisomers), 5.67–6.58 (m, 4H), 5.43–5.37 (multiple s, 2H, stereoisomers), 4.94–4.82 (m, 4H), 4.67–4.66 (m, 4H), 4.32–4.19 (m, 12H), 4.16–4.13 (m, 8H), 3.88–3.86 (m, 8H), 3.77–3.65 (m, 24H), 3.56–3.54 (m, 8H), 3.38–3.37 (m, 12H), 1.31–1.26 (m, 12H). ^{13}C NMR (151 MHz, $CDCl_3$) δ 165.3–163.5 (multiple peaks, stereoisomers), 159.9, 156.3, 134.2, 129.0, 128.6, 127.8, 125.2, 113.9, 108.3, 106.9, 95.0–92.3 (multiple peaks, stereoisomers), 71.9, 70.8, 70.64, 70.56, 69.6, 68.8, 68.6, 67.8, 66.8, 63.7, 62.4–62.2 (multiple peaks, stereoisomers), 59.0, 41.7, 13.9. MS (m/z): calcd, 1943.7085; found, 1943.7095.

DLS UV Degradation Data

Table S1. Averaged triplicate (with standard deviation) experimental (UV irradiated) vs control data for **SIJD1, 2 and 3** including *Z*-average, PDI, and derived count rate (attenuator index fixture of 5) acquired from DLS at t = 0, 0.5, 1, 2, 3, 4, 8, 16, 32 min.

**(SIJD1)**	***Z*-average (nm)**				**PDI**				**derived count rate (kcps)**			
Time (min)	Control	stdev	Exp	stdev	Control	stdev	Exp	stdev	Control	stdev	Exp	stdev
0	209.2	3.1	219.6	13.1	0.157	0.027	0.188	0.055	256.9	7.4	287.7	25.2
0.5	209.8	1.6	209.4	5.4	0.134	0.008	0.155	0.048	257.9	2.0	254.7	15.6
1	209.9	1.3	206.1	13.7	0.154	0.023	0.186	0.066	271.3	14.4	230.3	18.3
2	207.8	1.9	197.8	6.5	0.152	0.036	0.133	0.019	269.2	5.4	201.6	14.8
3	208.7	3.0	188.2	4.0	0.156	0.016	0.129	0.020	262.6	1.4	171.4	1.1
4	224.0	1.6	181.6	2.5	0.228	0.013	0.105	0.018	285.0	0.7	140.5	25.0
8	211.9	4.3	180.7	9.3	0.174	0.024	0.141	0.023	269.7	5.4	112.2	12.3
16	230.5	14.2	188.0	35.4	0.199	0.025	0.162	0.062	267.5	10.9	88.2	19.6
32	225.0	5.7	169.5	10.9	0.145	0.038	0.080	0.068	280.6	13.9	75.4	13.4
**(SIJD2)**	***Z*-average (nm)**				**PDI**				**derived count rate (kpcs)**			
Time (min)	Control	stdev	Exp	stdev	Control	stdev	Exp	stdev	Control	stdev	Exp	stdev
0	150.5	5.3	150.0	9.1	0.089	0.017	0.082	0.063	264.0	1.9	241.3	18.7
0.5	153.0	1.6	151.6	1.7	0.050	0.018	0.062	0.035	272.7	2.7	233.4	3.9
1	154.8	1.8	148.7	0.9	0.056	0.034	0.030	0.031	276.5	0.9	220.6	9.7
2	156.3	1.9	141.7	2.8	0.041	0.025	0.047	0.057	270.1	1.8	180.6	21.1
3	159.5	0.8	132.8	0.2	0.019	0.004	0.036	0.005	271.7	1.1	139.1	3.6
4	161.6	1.6	128.7	2.9	0.028	0.022	0.023	0.023	278.8	1.1	113.2	1.6
8	165.2	1.6	114.9	1.3	0.028	0.021	0.067	0.013	286.2	0.5	72.2	3.8
16	166.8	1.3	114.5	12.1	0.021	0.020	0.179	0.078	285.0	0.6	48.2	8.0
32	169.1	1.3	94.4	4.3	0.023	0.010	0.120	0.036	282.0	0.7	29.3	3.2
**(SIJD3)**	***Z*-average (nm)**				**PDI**				**derived count rate (kcps)**			
Time (min)	Control	stdev	Exp	stdev	Control	stdev	Exp	stdev	Control	stdev	Exp	stdev
0	186.8	3.1	203.2	17.8	0.050	0.019	0.102	0.065	273.5	47.9	304.7	64.9
0.5	203.4	17.6	196.2	9.1	0.090	0.089	0.102	0.064	263.9	55.7	279.1	5.5
1	197.1	0.7	183.6	1.5	0.048	0.012	0.056	0.036	235.7	53.8	184.6	3.8
2	202.7	4.3	176.2	1.2	0.076	0.054	0.051	0.006	257.1	60.4	173.6	19.2
3	205.4	1.0	165.9	0.4	0.070	0.009	0.060	0.027	258.5	62.3	141.1	15.7
4	207.1	1.6	158.4	3.1	0.064	0.014	0.052	0.012	283.5	1.5	114.6	2.6
8	208.2	1.0	144.7	2.6	0.068	0.017	0.085	0.013	238.6	57.3	78.7	1.6
16	211.5	2.0	139.2	3.5	0.089	0.012	0.113	0.038	262.3	1.3	60.7	6.4
32	214.1	2.3	143.9	21.0	0.100	0.014	0.180	0.060	253.2	2.1	55.8	13.3

^{1}H NMR Spectra

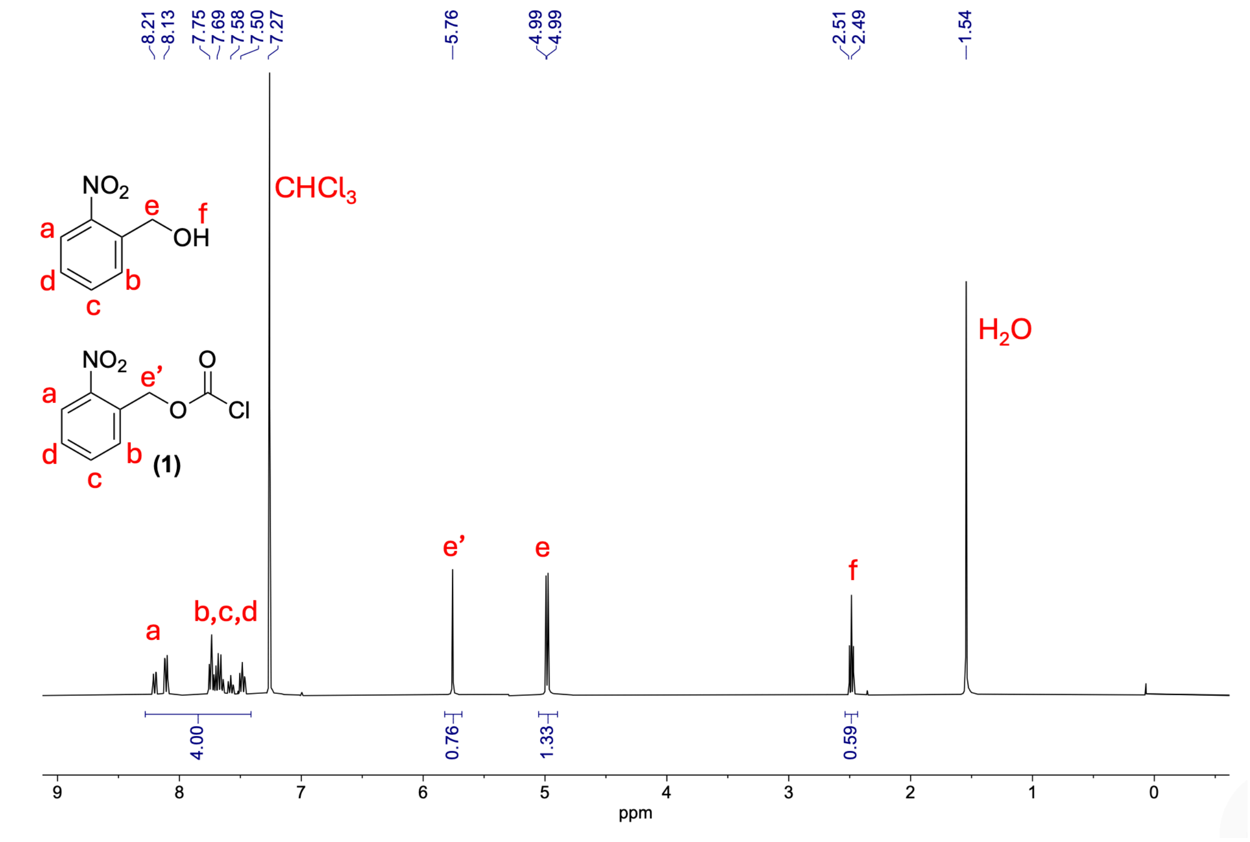

Figure S1. ^{1}H NMR spectrum of **Compound 1** showing ~36% conversion to the chloroformate. (400 MHz, $CDCl_3$).

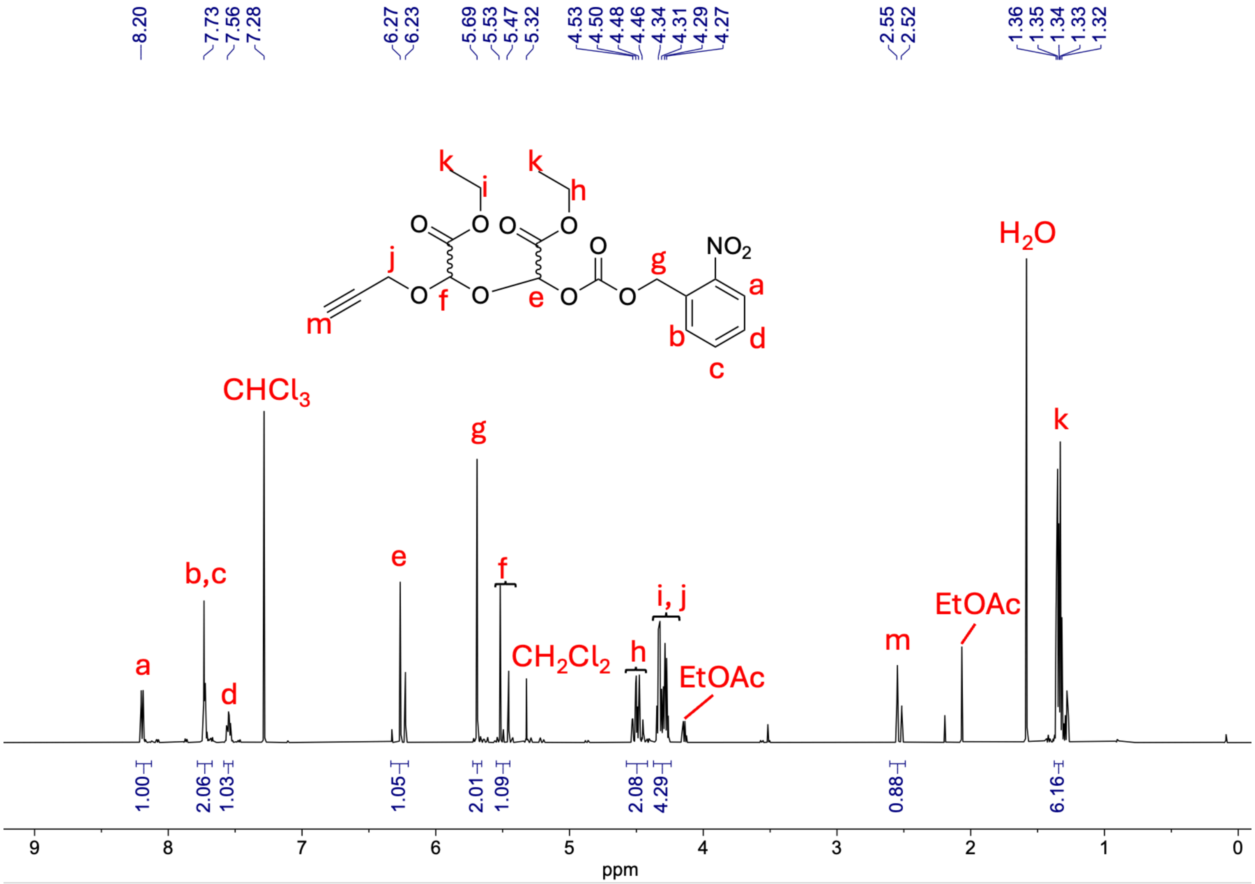

Figure S2. ^{1}H NMR spectrum of **Compound 2** (600 MHz, $CDCl_3$). Note that multiple peaks were observed for e and f due to the presence of stereoisomers. The left most peak of e (representing OEtG backbone) might be due to some dimer that was initiated from water, which resulted in a slightly different chemical shift, but since the species is not reactive during the next step click reaction, the species is removed through purification, as shown in **Figure S14** the OEtG oligomer backbone has only 2 peaks at this chemical shift (two diastereomers).

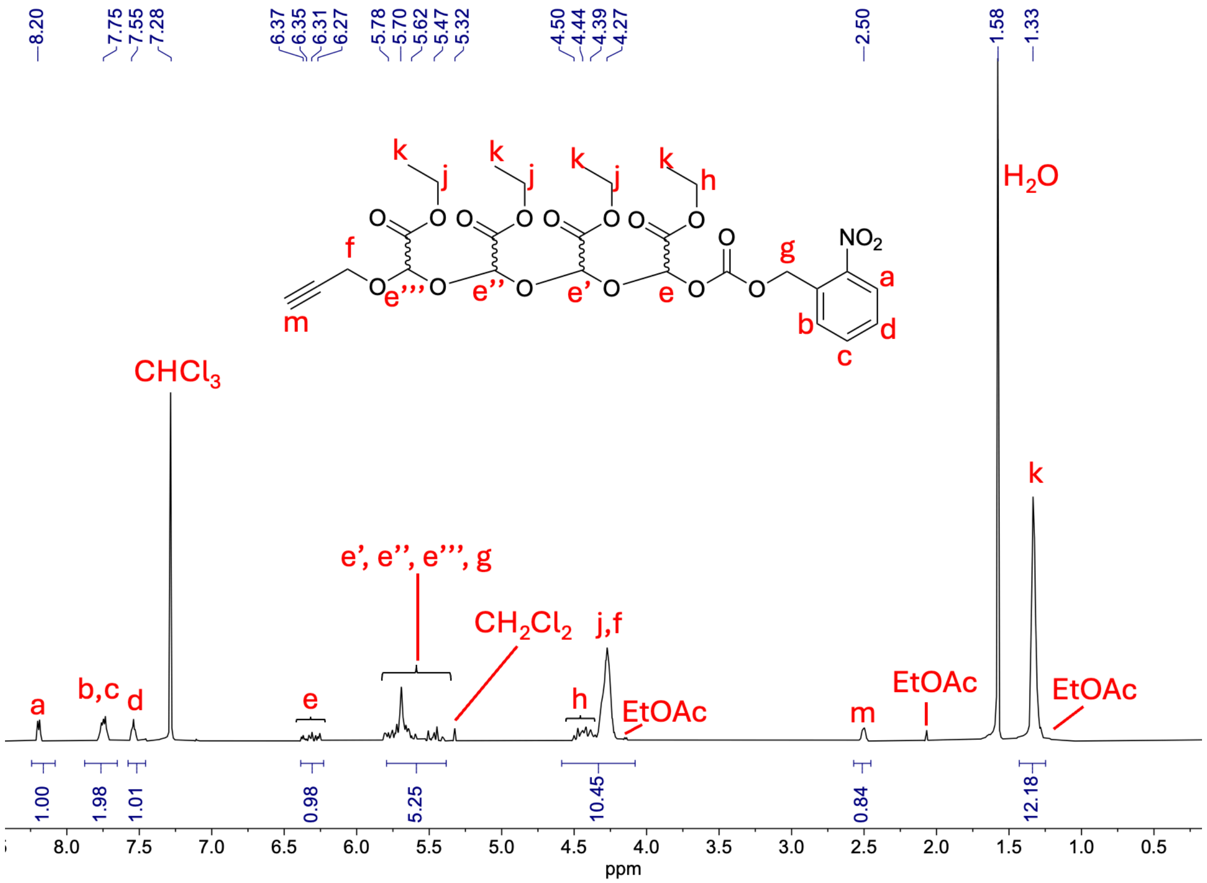

Figure S3. ^{1}H NMR spectrum of **Compound 3** (600 MHz, $CDCl_3$). Note that multiple peaks were observed for e, e', e'', and e''' due to the presence of stereoisomers (8 diastereomers possible).

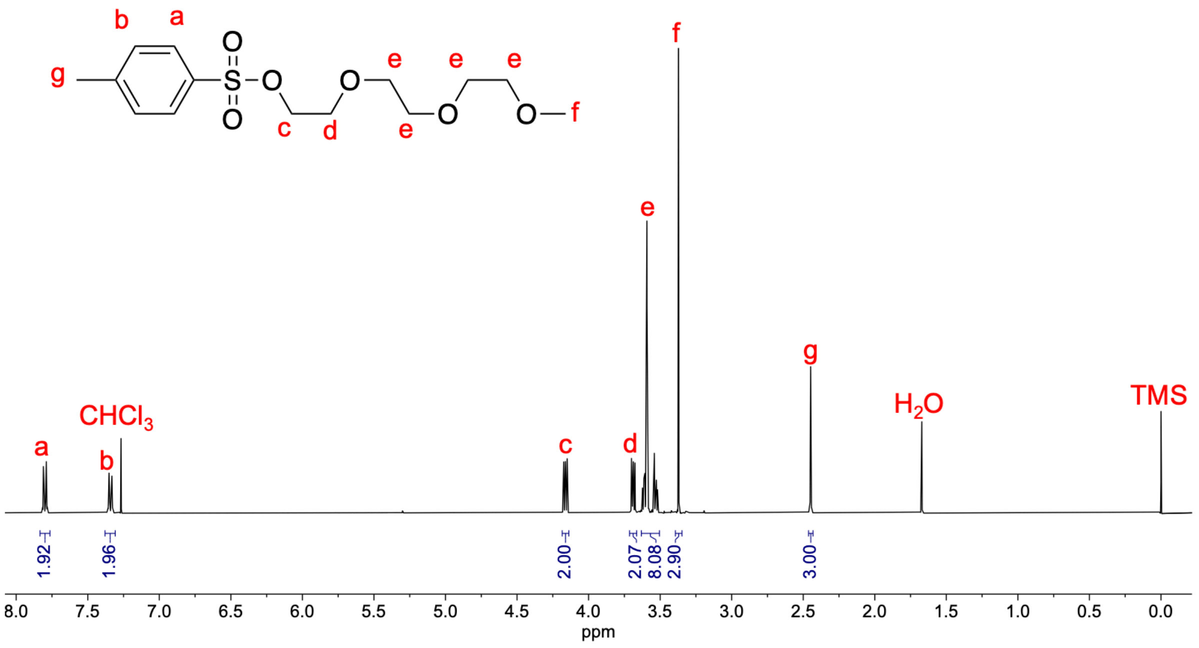

Figure S4. ^{1}H NMR spectrum of **Compound 4** (400 MHz, $CDCl_3$).

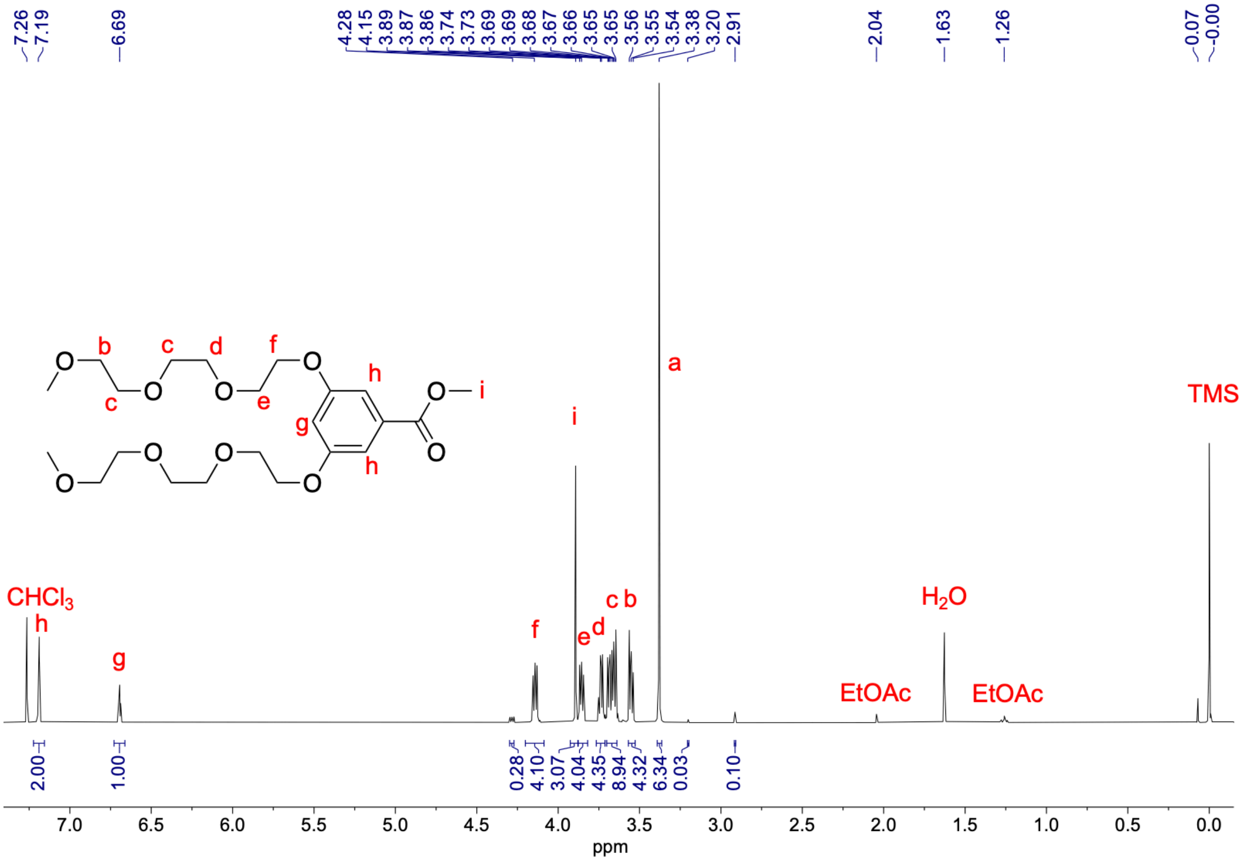

Figure S5. ^{1}H NMR spectrum of **Compound 5** (400 MHz, $CDCl_3$).

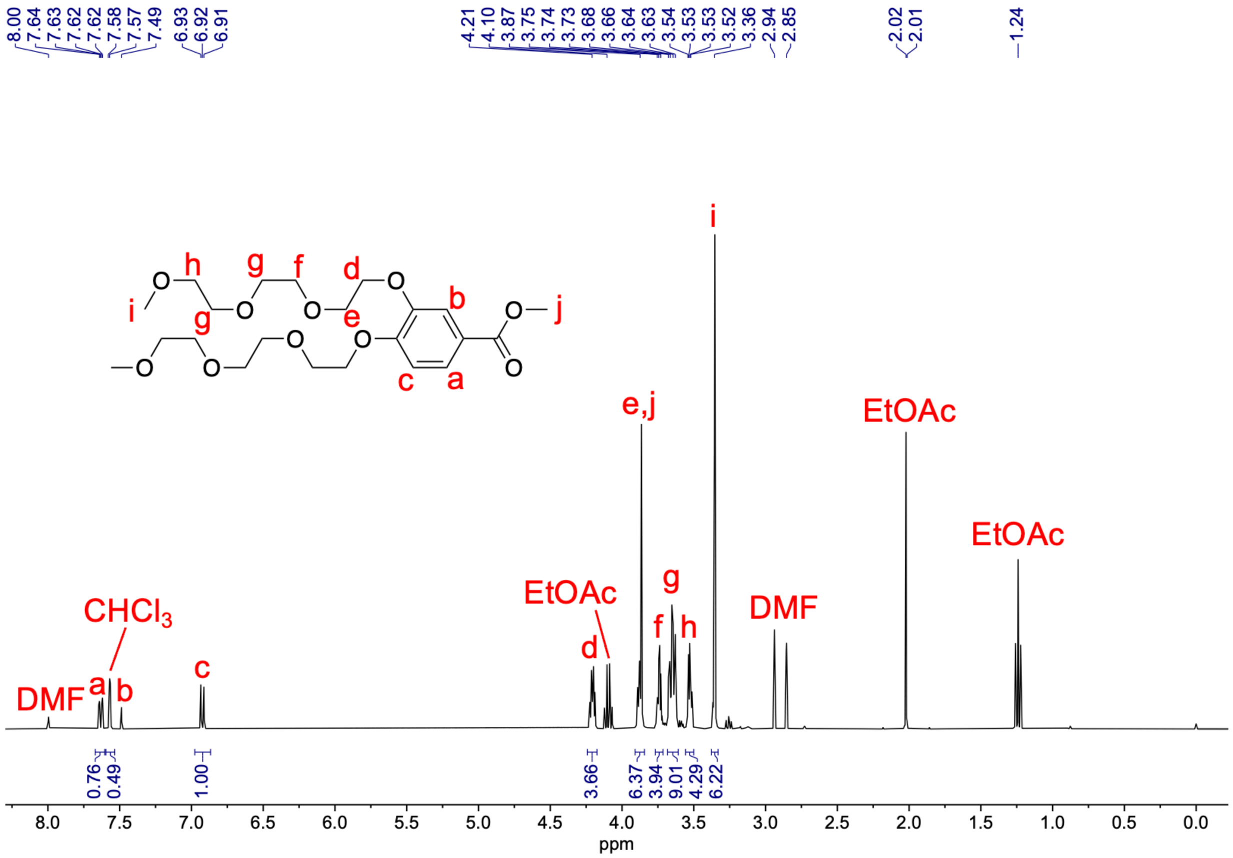

Figure S6. ^{1}H NMR spectrum of **Compound 6** (400 MHz, $CDCl_3$).

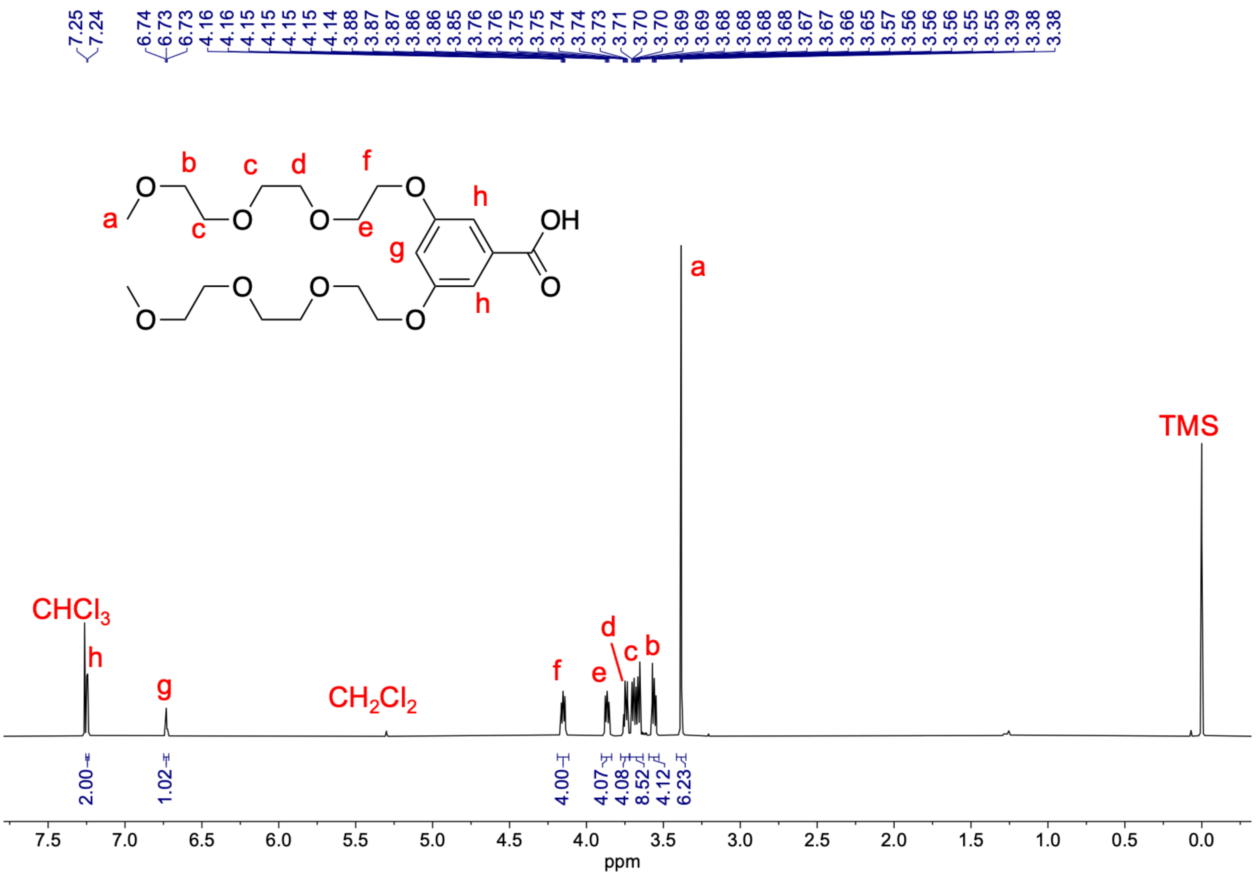

Figure S7. ^{1}H NMR spectrum of **Compound 7** (400 MHz, $CDCl_3$).

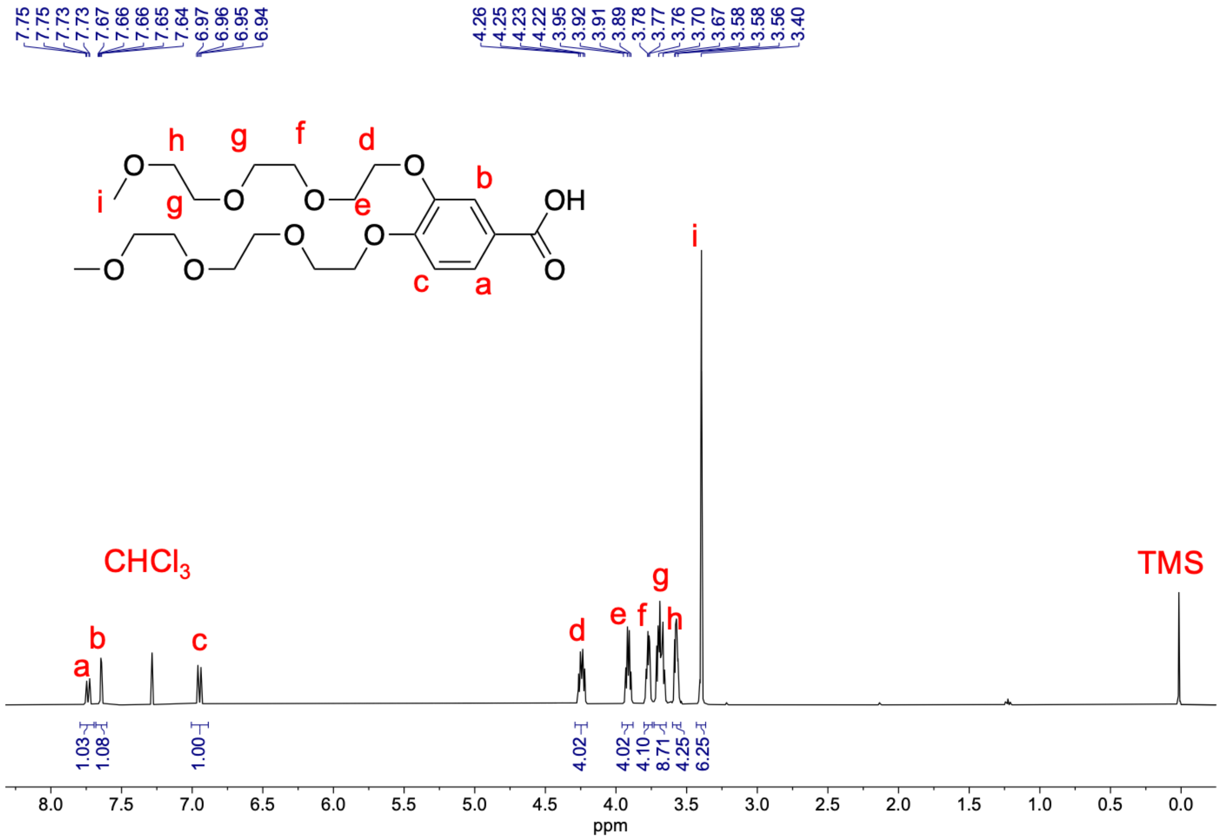

Figure S8. ^{1}H NMR spectrum of **Compound 8** (400 MHz, $CDCl_3$).

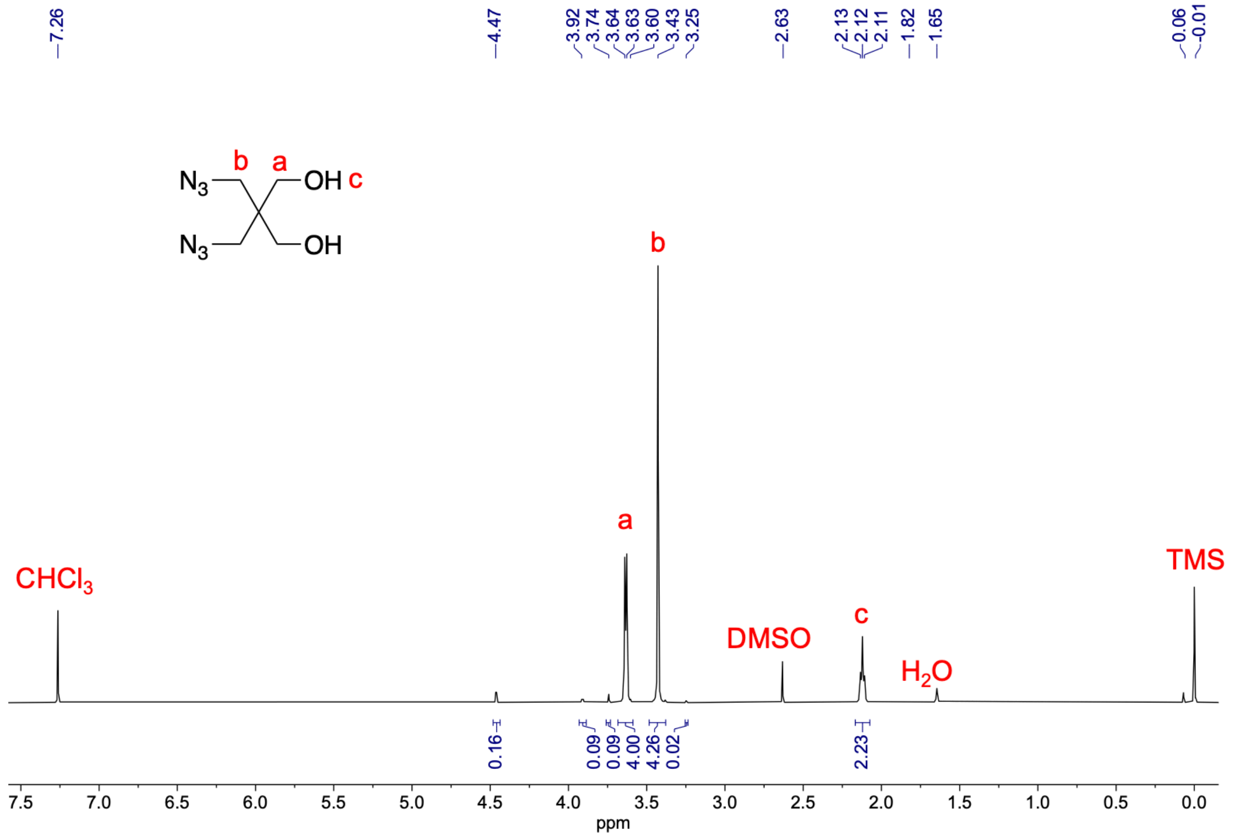

Figure S9. ^{1}H NMR spectrum of **Compound 9** (400 MHz, $CDCl_3$).

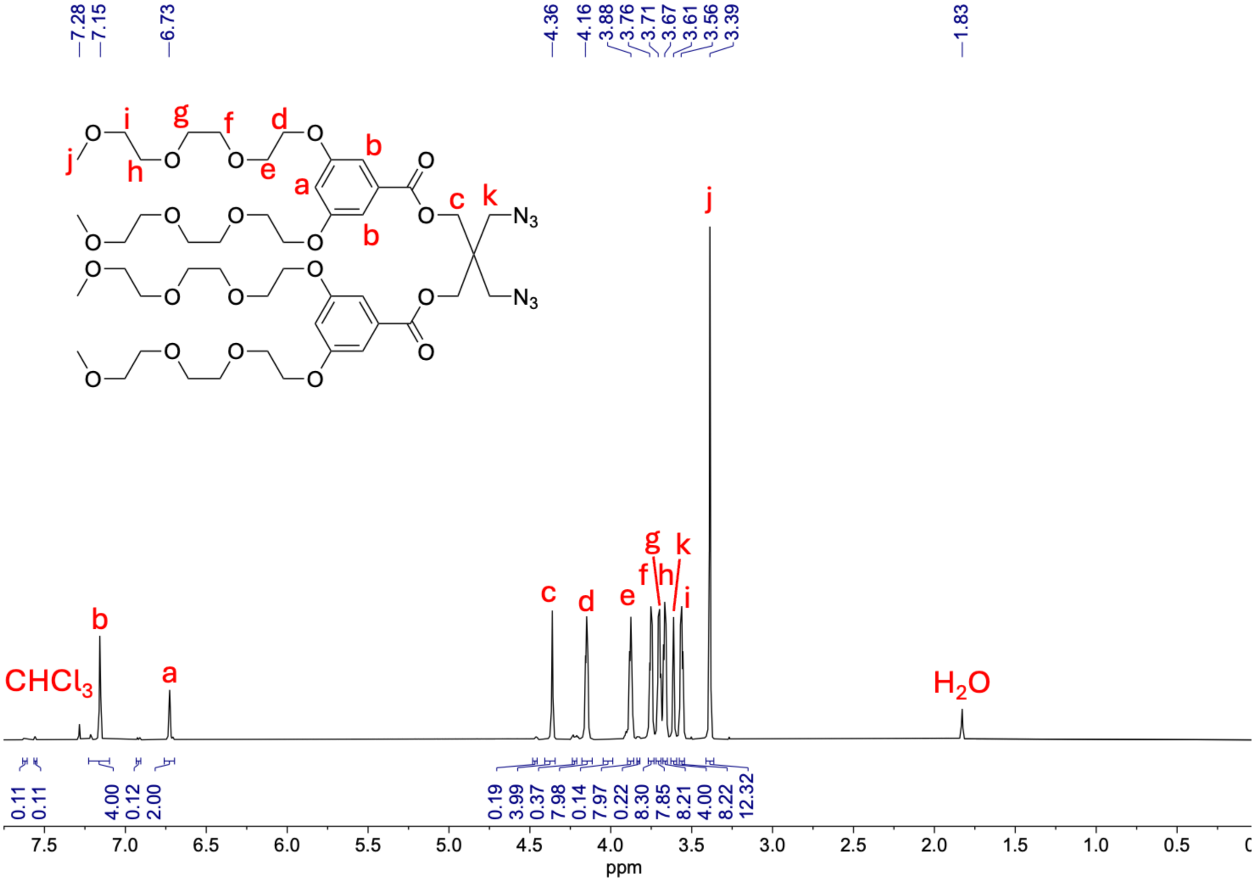

Figure S10. ^{1}H NMR spectrum of **Compound 10** (600 MHz, $CDCl_3$). Note that the water peak is shifted downfield from its expected shift of 1.56 ppm, consistent with previously observations in poly(ethylene glycol) systems.[5] This shift arises from hydrogen bonding between water and the oligo(ethylene glycol) units at the relatively high concentration used in the NMR sample.

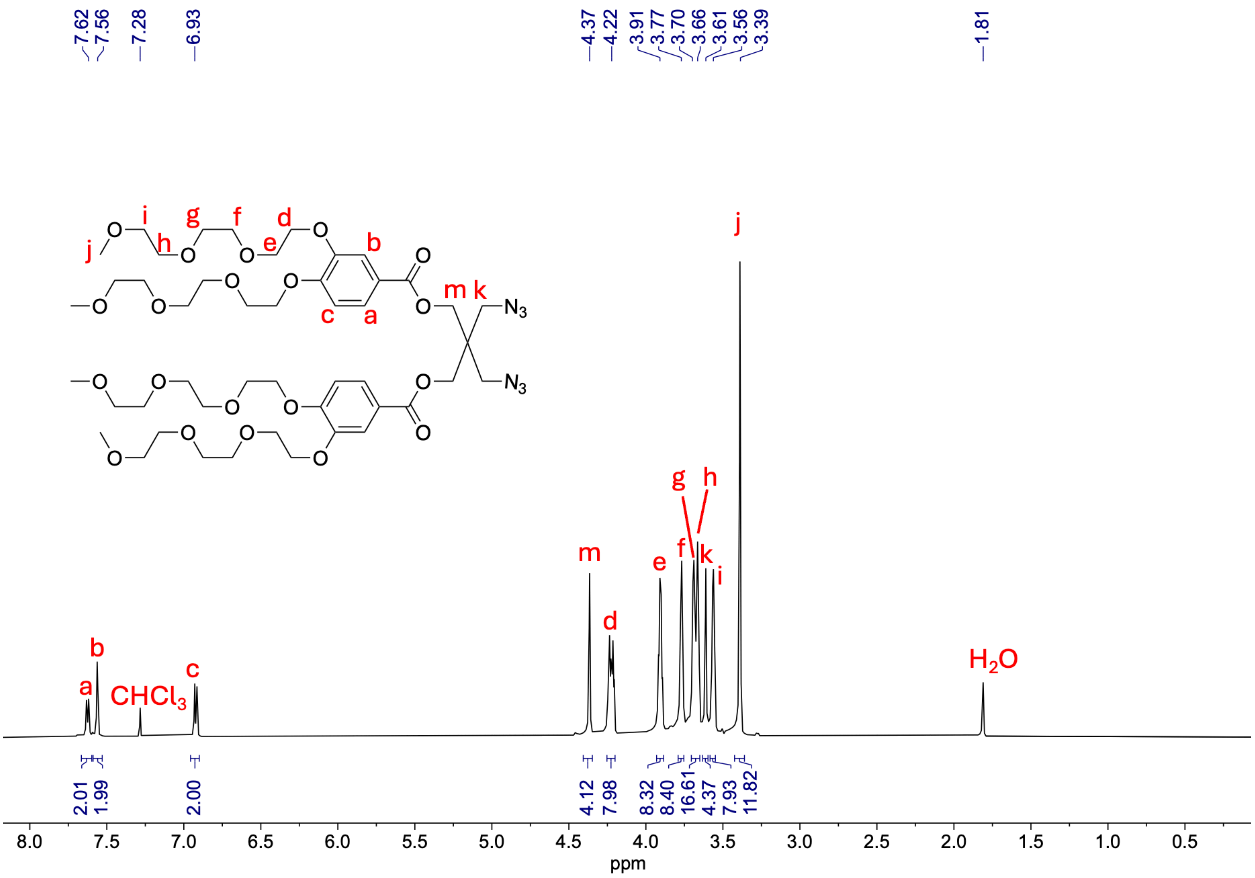

Figure S11. ^{1}H NMR spectrum of **Compound 11** (600 MHz, $CDCl_3$). Note that the water peak is shifted downfield from its expected shift of 1.56 ppm for the same reasons indicated in the caption of **Figure S10**.

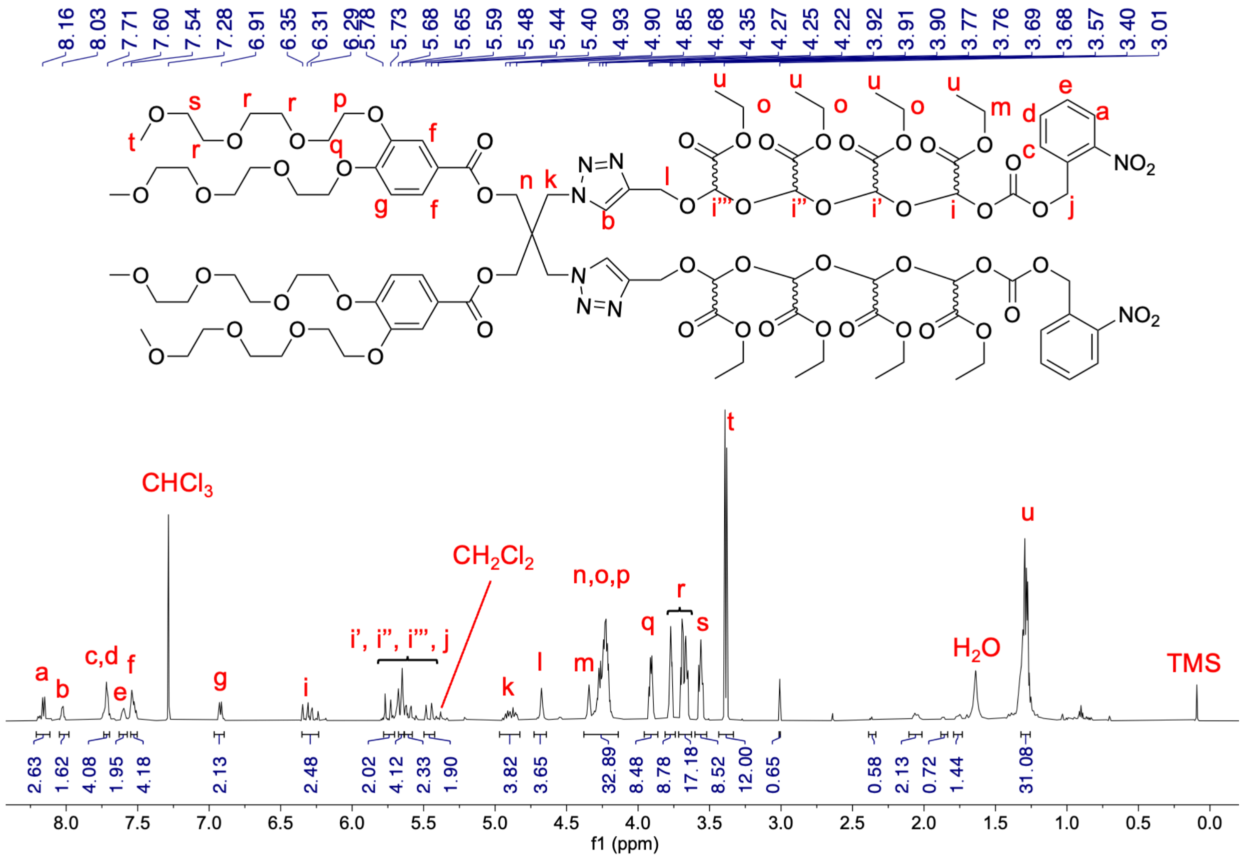

Figure S12. ^{1}H NMR Spectrum of **SIJD1** (600 MHz, CDCl$_3$). Multiple peaks were observed for i, i', i'', and i''' due to the presence of stereoisomers as noted in **Figure S3**.

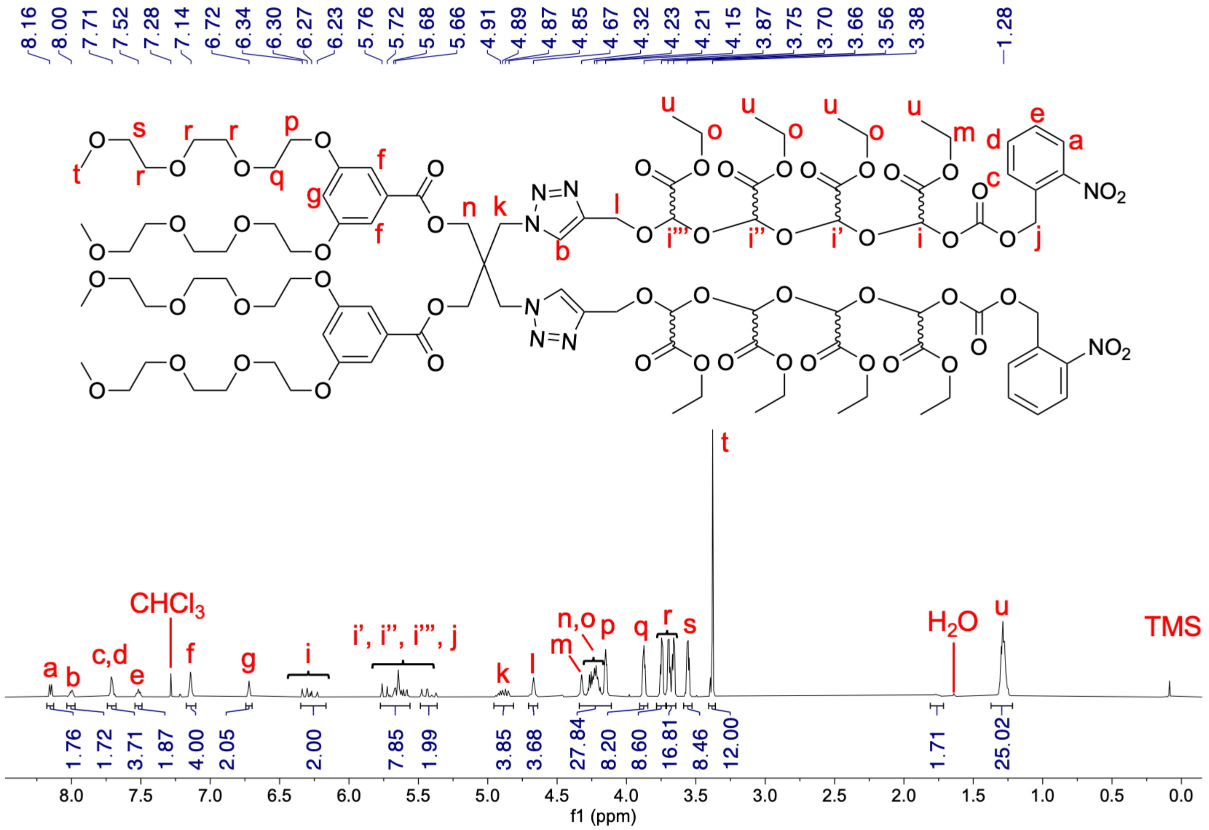

Figure S13. ^{1}H NMR Spectrum of **SIJD2** (600 MHz, $CDCl_3$). Multiple peaks were observed for i, i’, i’’, and i’’’ due to the presence of stereoisomers as noted in **Figure S3**.

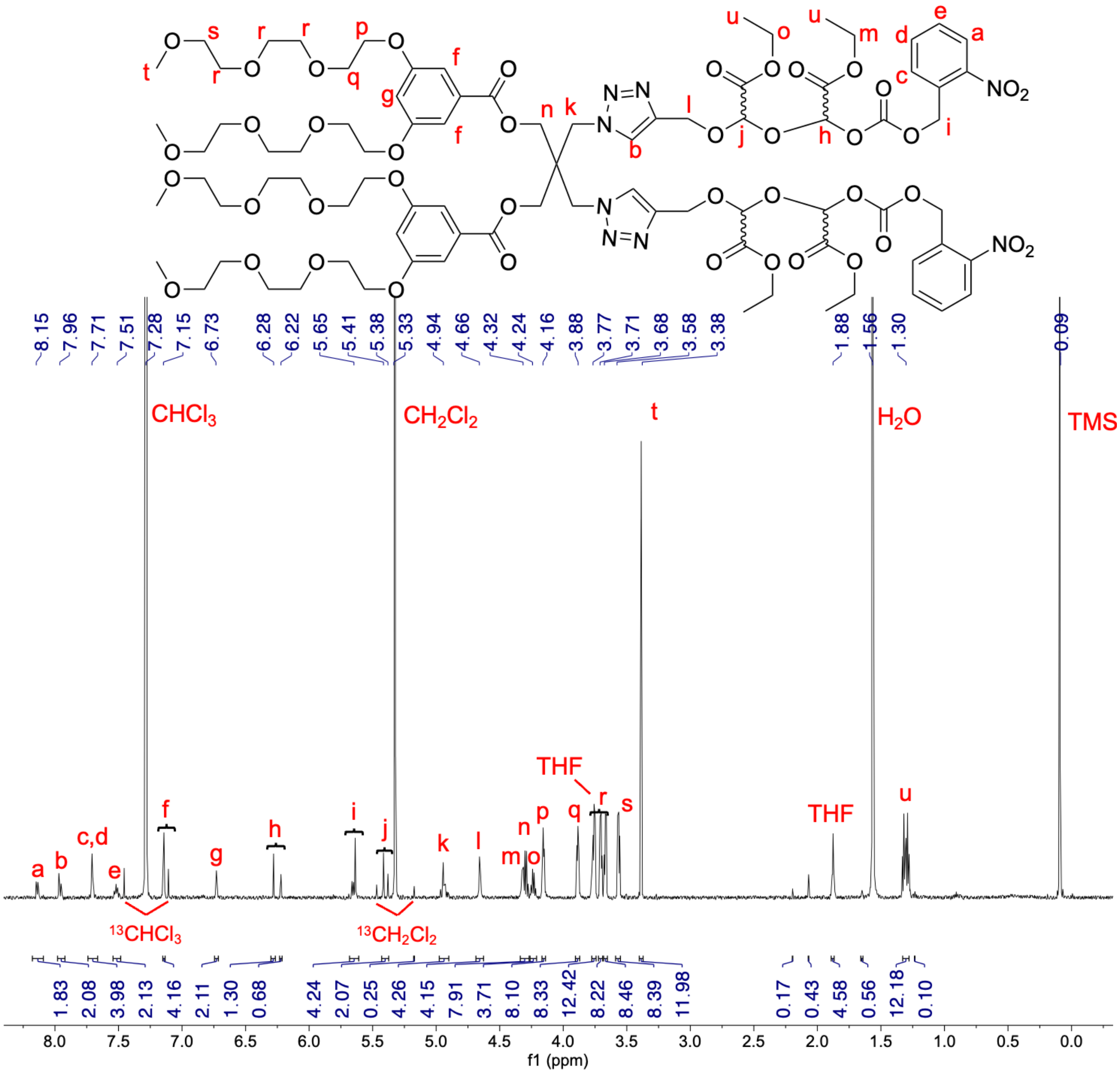

Figure S14. ^{1}H NMR spectrum of **SIJD3** (600 MHz, $CDCl_3$). Multiple peaks were observed for j and h due to the presence of stereoisomers as noted in **Figure S2**.

^{13}C NMR Spectra

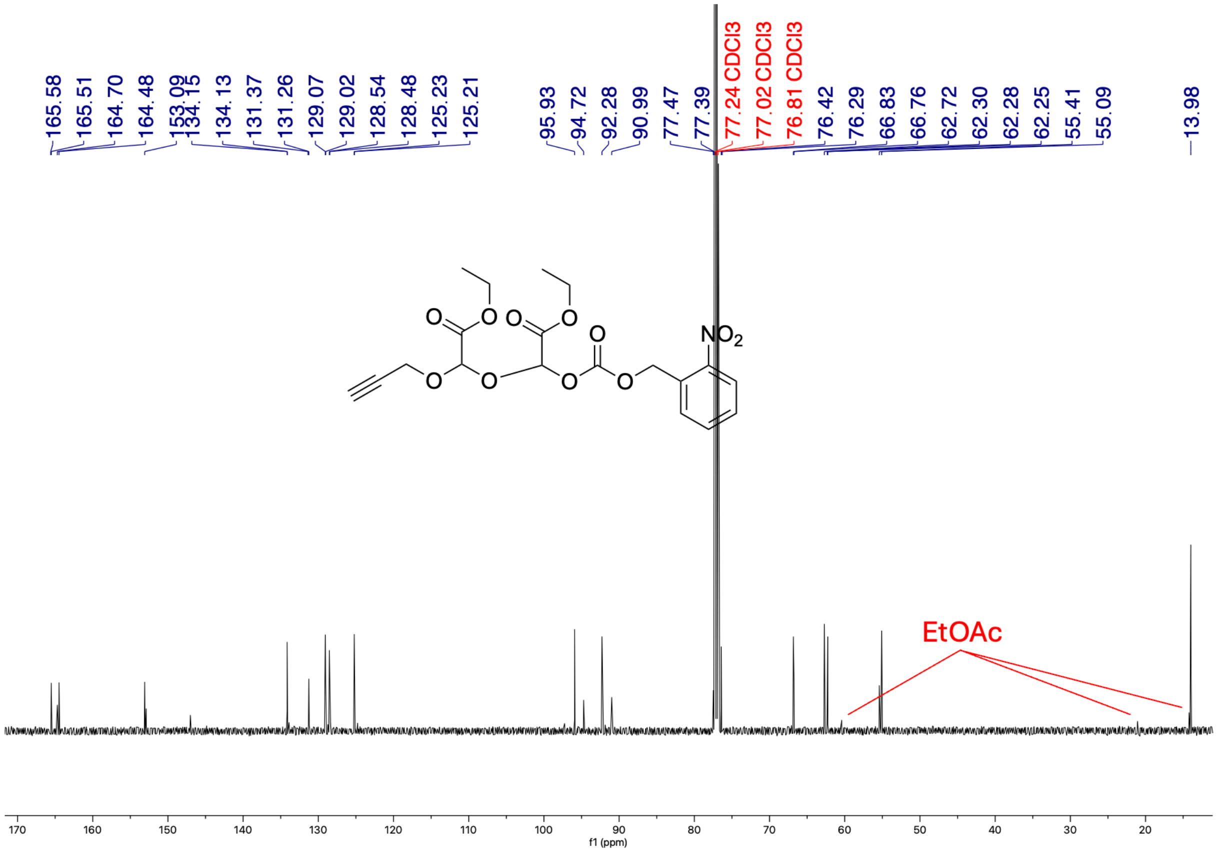

Figure S15. ^{13}C NMR spectrum of **Compound 2** (150 MHz, $CDCl_3$).

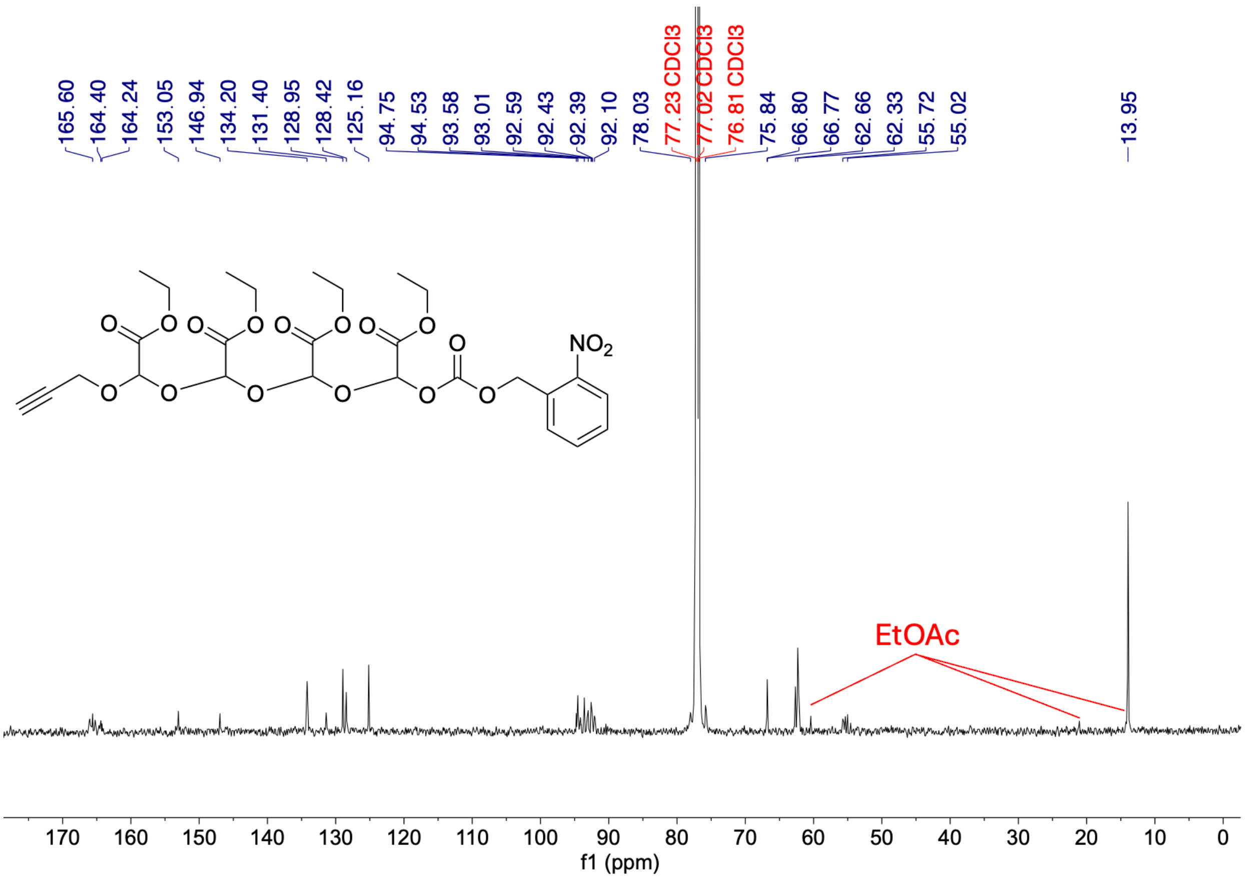

Figure S16. ^{13}C NMR spectrum of **Compound 3** (150 MHz, $CDCl_3$).

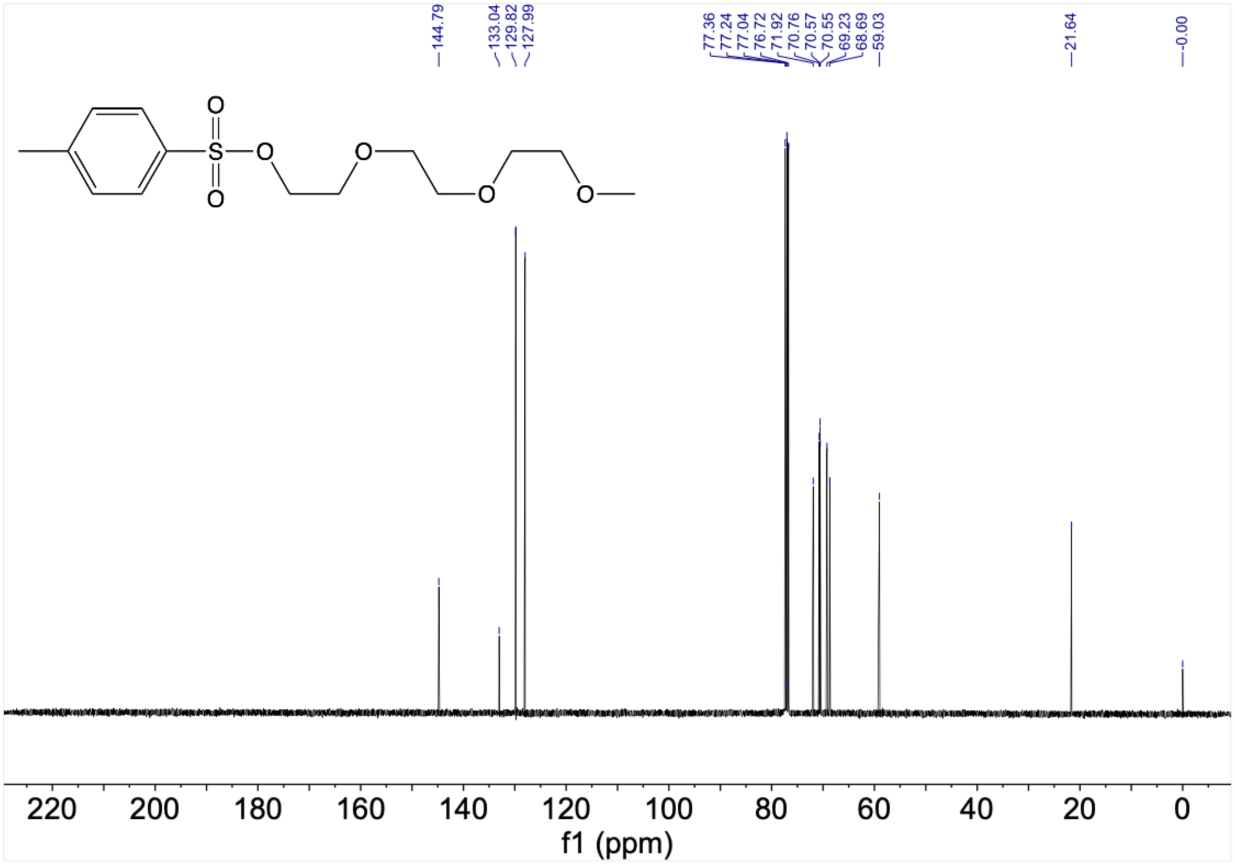

Figure S17. ^{13}C NMR spectrum of **Compound 4** (101 MHz, $CDCl_3$).

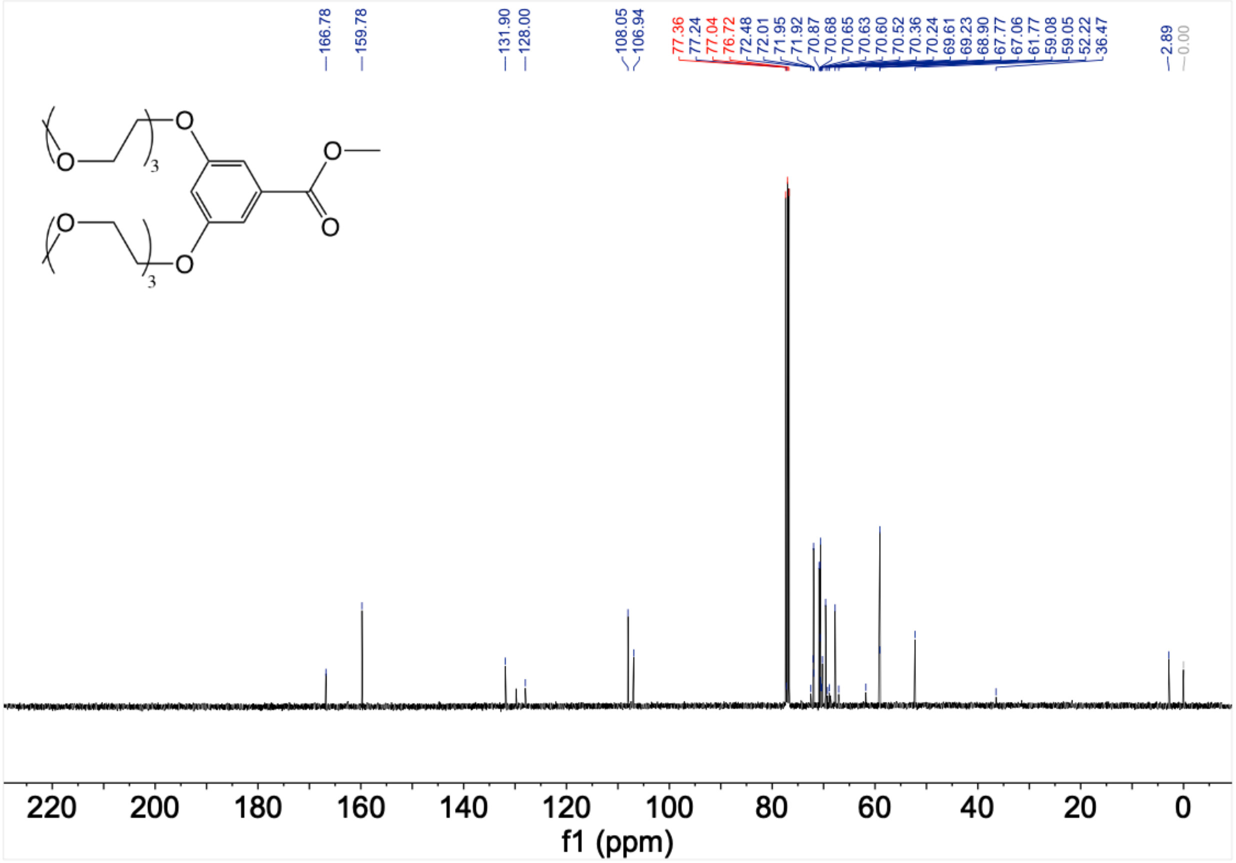

Figure S18. ^{13}C NMR spectrum of **Compound 5** (101 MHz, $CDCl_3$).

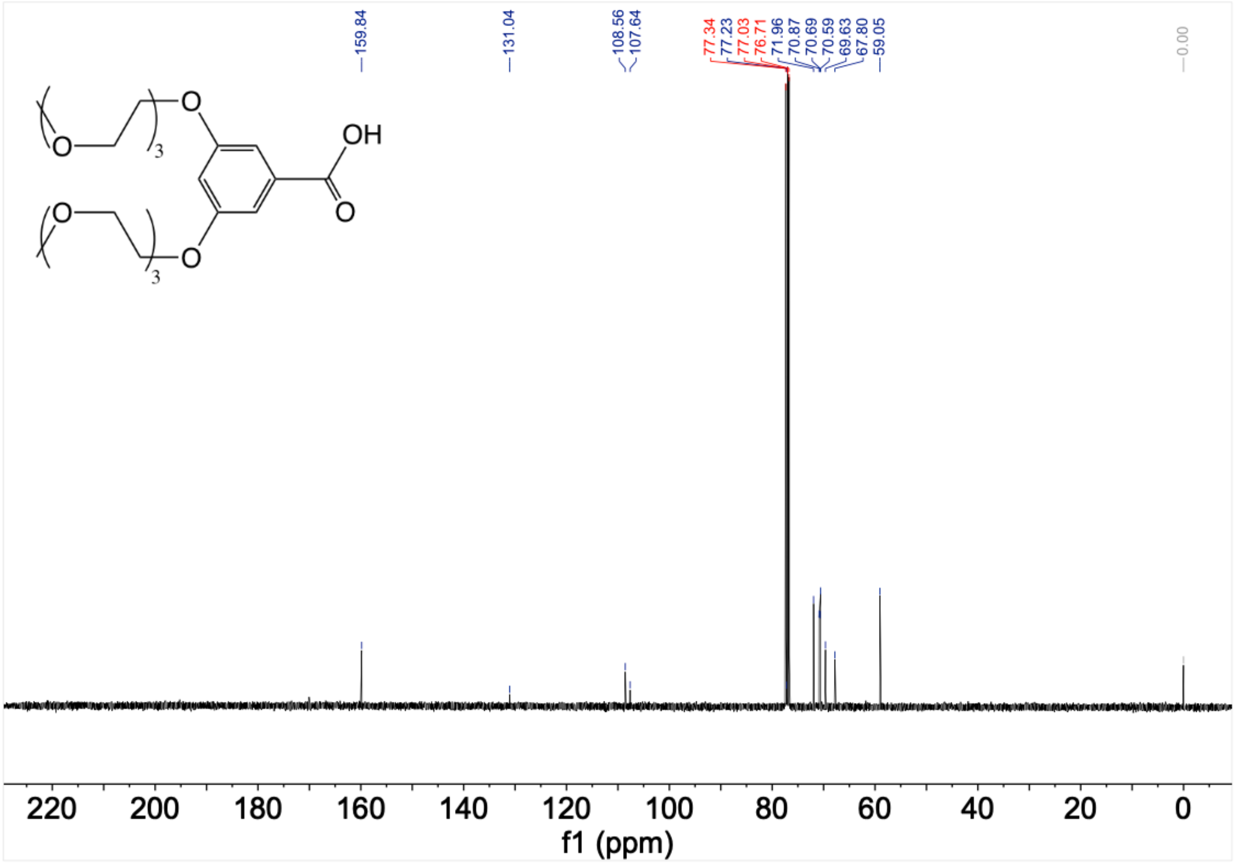

Figure S19. ^{13}C NMR spectrum of **Compound 7** (101 MHz, $CDCl_3$).

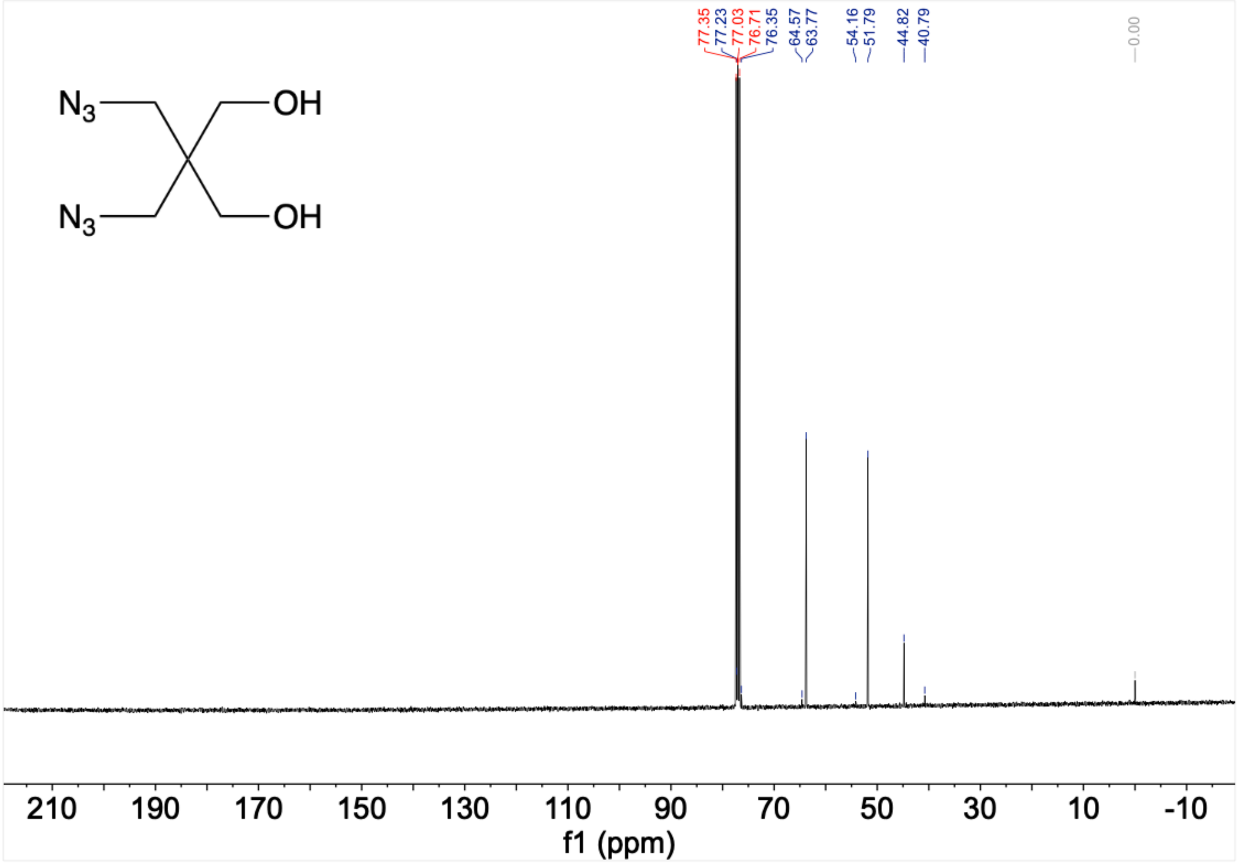

Figure S20. ^{13}C NMR spectrum of **Compound 9** (101 MHz, $CDCl_3$).

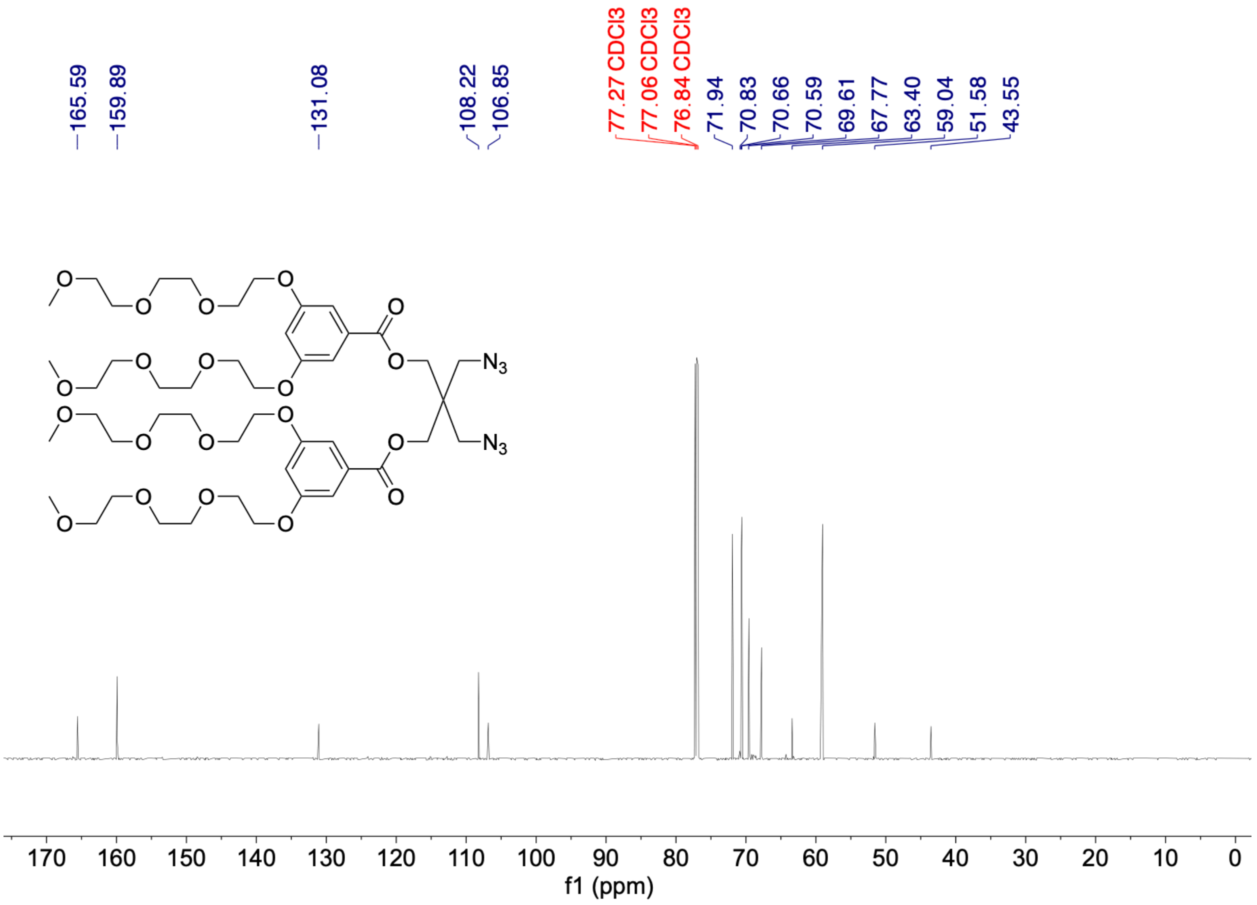

Figure S21. ^{13}C NMR spectrum of **Compound 10** (150 MHz, $CDCl_3$).

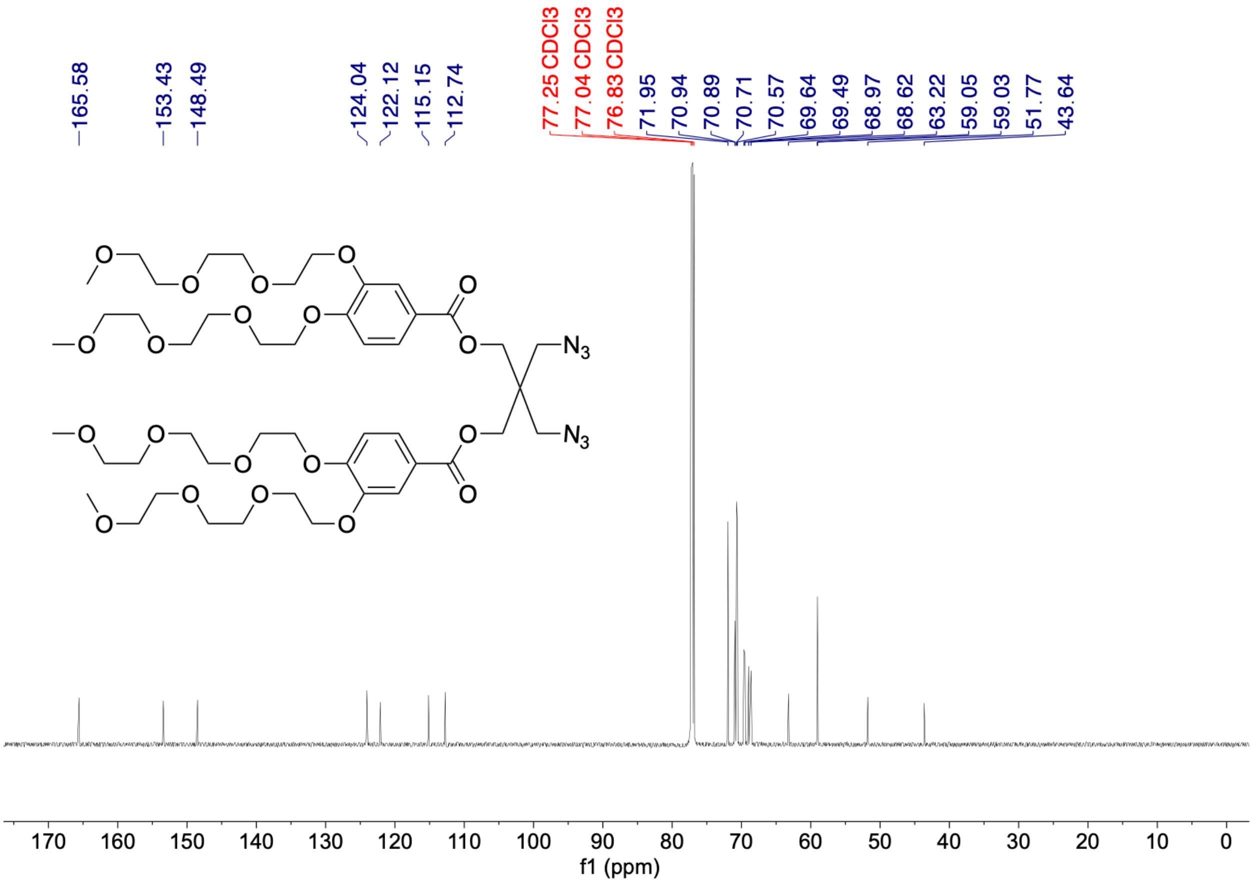

Figure S22. ^{13}C NMR spectrum of **Compound 11** (150 MHz, $CDCl_3$).

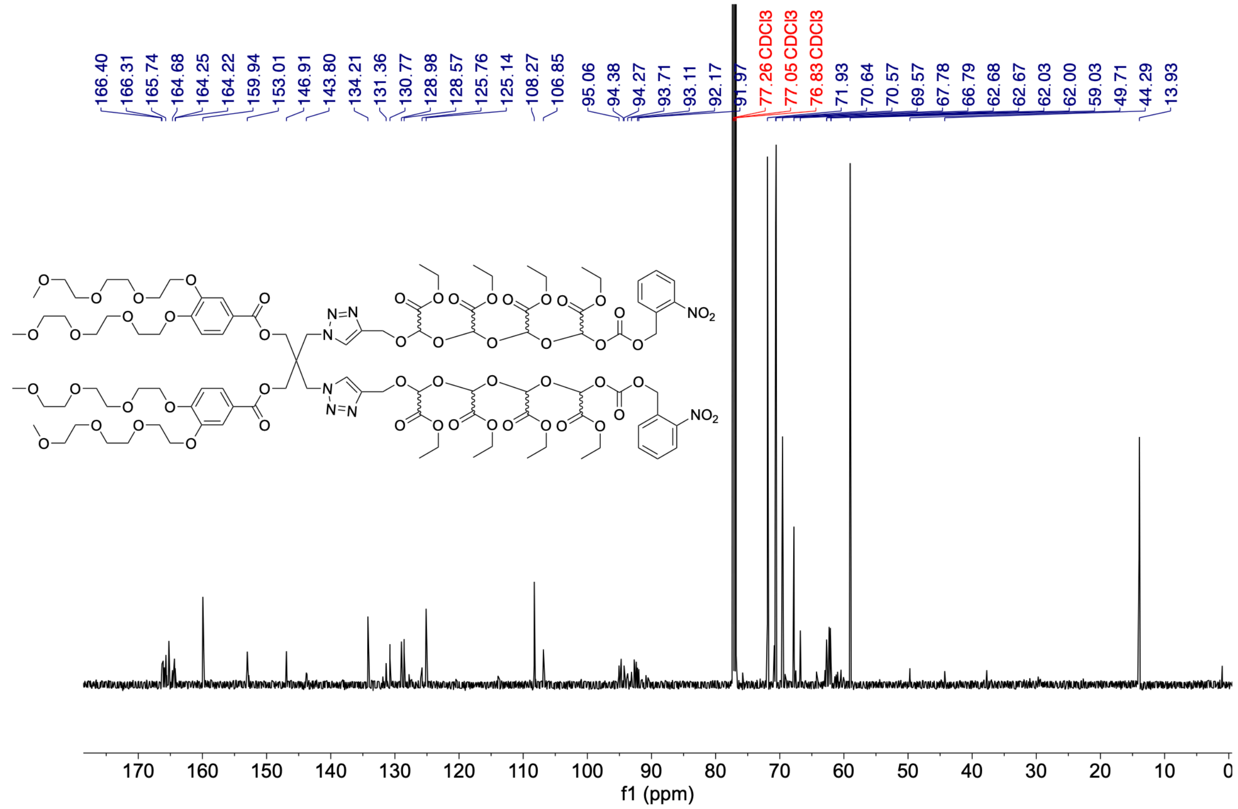

Figure S23. ^{13}C NMR spectrum of **SIJD1** (150 MHz, $CDCl_3$). Note that multiple peaks are present for carbon atoms on and near the oligo(ethyl glyoxylate) backbone due to the presence of stereoisomers (see **Figure S3**).

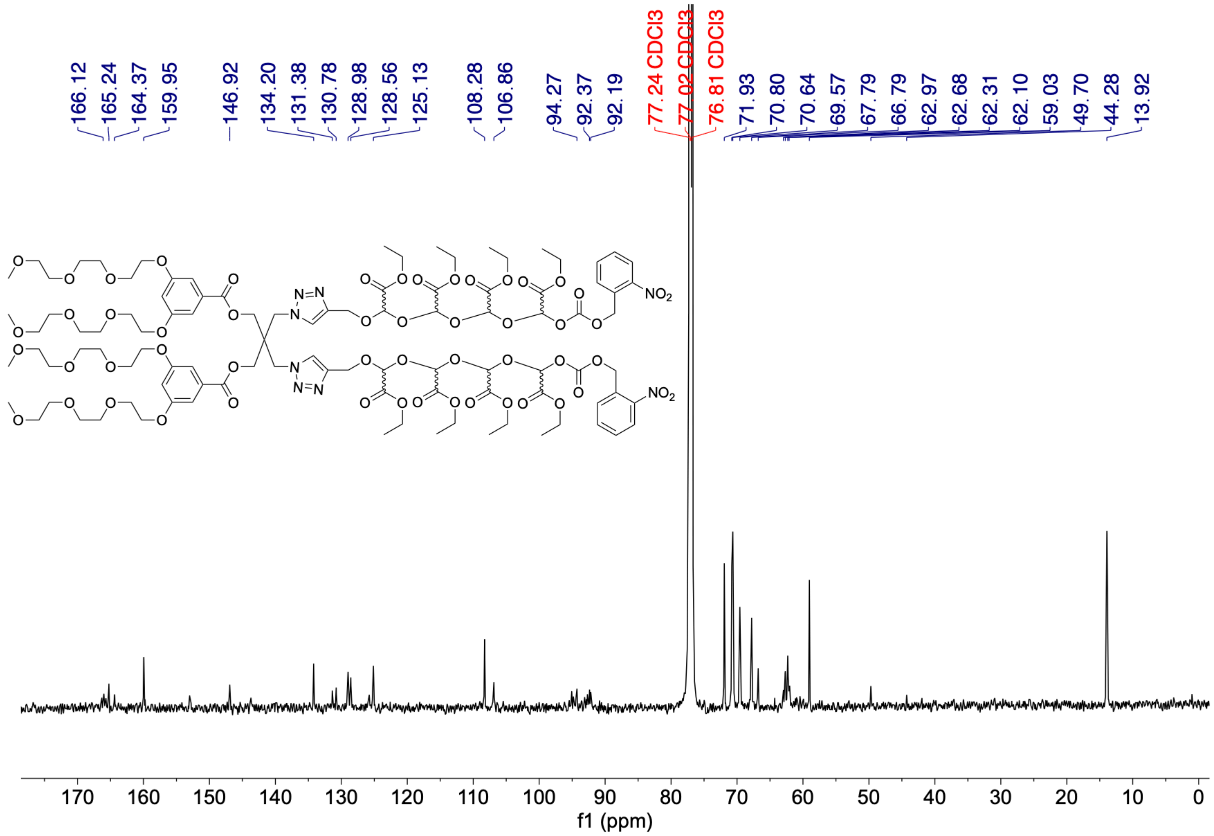

Figure S24. ^{13}C NMR spectrum of **SIJD2** (150 MHz, $CDCl_3$). Note that multiple peaks are present for carbon atoms on and near the oligo(ethyl glyoxylate) backbone due to the presence of stereoisomers (see **Figure S3**).

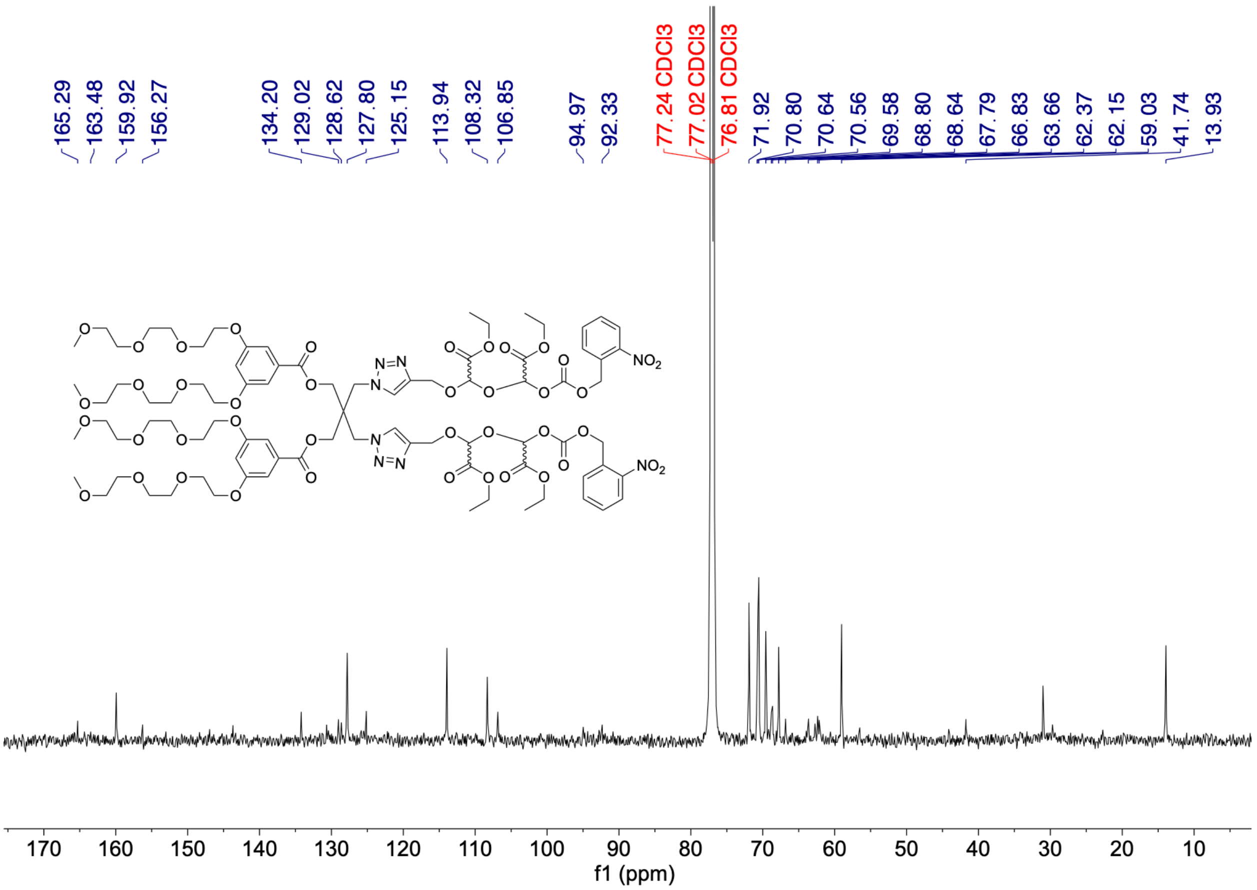

Figure S25. ^{13}C NMR spectrum of **SIJD3** (150 MHz, $CDCl_3$). Note that multiple peaks are present for carbon atoms on and near the oligo(ethyl glyoxylate) backbone due to the presence of stereoisomers (see **Figure S2**).

Mass Spectrometry

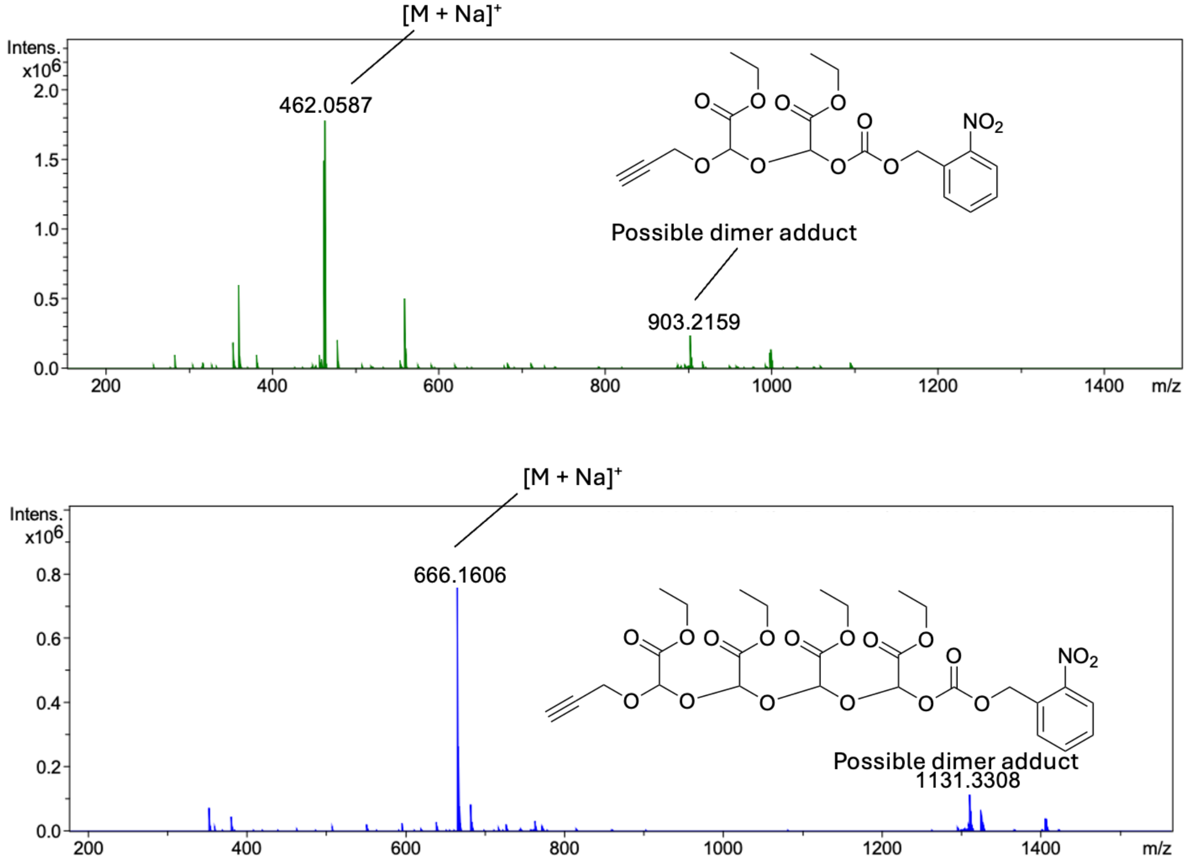

Figure S26. Mass spectra of **Compounds 2** (**a**) and **3** (**b**).

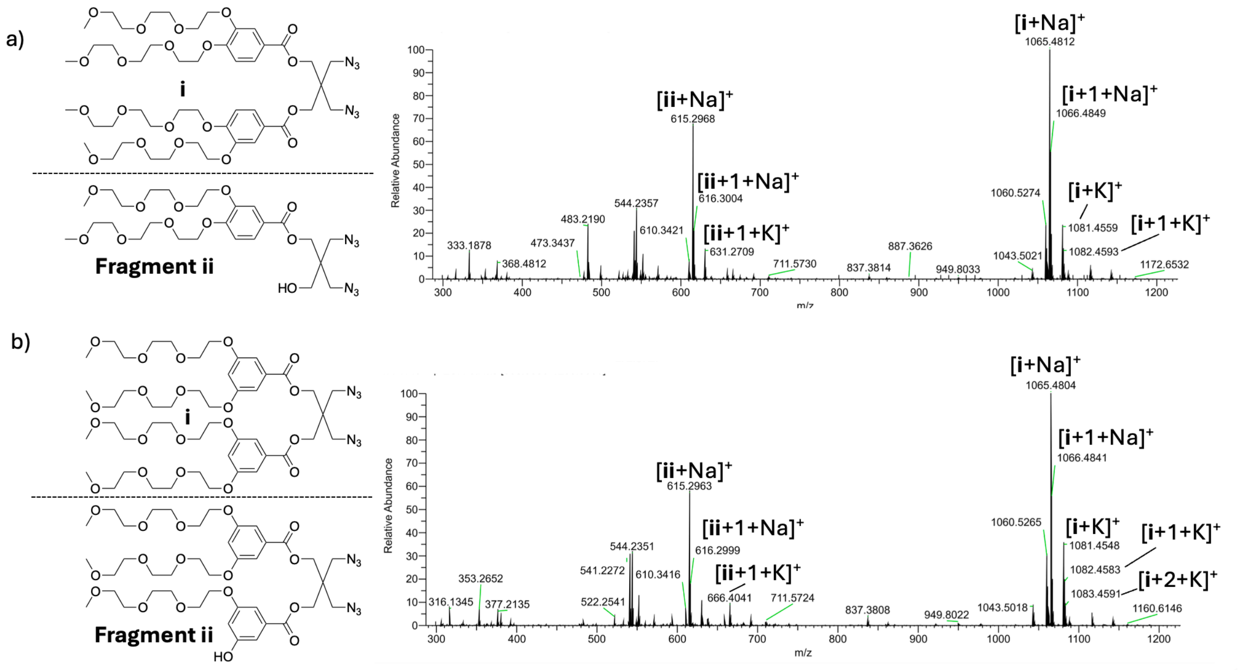

Figure S27. Mass spectra of (**a**) **Compound 10** and (**b**) **Compound 11**. Note that for both compounds, isotopic peaks were observed for ^{13}C, which are denoted as +1 and +2; fragmentations (**ii**) were also observed at 615.2968 and 615.2963 respectively.

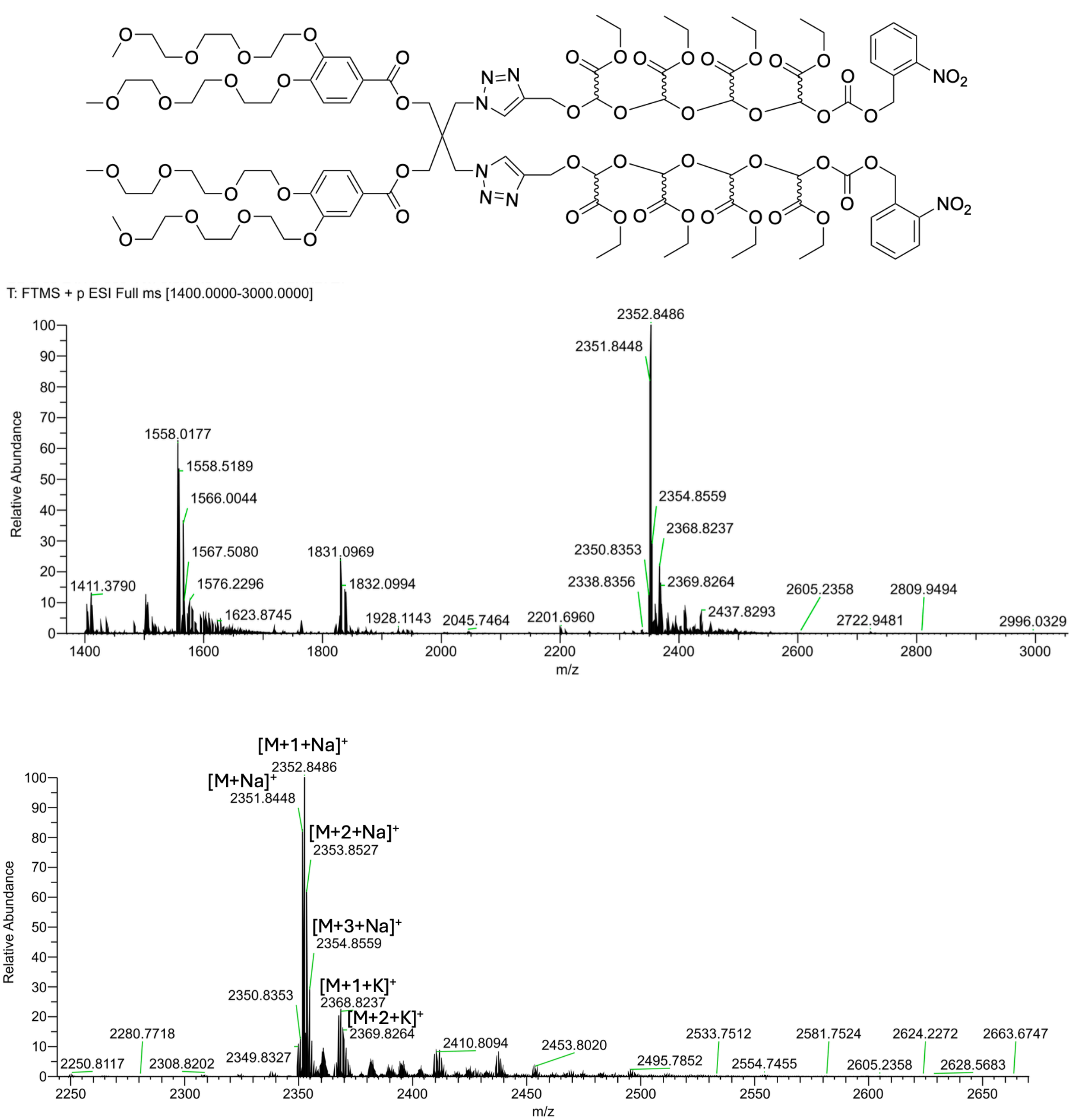

Figure S28. Mass spectrum of **SIJD1** with a zoom of the region from m/z = 2250–2650. Note that isotopic peaks were observed for ^{13}C, which are denoted as +1, +2, etc.

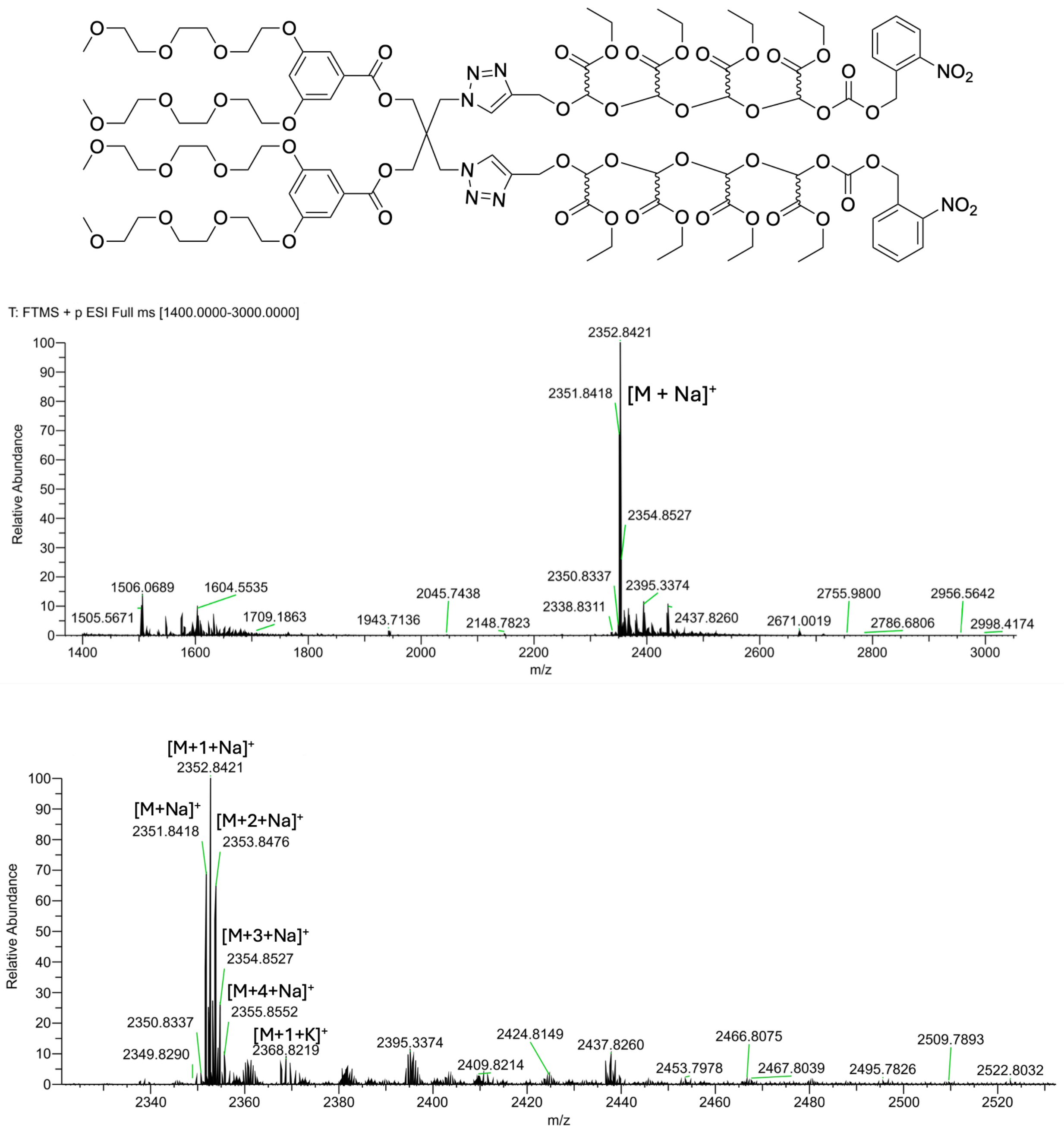

Figure S29. Mass spectrum of **SIJD2** with a zoom of the region from m/z = 2320–2520. Note that isotopic peaks were observed for ^{13}C, which are denoted as +1, +2, etc.

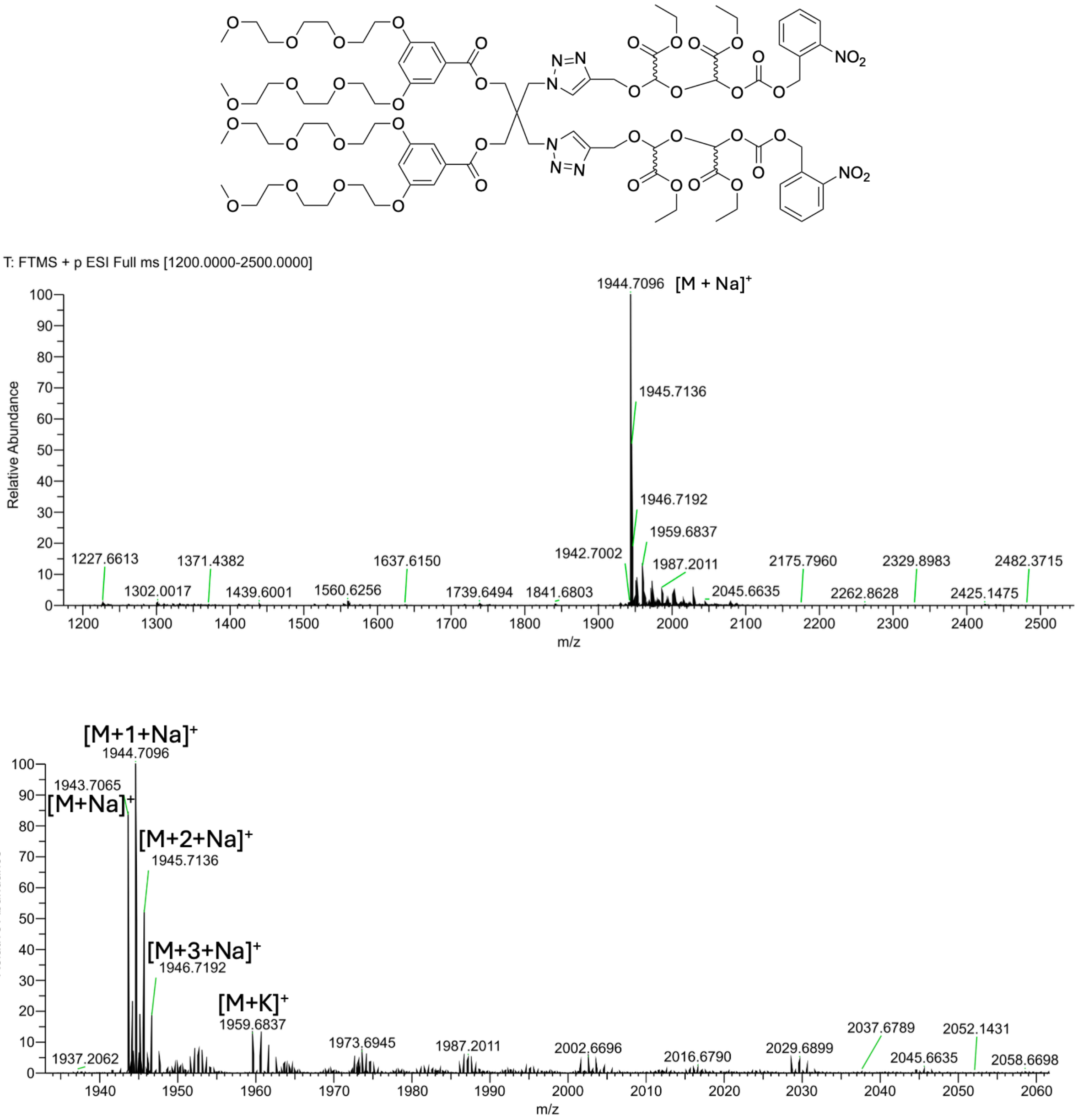

Figure S30. Mass spectrum of **SIJD3** with a zoom of the region from m/z = 1930–2060. Note that isotopic peaks were observed for ^{13}C, which are denoted as +1, +2, etc.

HPLC Data

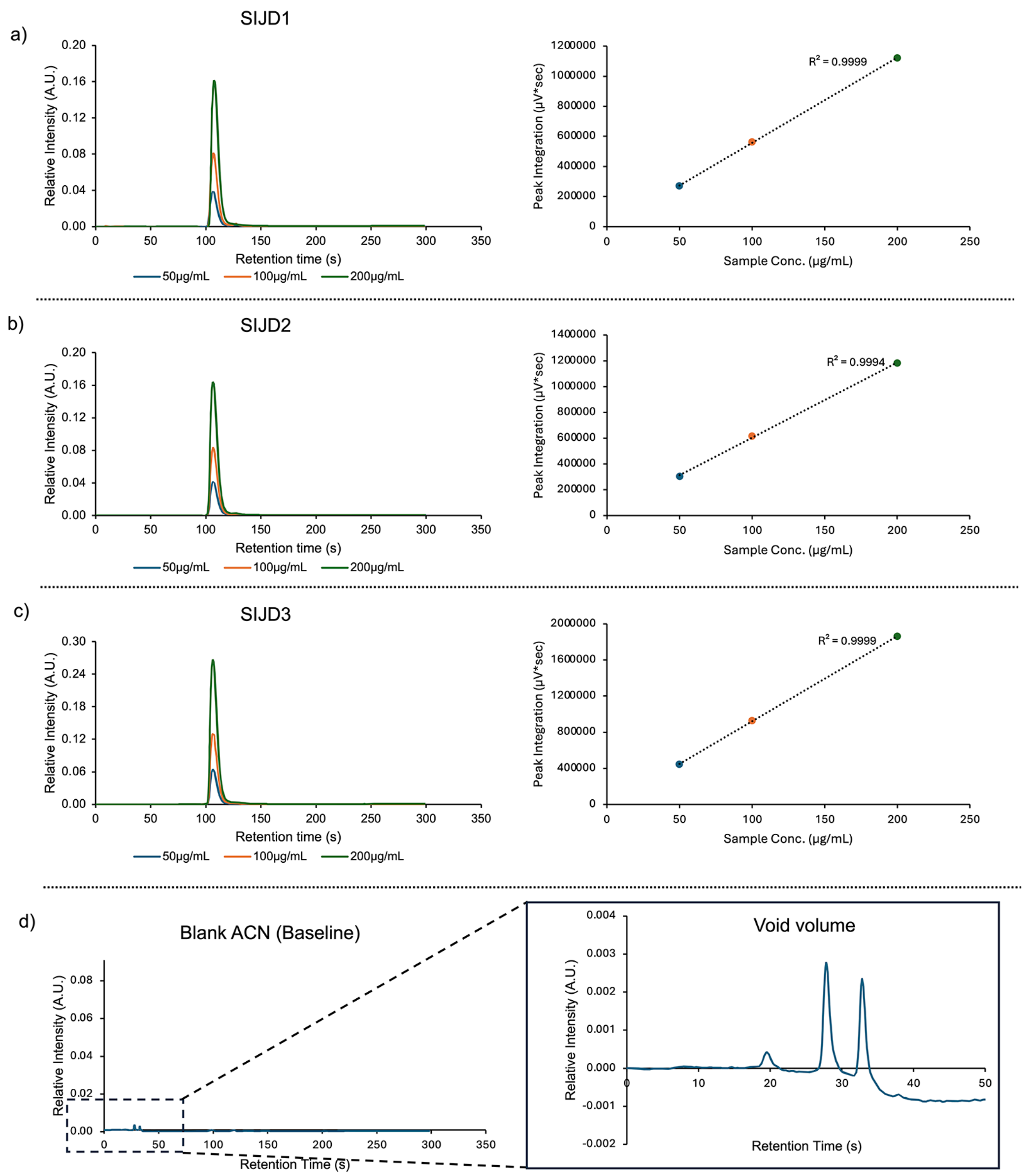

Figure S31. HPLC chromatograms of a) **SIJD1**, b) **SIJD2**, c) **SIJD3** at 50, 100, and 200 μg mL^{-1} in acetonitrile (ACN) (left); and the corresponding concentration–peak area relationships used to verify peak assignments for SIJDs 1−3 (right). d) HPLC chromatogram of the ACN eluent, indicating the void volume, which is clearly separated from the elution of SIJDs. Notes: 1. A minor impurity peak was observed for SIJD samples at an elution time of 126 s. Peak areas were determined for solutions at 50, 100, and 200 μg mL^{-1} using chromatographic integration and were 5327, 8080, and 16980 μV*s for SIJD1; 1174, 1907, and 3666 μV*s for SIJD2; and 2527, 4833, and 11158 μV*s for SIJD3, respectively. Based on the corresponding concentration–peak area calibration curves, the impurity content was estimated to be 1.64 ± 0.29% for SIJD1, 0.34 ± 0.04%

for SIJD2, and 0.56 ± 0.04% for SIJD3. 2. After confirming that no additional peaks were detected beyond those eluting at 107 s (product) and 126 s (impurity) over a 20 min run, subsequent HPLC analyses were performed using a 5 min run time.

Characterization of SIJD Self-assemblies

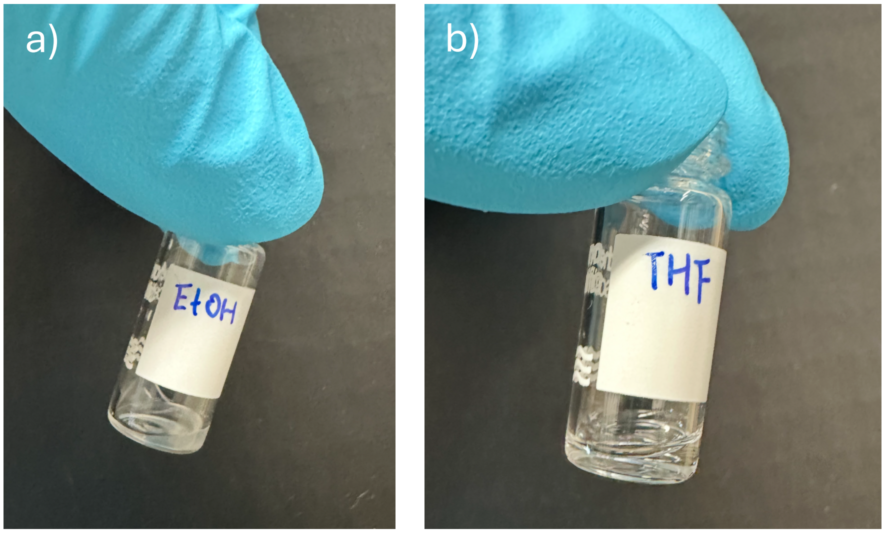

Figure S32. SIJD3 dissolved in EtOH (**a**) and THF (**b**).

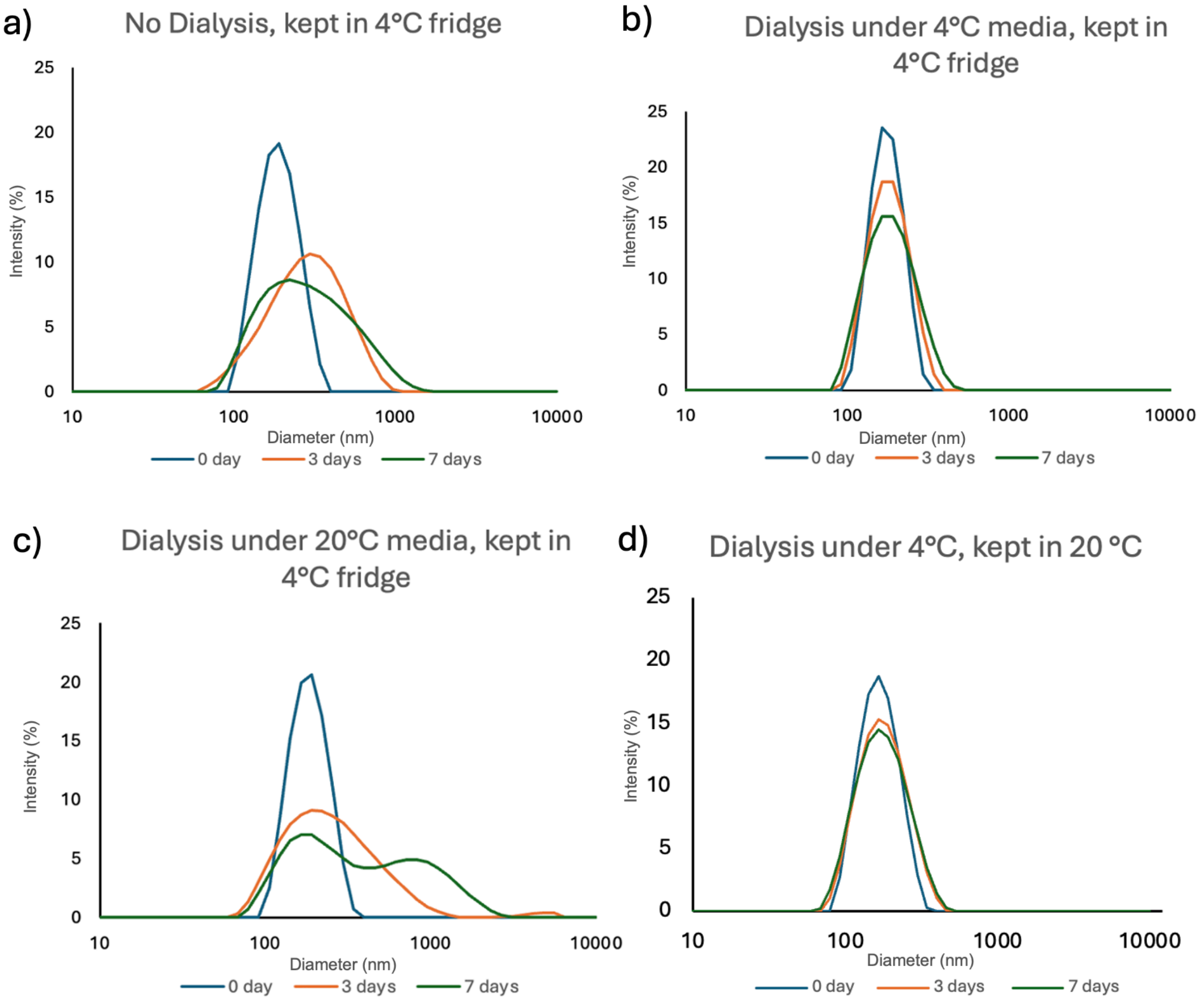

Figure S33. Stability investigation of **SIJD3** self-assemblies as indicated by DLS (intensity distributions) under the following conditions: (**a**) stored in fridge at 4 °C immediately after self-assembly; (**b**) dialyzed in 4 °C water and stored at 4 °C; (**c**) dialyzed in 20 °C water and subsequently stored at 4 °C; (**d**) dialyzed in 4 °C water and stored at 20 °C.

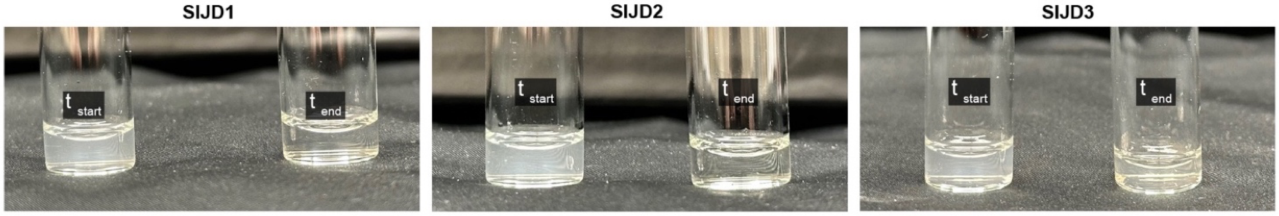

Figure S34. Photos of self-assembled particles in solution before and after UV irradiation showing transitions from translucent to clear.

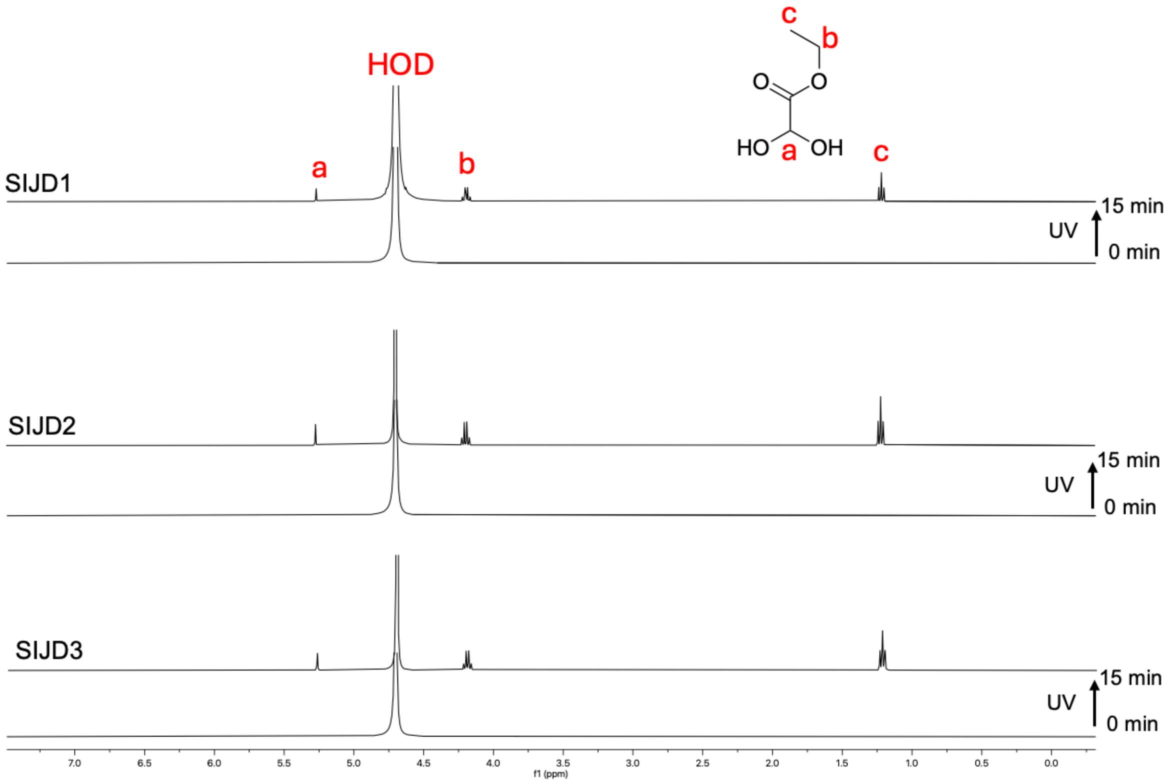

Figure S35. ¹H NMR spectra (600 MHz) of assemblies formed from **SIJD1**, **SIJD2**, and **SIJD3** nanoparticles in D_2O before and after 15 min of 365 nm light irradiation. Prior to irradiation, only the solvent peak (HOD, 4.7 ppm) was observed. After irradiation, new peaks corresponding to the ethyl glyoxylate depolymerization product emerged (labelled in red: a, b, c).

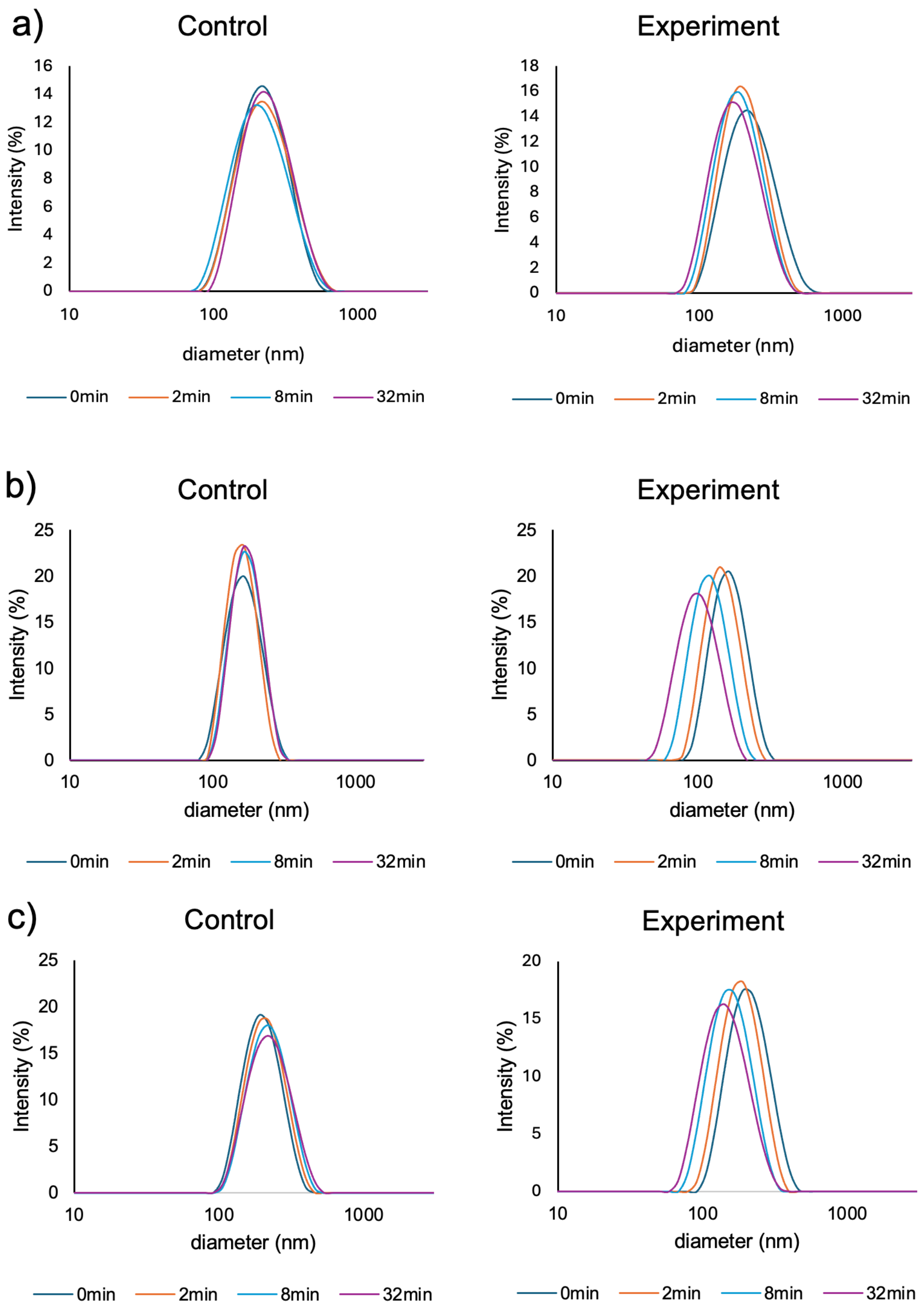

Figure S36. Intensity-weighted diameter distributions over time for: (**a**) **SIJD1**, (**b**) **SIJD2**, and (**c**) **SIJD3**, where the control samples were kept in dark, and experimental samples were exposed under UV irradiation.

Fluorescence Spectra for Nile Red Release

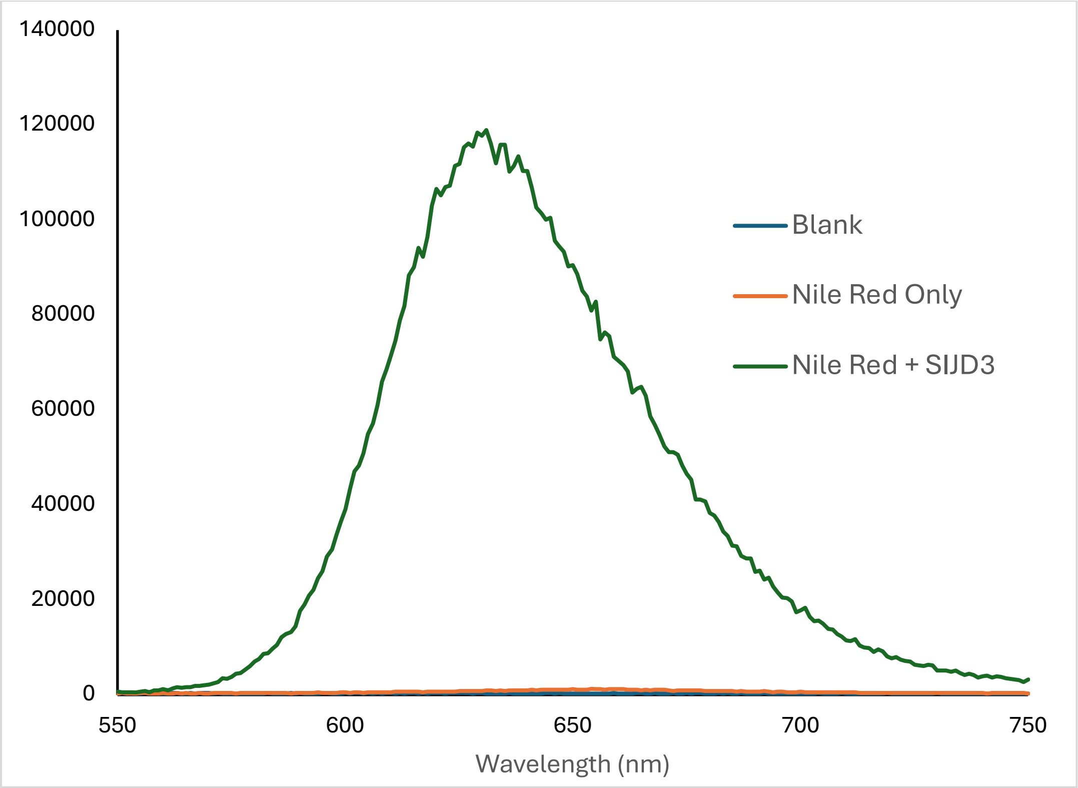

Figure S37. Fluorescence spectra of Nile red-loaded **SIJD3** assemblies and free Nile red, prepared using the same self-assembly procedure (blank is water alone).

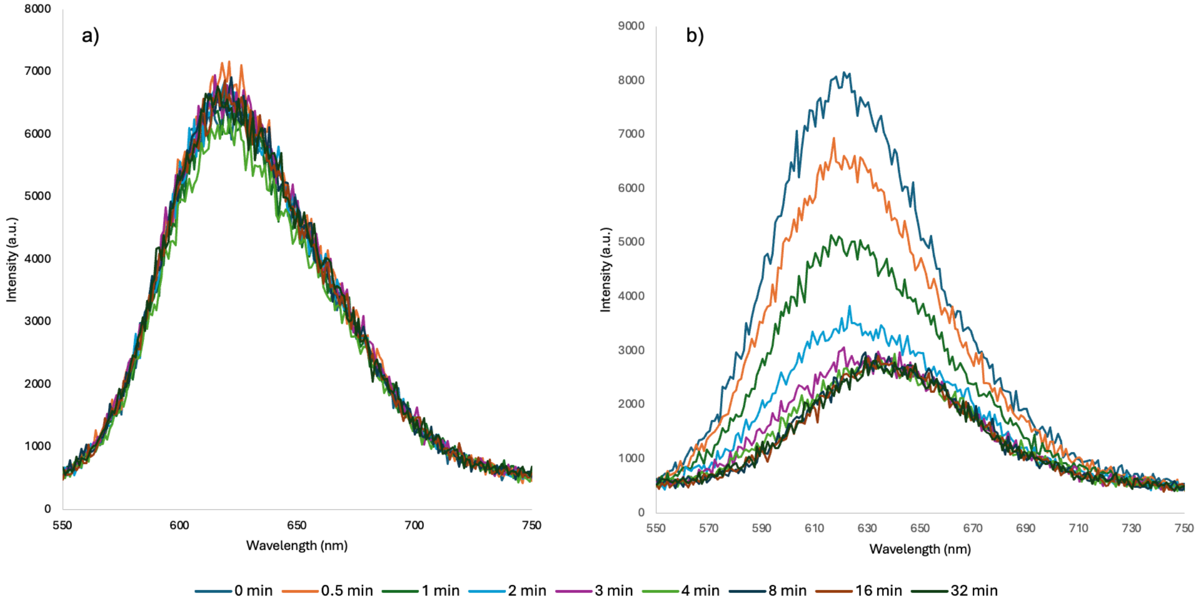

Figure S38. Fluorescence spectra of Nile red-loaded **SIJD1** nanoparticles: (**a**) kept in the dark and (**b**) irradiated with UV light for 32 min. Samples kept in the dark remained stable, with no change in fluorescence intensity. In contrast, UV-irradiated samples showed a gradual decrease in fluorescence, which plateaued after approximately 4 minutes of cumulative irradiation. A slight red shift of the emission peak was observed, attributed to changes in the local environment of the dye (polarity shift).

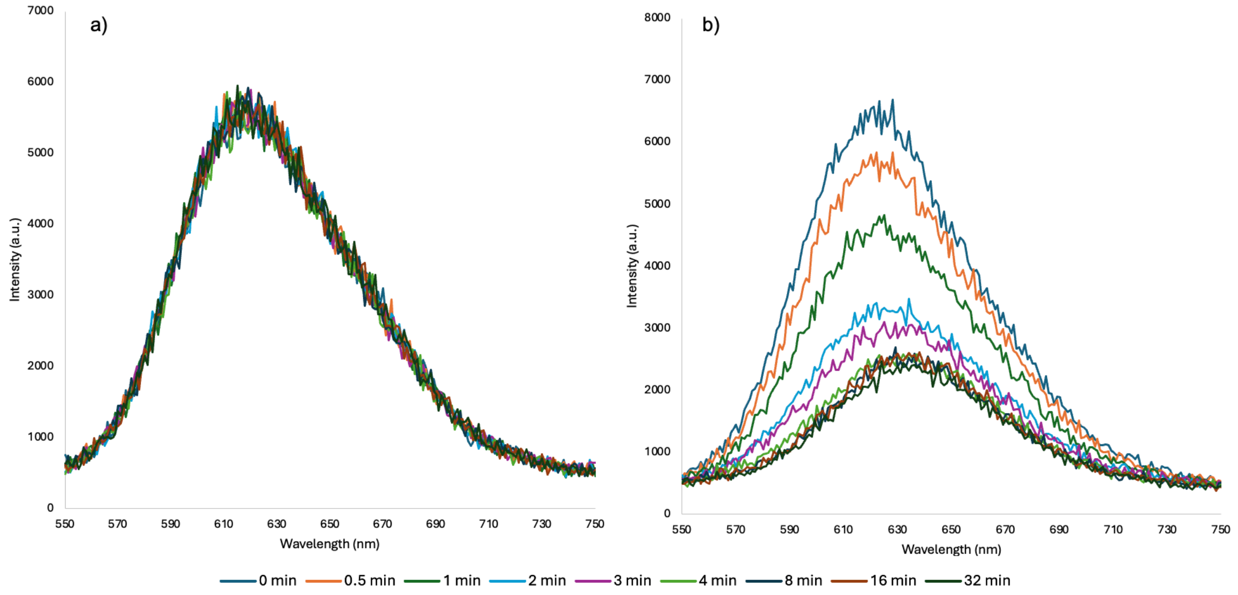

Figure S39. Fluorescence spectra of Nile red-loaded **SIJD2** nanoparticles: (**a**) kept in the dark and (**b**) irradiated with UV light for 32 min. Samples kept in the dark remained stable, with no change in fluorescence intensity. In contrast, UV-irradiated samples showed a gradual decrease in fluorescence, which plateaued after approximately 4 minutes of cumulative irradiation. A slight red shift of the emission peak was observed, attributed to changes in the local environment of the dye (polarity shift).

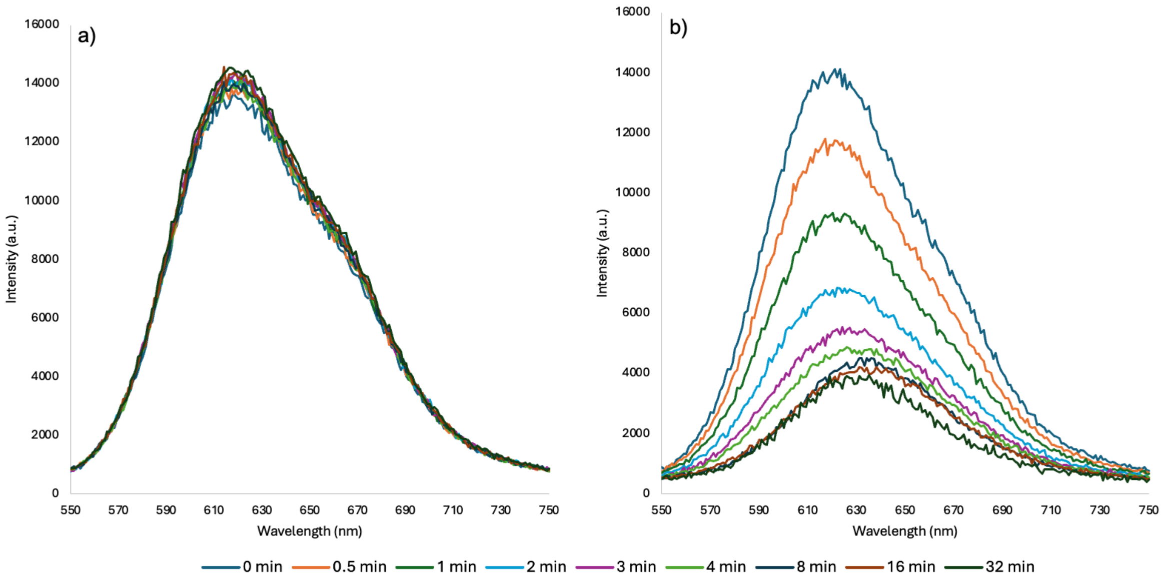

Figure S40. Fluorescence spectra of Nile red-loaded **SIJD3** nanoparticles: (**a**) kept in the dark and (**b**) irradiated with UV light for 32 min. Samples kept in the dark remained stable, with no change in fluorescence intensity. In contrast, UV-irradiated samples showed a gradual decrease in fluorescence, which plateaued after approximately 4 minutes of cumulative irradiation. A slight red shift of the emission peak was observed, attributed to changes in the local environment of the dye (polarity shift).

References

(1) Rabiee Kenaree, A.; Gillies, E. R. Controlled Polymerization of Ethyl Glyoxylate Using Alkyllithium and Alkoxide Initiators. *Macromolecules* **2018**, *51*, 5501-5510.

(2) Percec, V.; Wilson, D. A.; Leowanawat, P.; Wilson, C. J.; Hughes, A. D.; Kaucher, M. S.; Hammer, D. A.; Levine, D. H.; Kim, A. J.; Bates, F. S.; Davis, K. P.; Lodge, T. P.; Klein, M. L.; DeVane, R. H.; Aqad, E.; Rosen, B. M.; Argintaru, A. O.; Sienkowska, M. J.; Rissanen, K.; Nummelin, S.; Ropponen, J. Self-Assembly of Janus Dendrimers into Uniform Dendrimersomes and Other Complex Architectures. *Science* **2010**, *328*, 1009-1014.

(3) Percec, V.; Leowanawat, P.; Sun, H.-J.; Kulikov, O.; Nusbaum, C. D.; Tran, T. M.; Bertin, A.; Wilson, D. A.; Peterca, M.; Zhang, S.; Kamat, N. P.; Vargo, K.; Moock, D.; Johnston, E. D.; Hammer, D. A.; Pochan, D. J.; Chen, Y.; Chabre, Y. M.; Shiao, T. C.; Bergeron-Brlek, M.; André, S.; Roy, R.; Gabius, H.-J.; Heiney, P. A. Modular Synthesis of Amphiphilic Janus Glycodendrimers and Their Self-Assembly into Glycodendrimersomes and Other Complex Architectures with Bioactivity to Biomedically Relevant Lectins. *J. Am. Chem. Soc.* **2013**, *135*, 9055-9077.

(4) Moore, J. S.; Stupp, S. I. Room temperature polyesterification. *Macromolecules* **1990**, *23*, 65-70.

(5) Shi, S.; Yao, C.; Cen, J.; Li, L.; Liu, G.; Hu, J.; Liu, S. High-fidelity End-functionalization of Poly(ethylene glycol) using stable and potent carbamate linkages. *Angew. Chem. Int. Ed.* **2020**, *59*, 18172-18178.
